# The Natural History of Class I Primate Alcohol Dehydrogenases Includes Gene Duplication, Gene Loss, and Gene Conversion

**DOI:** 10.1371/journal.pone.0041175

**Published:** 2012-07-31

**Authors:** Matthew A. Carrigan, Oleg Uryasev, Ross P. Davis, LanMin Zhai, Thomas D. Hurley, Steven A. Benner

**Affiliations:** 1 Foundation for Applied Molecular Evolution, Gainesville, Florida, United States of America; 2 Department of Biochemistry and Molecular Biology, Indiana University School of Medicine, Indianapolis, Indiana, United States of America; University of Lausanne, Switzerland

## Abstract

**Background:**

Gene duplication is a source of molecular innovation throughout evolution. However, even with massive amounts of genome sequence data, correlating gene duplication with speciation and other events in natural history can be difficult. This is especially true in its most interesting cases, where rapid and multiple duplications are likely to reflect adaptation to rapidly changing environments and life styles. This may be so for Class I of alcohol dehydrogenases (*ADH1s*), where multiple duplications occurred in primate lineages in Old and New World monkeys (OWMs and NWMs) and hominoids.

**Methodology/Principal Findings:**

To build a preferred model for the natural history of *ADH1s*, we determined the sequences of nine new *ADH1* genes, finding for the first time multiple paralogs in various prosimians (lemurs, strepsirhines). Database mining then identified novel *ADH1* paralogs in both macaque (an OWM) and marmoset (a NWM). These were used with the previously identified human paralogs to resolve controversies relating to dates of duplication and gene conversion in the *ADH1* family. Central to these controversies are differences in the topologies of trees generated from exonic (coding) sequences and intronic sequences.

**Conclusions/Significance:**

We provide evidence that gene conversions are the primary source of difference, using molecular clock dating of duplications and analyses of microinsertions and deletions (micro-indels). The tree topology inferred from intron sequences appear to more correctly represent the natural history of *ADH1s*, with the *ADH1* paralogs in platyrrhines (NWMs) and catarrhines (OWMs and hominoids) having arisen by duplications shortly predating the divergence of OWMs and NWMs. We also conclude that paralogs in lemurs arose independently. Finally, we identify errors in database interpretation as the source of controversies concerning gene conversion. These analyses provide a model for the natural history of *ADH1*s that posits four ADH1 paralogs in the ancestor of Catarrhine and Platyrrhine primates, followed by the loss of an ADH1 paralog in the human lineage.

## Introduction

While genomics has opened new directions in biological and biomedical research, its surfeit of data also makes evident the complexity of real genomes and their evolution, a complexity that challenges our ability to interpret the natural history of biomolecules set within the natural history of their host organisms. A special kind of challenge is presented when a genome “contains “too many” paralogous genes encoding proteins that appear to be duplicate catalysts for “the same reaction” [1]. Here, functional genomics seeks to construct hypotheses to explain how such paralogs differentially contribute to fitness.

Those hypotheses cannot ignore events in divergent evolution that are not modeled well by standard bioinformatics tools. Handled well by these tools are stochastic amino acid replacements with occasional insertions and deletions (indels) placed parsimoniously on trees. Here, the phylogeny of all fragments in a gene sequence are expected to fit the same phylogeny and, especially, the phylogeny of their host organisms. Handled less well are events that do not conform to canonical processes for sequence evolution. These include gene conversion [2], multiple indels not stochastically distributed in a sequence [3], compensatory covariation [4], convergent evolution, and other forms of homoplasy [5]. Standard bioinformatics tools can also be easily confused by database error [6], including incomplete or faulty gene finding. Studies of the natural history of individual gene families must consider all of these. Therefore, many investigators complement standard bioinformatics tools with studies of these processes in individual protein families [7]. By comparing many individual “case studies” over time, hypotheses to explain the creation, maintenance, and retention of “too many” paralogs should become easier to formulate. These can then be tested using paleogenetics, an experimental approach in which sequence data from contemporary species are used to predict the ancestral protein sequence at key points in the evolutionary history of a protein family. These ancestral protein sequences are then resurrected by *in vitro* techniques, and subjected to experimental studies in the laboratory [8]–[10].

Alcohol dehydrogenases (ADHs) offer an excellent example of the extent to which models for natural history can become convoluted by combinations of such evolutionary processes. This makes them systems well suited to develop case studies that combine canonical and non-canonical models with paleogenetic experiments. Indeed, ADH paralogs from yeast, which produce much of the ethanol on Earth, have already been the target of paleogenetic resurrections based on canonical and non-canonical bioinformatics analyses [11]. These resurrections brought 80 million year old ancestral fungal ADHs into the laboratory where they were studied to test hypotheses explaining why yeast holds two ADH paralogs, and how they relate to changing behavior and function in a changing environment [12].

Yeast ADH has clear homologs in mammals. In addition to more distant oxidoreductase relatives that contain the “Rossmann fold” [13], these mammalian homologs include five “canonical” classes of ADHs that are especially closely related [14]. Following suggestions of Duester *et al.* [15], the genes within the five evolutionary classes are designated *ADH1* through *ADH5*. Different canonical ADH classes appear to have been adapted to oxidize species other than ethanol. For example, formaldehyde (HCHO) is a likely target of *ADH3* [16]–[18]. However, in humans and other mammals, representative members of *ADH1* and *ADH4* appear to act well on ethanol, oxidizing it to acetaldehyde, which is then oxidized to acetate.

In general, only one exemplar of each class of ADH is reported in mammalian species. For example, Class I ADH (*ADH1)* has a single exemplar in the tree shrew (representing the mammal order *Scandentia*), one in mouse and gopher (representing *Rodentia*), one in cow and pig (representing *Artiodactyla*), and one in dog and panda (representing *Carnivora*). Only occasionally is more than one Class I *ADH* paralog observed in a mammalian genome. For example, horse (representing *Perissodactyla*) has two forms of *ADH1*, differing in only 10 of 375 sites and apparently arising from a recent duplication [19]. While southern blot hybridization studies suggested two forms of *ADH1* were present in rabbit (*Oryctolagus cuniculus*) and sheep (*Ovis aries)* [20], a recent search of the NCBI databases found no sequence data supporting a second *ADH1* gene.

**Figure 1:**
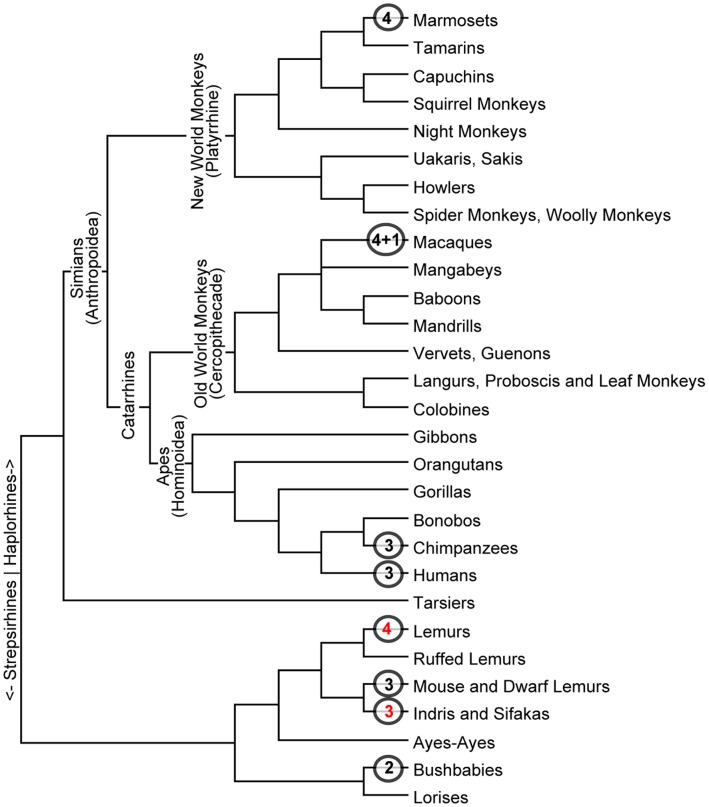
Overview of primate phylogeny. An overview of primate phylogeny is shown, with the number of *ADH1* paralogs identified within select taxon indicated by the circled numbers at the leaves of the tree. Black numbers are derived from analysis of public databases, while red numbers were determined from cDNA sequencing reported here. The “4+1” designation for the macaque taxon indicates the presence of four *ADH1* paralogous genes plus one *ADH1* pseudogene. The genome sequencing projects are not completed for any lemur, so additional *ADH1* paralogs may be present (see text).

Primates also have single exemplars of *ADH2*, *ADH3*, *ADH4*, and *ADH5*. However, many primates have multiple paralogs for *ADH1*. For example, in *Homo sapiens*, representing the catarrhines (which includes Old World monkeys and hominoids/apes, see Figure 1), three Class I ADH paralogs are found, and are designated *ADH1A*, *ADH1B*, and *ADH1C* [21] [15]. New World platyrrhines, represented by the marmoset, also have multiple *ADH1* paralogs [this work]. Similarly, strepsirhines (lemurs), primates that diverged from Old and New World primates (collectively, the haplorhines) about 63–90 million years ago (MYA) [22]–[24] also have multiple *ADH1* paralogs [this work].

Many attempts have been made to model the natural history of primate *ADH1* in the face of multiple paralogs in many primate species. For example, Ikuta *et al.* [25] constructed consensus sequences to argue that *ADH1A* diverged about 10 million years ago from the lineage that later diverged to create *ADH1B* and *ADH1C*. The divergence was characterized by a rate constant of 0.5×10^−9^ substitutions/site/year (see also the of 0.5×10^−9^ substitutions/site/year rate constant reported by [26]). Yokoyama and Yokoyama disagreed [27]. Based on amino acid sequences from both metazoan and plant ADHs, they placed human *ADH1C* as an outgroup with respect to *ADH1B* and *ADH1A*. In contrast, Yokoyama *et al.* [28] suggested that *ADH1B* might be the outgroup. Yokoyama and Harry [29] returned to preferring *ADH1C* as the outgroup, but remarked that “the support for clustering … is very low, and it may be more appropriate to conclude that the three subunits diverged about the same time. ” In yet another pre-genomic study, Trezise *et al.* [26], [30] expanded the analysis to include the coding region of a single *ADH1* from baboon (*Papio hamadryas*). Applying tools that corrected for multiple substitutions at single sites, they (like Ikuta *et al.* [25]) placed *ADH1*A as an outgroup, but found a synonymous substitution rate constant of *1.2*×10^−9^ substitutions/site/year.

As the post-genomic era developed, Cheung *et al.* [31] revisited the problem, sequencing segments of the 5′-untranslated regions (5′-UTR) of various *ADH1* genes from baboon and macaque (*Macaca mulatta*, an Old World monkey), comparing their sequences with the sequence of genes from human. They found evidence for “reticulate” evolution, which they interpreted as evidence of gene conversion. This raised the possibility that the outgroup-ingroup relationship of *ADH1A*, *ADH1B*, and *ADH1C* might not be the same across the entire length of the gene, with at least three regions of the gene (908-nt of the 5′-UTR, exon 2–5, and from exon 7 to 271-nt into the 3′-UTR) evolving differently.

A decade later, Oota *et al.* [32] revisited the problem after the human genome sequence was complete. Adding 15 kb of new sequence data from four hominoids (*Pan troglodytes,* chimpanzee; *Pan paniscus,* bonobo; *Gorilla gorilla,* gorilla; and *Pongo pygmaeus,* orangutan) and one Old World monkey (*Papio anubis,* baboon) covering introns 2, 3, and 8 at the *ADH1* locus, they analyzed their data in the context of other intronic sequence data [33]. These included the orthologous introns from *H. sapiens*. Like Yokoyama *et al.* [27] they concluded that *ADH1C* was the outgroup for *ADH1A* and *ADH1B*. Contradicting Cheung *et al.* [31], however, Oota *et al.* found no evidence for gene conversion [32].

Patterns in the divergent evolution of *ADH1* display various signatures of adaptation, further complicating simple models for sequence evolution. For example, heterotachyous sites [34], those whose rates of change are different in Class I ADHs compared to other classes of ADHs that have not undergone adaptive change, are clustered in the active site [35]. This can be taken as evidence of adaptation in cases where the *d*_N_/*d*_S_ ratio does not surpass unity [36]. Another signature that can indicate functional adaptation that might confound simple bioinformatics tools is an unexpectedly high number of sites that show parallel or reverse evolution (homoplasy) during the divergent evolution of *ADH1* paralogs.

While the specific physiological roles of the various ADH classes remain unclear, the involvement of *ADH4* and the multiple *ADH1* paralogs in ethanol metabolism demonstrate that the evolution of the ADH gene family has significant implications to human health [37], [38]. Understanding the evolution of this gene family is important to understanding specific details regarding primate adaptation to ethanol, and more generally about the processes of adaptation via gene duplication. Paleogenetics experiments require as their starting point a reasonably robust model for the natural history of a protein family [39]. With their uncertainties surrounding tree topology, homoplasy, and gene conversion, current views of the history of the *ADH1* class of proteins are clearly inadequately robust for such experiments. Accordingly, we sequenced cDNA from liver from a variety of primate taxa, combined these with data obtained from sequence databases (Table S1), and reanalyzed the natural history of *ADH1* using models that might detect gene conversion and functional adaptation. These results are reported here.

## Methods

### ADH1 gene discovery

#### Database mining

A curated database of human, macaque and marmoset *ADH1* genes (including both exons and introns), as well as representative *ADH1* genes from other mammals, was compiled by mining the NCBI Trace Archive whole-genome shotgun (WGS) sequence database and the NCBI non-redundant nucleotide (nr/nt) database (the online October 2009 versions), the ENSEMBLE (version 57) database, and the genome database of *Callithrix jacchus* (downloaded from the genome.ucsc.edu website, March 2009 version WUGSC 3.2/calJac3). Sequences were retrieved using BLAST using human *ADH1*A, *ADH1B* and *ADH1C* DNA sequences as probes. Table S1 holds a list of the *ADH1* genes used here. The Ensemble database annotated the locus containing the *ADH1* genes in macaque as a *single* gene with ten *predicted* splice variants, only four of which were non-overlapping (Figure S1); the four non-overlapping variants were considered authentic genes.

#### Transcriptome analysis via cDNA Sequencing

Supplementing the sequences of primate ADHs mined from various databases, additional genes from a variety of unrepresented primates were sequenced via RT-PCR of RNA extracted from well-preserved samples of liver tissues (Table S2). Ethical standards for the treatment of animals was ensured by the institutions providing animal tissue (listed in Table S2) according to the ethical review board policy of each institution, including the Duke Lemur Center Research Committee, the Institutional Animal Care and Use Committee of the University of Alabama at Birmingham, the Duke University Animal Care and Use Committee, the Wildlife Conservation Society Biomaterials Committee and the Wildlife Conservation Society Animal Management Committee, the Institutional Animal Care and Use Committee of the University of Wisconsin, and the Institutional Animal Care and Use Committee of the Southwest National Primate Research Center. In general, tissues were collected at necropsy following natural death, or were collected initially for research other than that described here. RNA was extracted from these tissues using TRI reagent, with minor modifications to manufacturers protocol (Sigma).

In general, frozen tissue (ca. 0.1 gram) was placed in a screw cap tube (2.0 mL) containing Trizol (1.0 mL) and zirconia/silica beads (0.2 mL, 1.0 mm, 3.7 g/cc; Research Products International). The tube containing tissue, beads and Trizol was attached to the blade of reciprocating saw (Craftsman model 172.171040, 6.5 amp, 150 mm path length) and homogenized by shaking at full speed (2400 spm) for 20-seconds, followed by a 20-second pause to prevent overheating. Homogenization via reciprocated mixing was repeated until all tissue was completely dispersed (usually requiring five 20-second mix cycles). The homogenized tissue was transferred to a new tube and mRNA extracted using the manufacturer’s protocol (Sigma, http://www.sigmaaldrich.com/etc/medialib/docs/Sigma/Bulletin/t9424bul.Par.0001.file.tmp/t9424bul.pdf ).

The mRNA was then reverse transcribed at 55°C using Thermoscript (Invitrogen) and gene-specific reverse primers designed against 3′-UTR regions conserved among all known primate *ADH1* sequences. The primers used are listed in Table S3. PCR was then performed using hot start Taq polymerase (Sigma) using the same reverse primers as used in the RT reaction, with a forward primer designed against conserved 5′-UTR regions of primate *ADH1* genes (Table S3). PCR products were gel purified and cloned directly into the TOPO-TA cloning vector (Invitrogen).

Sequencing was performed using Big Dye technology by BioBasic (Markham, Ontario). All genes were sequenced in the forward and reverse directions with at least 100-nt of overlap. At least five independently obtained clones were required to call a potentially unique gene.

To determine if all four *ADH1* paralogs were actively expressed in marmoset (labeled with the prefix “Cal_ADH”, for *Callithrix*), mRNA was extracted from adult liver tissue, converted into cDNA using RT-PCR using primers targeted to the 5′- and 3′-untranslated regions (UTR) conserved among all paralogs, and then cloned. Sequencing of multiple clones identified 20 cDNA clones derived from each of the Cal_*ADH1.1* and Cal_*ADH1.3* paralogs. No clones were recovered for Cal_*ADH1.2* and Cal_*ADH1.4* from adult marmoset liver even when using primers “specific” for these paralogs; these found only more Cal_*ADH1.1* and Cal_*ADH1.3* sequences based on mismatched priming in the RT-PCR.

In search of evidence that the Cal_*ADH1.2* and Cal_*ADH1.4* genes generated transcripts in *some* marmoset tissue, RT-PCR was repeated using fetal marmoset liver mRNA extracts. To increase the likelihood of sequencing rare copies of Cal_*ADH1.2* or Cal_*ADH1.4*, these RT-PCR products were digested with either KpnI (which cuts all marmoset *ADH1* genes except Cal_*ADH1.2*) or with BstI (which cuts all marmoset *ADH1* genes except Cal_*ADH1.4*). In both cases, at least 75% of the PCR product was cut. This suggested that Cal_*ADH1.1* and Cal_*ADH1.3* transcripts constitute over 50% of the *ADH1* transcriptome mRNA in fetal marmoset liver tissue. The uncut PCR-products were separated from the digested material by gel purification, and then cloned and sequenced. We sequenced 16 clones from each of the KpnI and BstI treated reactions, and in both cases, over 90% of the clones derived were the expected Cal_*ADH1.2* and Cal_*ADH1.4*; the remaining 10% were either Cal_*ADH1.1* or Cal_*ADH1.3* that presumably arose from incomplete digest.

#### ADH1 protein expression and characterization

To obtain evidence that all marmoset *ADH1* paralogous genes found via database mining encoded active proteins, synthetic genes based on the sequence obtained from the genome sequence project were made in the pET21 vector (BioBasic, Canada) and expressed in *E*. *coli* TUNER cell line. The heterologously expressed proteins were isolated following the procedures of Niederhut [40] and characterized using ethanol as a substrate (as in [41]). We were unable to get reasonable yield and purity for Cal_*ADH1.1* using this procedure, and instead subcloned it into pET41 and purified it over a nickel column using the accompanying C-terminal His-tag. A lemur *ADH1* paralog (clone 4B from brown lemur) was also isolated from cDNA clones and expressed following the same procedure. All of the proteins that were examined were found to be active at levels characteristic of other class I ADH enzymes (data not shown).

#### Phylogenetic analysis

Phylogenetic trees for exonic data were estimated using a codon model implemented with MrBayes [42] (using these parameters: nucmodel = codon nst = 6 rates = invgamma Ngammacat = 8 omegavar = Ny98; mcmc temp = 0.05 ngen = 300000, samplefreq = 100 printfreq = 100 nruns = 2 nchains = 4 burnin = 750). Phylogenetic trees for intronic data were also estimated with MrBayes (using these parameters: nucmodel = 4by4 nst = 6 rates = invgamma Ngammacat = 8 omegavar = equal; mcmc temp = 0.05 ngen = 300000 samplefreq = 100 printfreq = 100 nruns = 2 nchains = 4 burnin = 750). Neighbor-Joining trees [43], used for the exonic data partitioned into synonymous and nonsynonymous sites, were constructed using MEGAv4.0 [44], with evolutionary distances computed using the maximum composite likelihood model [45], using transition and transversion substitutions, a homogeneous pattern among lineages, and uniform rates among sites.

The extent of conservation at the third position of two fold redundant codons in exonic regions was measured using the procedure described by Li *et al.* [46]. These sites were then scored as unchanged or changed, with the fraction unchanged (*f*_2_) used to estimate the TREx distances and the dates of pairwise divergence using a rate constant of 3.1×10^−9^ silent substitutions/site/year [46], and an equilibrium silent nucleotide site bias of 0.52/0.48 (AT/GC).

For the eight introns in the *ADH1* genes, multiple sequence alignment (MSAs) were initially produced for each intron with Clustal X, and then manually optimized. These were then concatenated to form a combined *ADH1* intronic MSA.

Pairwise distance estimates (base substitutions per site) between homologous intronic regions were calculated using the Maximum Composite Likelihood method implemented by MEGAv4.0 [44], [45]. The calculation assumed a homogeneous pattern of transitions and transversions among lineages, assumed uniform substitution among sites, and ignored gapped sites shared by the pair.

Pairwise distance estimates for *ADH1* introns were calculated for all paralog and ortholog pairs in the *ADH1* intronic dataset, which included human *ADH1A*-1*C*, Cal_*ADH1.1*-*1.4*, and Mac_*ADH1.0*-*1.5* (where the prefix “Mac” refers to *Macaca*). Orthologs were assigned to the interspecies pairs having the smallest pairwise distance. The difference between the smallest interspecies pairwise distance and the next smallest pairwise distances was generally large, making the assignment of ortholog and paralog unambiguous. There were no discrepancies in ortholog assignment (i.e. conflicting pairs), and the ortholog assignment corroborated the intronic phylogeny. The Mac_*ADH1.0* pseudogene was included because phylogenetic analysis suggested it is the ortholog of Cal_*ADH1.1* (see below). Mac_*ADH1.2* was removed from pairwise distance analysis because initial analysis indicated it is a chimera of Mac_*ADH1.1* and *1.3*; further analysis demonstrated it was a recent duplicate of Mac_*ADH1.3* that later suffered gene conversion with Mac_*ADH1.1* (see below).

### Estimating *ADH1* Paralog Duplication Dates Using Non-*ADH1* Introns as a Reference

*ADH1* paralog duplication dates were estimated by comparing the pairwise distances among their intronic regions to the pairwise distances among orthologs from a variety of other genes and from a variety of species whose divergence spans our time period of interest. This work began with a set of sequences from 11 intronic regions from a variety of lemurs (strepsirhines) compiled by Horvath, *et al.* [47]. The Horvath intron sequences were used to BLAST the primate NCBI database to get orthologous intron sequences from several haplorhines. This “non-ADH” intronic data set was augmented with intronic regions from *ADH4*, which were retrieved from NCBI databases using human *ADH4* as a query.

To test the value of intronic sequences as a clock, these intronic regions were examined for indications that they many contain functional RNA motifs that might be subject to selection pressure. Each intronic region was examined for regions of unusually high conservation among mammals using the ‘mammal conservation plot’ of the USCS genome browser. This identified long conserved noncoding sequences (LCNS, also called ‘Ultra-Conserved Regions’, or UCR) encompassing the majority of the ERC2, LUC7, TTR, LRPPRC-Pair A and LRPPRC-Pair B intron in the Horvath *et al.* [47] data set. Because of their high level of sequence conservation, these intronic regions with LCNS were not included in our molecular clock analysis. None of *ADH1* or *ADH4* introns appeared to contain a LCNS.

Mutation rates are known to be higher in GC-rich regions of the genome, and in segments that lie within 15 Mbp of telomeres [48]. Therefore, introns near the telomeres or with high GC-ratios were removed from our intronic data set. This removed vWF (47.% GC, and 6.2 Mbp from the telomere), SREBF2 (49.5% GC, and 9.0 Mbp from the telomere), and NRAMP/SLC11a1 (47.1% GC, but not within 15 Mbp of the telomere). The *ADH1* loci are not near the telomere, and have normal GC-ratios (38.5%).

Finally, the remaining introns were screened for potential function by using the UCSC genome browser to determine whether they appeared in mRNA databases. None were found in the non-ADH introns, as well as in *ADH4* (introns 6, 7, and 8 being available from lemurs), *ADH1*A and *ADH1*C introns. However, *ADH1*B introns 1–5 were found in the UCSC human mRNA database. The pairwise distances for the concatenated introns 1–8 did not differ significantly from those calculated from introns 6–8 alone, however. The fact that *ADH1*B introns 1–5 have not diverged more slowly than *ADH1*A, *ADH1*C or *ADH1*B introns 6–8 (none of which appear in an mRNA database) suggests that presence of *ADH1*B introns 1–5 in the mRNA database does *not* imply functional constraints.

### Detecting Candidate Regions for Gene Conversion

By transferring sequence information from one gene to another, gene conversion produces regions of higher sequence identity between the two paralogs than found in non-converted regions. Gene conversion may be particularly noticeable for neutral sites, which cannot undergo function-driven adaptive convergence. To identify regions within paralogous sequences that suggest gene conversion, pairwise similarity plots with a sliding window (generally of 150 nucleotides for exonic data and 250-nt for intronic data) were constructed using “Similarity Calculator” (available at www.ffame.org.). Identities were given a score of *1.0*; mismatches were scored as 0. Sites containing a gap in the pairwise comparison were skipped. If gapped sites accounted for greater than 20% of the sites in the window, no score was reported for that window.

Several additional methods were used to detect potential gene conversion events: GENECONV [49], [50], BOOTSCAN [51], and RDP [52] were implemented for all intraspecies pairwise comparisons using the manual options in the software package RDP4.13. Bootscanning was executed using the “Jukes and Cantor 1969 model”, with window size = 200 and step size = 20 for intronic comparisons. Exonic sequences are much shorter, and thus were examined with Bootscanning using both a window size = 200 and 150, each with a step size of 2. RDP was executed using a window = 30 for intronic comparisons, and both a window = 10 and 15 for exonic regions. Bootscanning and RDP analysis conducted with and without the use of phylogenetic evidence for gene conversion yielded similar results. GENECONV was executed using all sites within both intronic and exonic regions and the following parameters: G-scale = 1, minimum aligned fragment length = 1 nucleotide; minimum polymorphisms in fragments = 2; minimum pairwise fragment score = 2; and maximum number of overlapping fragments = 1. Potential gene conversions were reported when P-values were <0.05, unless noted otherwise.

The command line version of GENECONV_1.81 was also used to analyze the ADHs from our “focal species” partitioned into three groups by genus: *Homo, Callithrix* or *Macaca*. Because we are concerned with gene conversion events between paralogs within each of the focal species, this approach increases statistical power by including sequence diversity in all sequences while implementing the Bonferroni multiple comparison correction [53]–[54] without including possibly spurious between-species comparisons. Intronic sequences were analyzed using the same parameters described above; exonic regions were analyzed using these parameters and a range of G-scales from zero to 3. The exonic regions of the *ADH1* paralogs from each focal species were analyzed separately, but included mouse (*Mus musculus*), dog (*Canis familiaris*), and treeshrew (*Tupaia belangeri*) ADH1 exonic sequences. All sequences were grouped by species. Intronic sequences are too divergent between primates and non-primate outgroups, and so GENECONV analysis was conducted with only the twelve ADH1 paralogs from marmoset, macaque and human. In this case, analysis was conducted for each species individually, and for all species simultaneously (but with paralogs grouped by species). The results of the RDP and command line implementations of GENECONV were generally similar, and where different, gene conversions detected by either method were included in our summary of gene conversion events.

## Results

### New Paralogs

#### Marmoset and macaque each contain four ADH1 paralogs

Mining of the human and chimpanzee public genome databases re-discovered three *ADH1* paralogs (*ADH1*A, *ADH1*B, *ADH1*C), the three that were already known in these two catarrhines. Further, database mining discovered a fourth *ADH1* paralog in macaque (*Macaca mulatta*), a more distantly related catarrhine. All four macaque *ADH1* paralogs were located between the macaque *ADH5* and *ADH4*, as in human and chimpanzee. Because it was not immediately clear which macaque genes were orthologs to human *ADH1A*, *ADH1B* and *ADH1C*, we named the macaque *ADH1* paralogs Mac_*ADH1.1*, Mac_*ADH1.2*, Mac_*ADH1.3* and Mac_*ADH1.4*, in order as they appeared in the chromosome starting from *ADH5* and ending at *ADH4* (Figure S1).

Further, a fifth duplicate, named Mac_*ADH1.0*, was found in the macaque genome between Mac_*ADH5* and Mac_*ADH1.1*. We suspect that this is a pseudogene for several reasons. First, Mac_*ADH1.0* appeared to contain no exon 1, although its short length (just 18 nucleotides) makes exon 1 difficult to find. Second, no in-frame start codon was found near the beginning of exon 2, necessary if exon 1 were missing. Third, exon 2 of Mac_*ADH1.0* contained a 21-nucleotide deletion that affects amino acid sites 24–32. Fourth, frameshifts deletions were found at three different locations; while sequencing error in draft genomes such as the macaque could easily account for a frameshift, three such errors are unlikely in a gene of 1125 nucleotides. Fifth, no exon 9 was found, yet an in-frame stop codon was found immediately after the end of exon 8 (an in-frame stop codon is also present at the beginning of intron 8 of other *ADH1* genes). Making this pseudogene assignment fall short of certainty, no stop codons were found within the exons that were discovered (suggesting Mac*1.0* became a pseudogene relatively recently).

The genome from *Callithrix jaccus* (marmoset, a New World primate, a platyrrhine) had been incompletely assembled when this mining was done. Nevertheless, mining indicated that marmoset also had four (apparently whole) *ADH1* paralogs located between its ADH5 and *ADH4* genes. The marmoset *ADH1* paralogs were also named sequentially in their chromosome as Cal_*ADH1.1*, Cal_*ADH1.2*, Cal_*ADH1.3*, and Cal_*ADH1.4*.

#### Expression of marmoset ADH1 paralogs suggests that they are all functioning genes

As some in the literature had assumed that the number of paralogs in *all* primates was the same as the three in humans and chimp [31], [32], finding four *ADH1* paralogs in the New World marmoset and (especially) the Old World macaque was surprising. Two approaches were therefore used to determine if the additional, apparently “whole”, *ADH1* paralogs in macaque and marmoset are expressed as mRNA.

First, BLAT [55] was used to mine the macaque EST database [http://www.ncbi.nlm.nih.gov/sra] and the marmoset “sequence read archive” (SRA) database [http://www.ncbi.nlm.nih.gov/nucest]. This mining found evidence for transcripts from three macaque paralogs (Mac_*ADH1.2, 1.3,* and *1.4*) and two of the four marmoset genes (Cal_*ADH1.1* and *1.3*, but not Cal_*ADH1.2* and *1.4*, Table S4).

The failure to find transcripts for genes in an EST database does not rule out their transcription, however. For example, human *ADH1*A is expressed predominantly in the fetus, and might be missed in a database that contains few fetus ESTs. The marmoset SRA database does not contain fetal ESTs, and the macaque database appeared to contain few (if any) fetal ESTs.

Accordingly, we applied RT-PCR to amplify and clone *ADH1* transcripts isolated from fetal marmoset liver and compared the results to transcripts detected by amplifying and cloning mRNA from adult marmoset liver. Cal_*ADH1.1* and Cal_*ADH1.3* genes were recovered as cDNA clones from both adult and fetal liver mRNA, consistent with results obtained from database mining. Cal_*ADH1.2* and Cal_*ADH1.4* genes were recovered from fetal liver mRNA. This suggested that all four marmoset genes are functional, with the differential expression of these in different tissues suggesting that the different paralogs have different functions.

As a final indication that all four marmoset *ADH1* paralogs were functional, we expressed each of these eight proteins in *E. coli* (Material and Methods). Kinetics on the purified proteins showed that all are active as enzymes, with kinetics for the oxidation of ethanol similar to those of human *ADH1* paralogs (data not shown).

#### Lemurs (strepsirhines) contain multiple ADH1 paralogs

We extended mining efforts to databases emerging from sequencing projects underway for two lemurs (strepsirhines), *Microcebus murinus* (mouse lemur) and *Otolemur garnetti* (bushbaby). At the time when our mining was done, coverage within the *ADH1* regions of these two strepsirhines was insufficient to permit assembly of contigs covering substantial lengths of the *ADH1* locus. Nonetheless, we found evidence for two *ADH1* paralogs in the bushbaby genome, and for three *ADH1* paralogs in the mouse lemur genome, based on intronic regions.

To supplement mining, we also sequenced cDNAs obtained from adult liver mRNA from the ring-tailed lemur (*Lemur catta*) and sifaka (*Propithecus coquereli*). These yielded four and three *ADH1* paralogs respectively. This is discussed in greater detail below.

### Relative Order of Duplications and Speciations

#### Exon sequences favor independent *ADH1* duplications in platyrrhine and catarrhine lineages

Assuming gene duplications are rare, the presence of four *ADH1* paralogs in both marmoset and macaque suggests that four *ADH1* paralogs were also present in the their common haplorrhine ancestor. As an alternative model, given that only three *ADH1* paralogs exist in human and chimp (representing hominoids), only three *ADH1* paralogs may have been present in the haplorrhine ancestor, with an additional paralog generated independently and separately in each of the lineages leading to marmoset and macaque.

**Figure 2:**
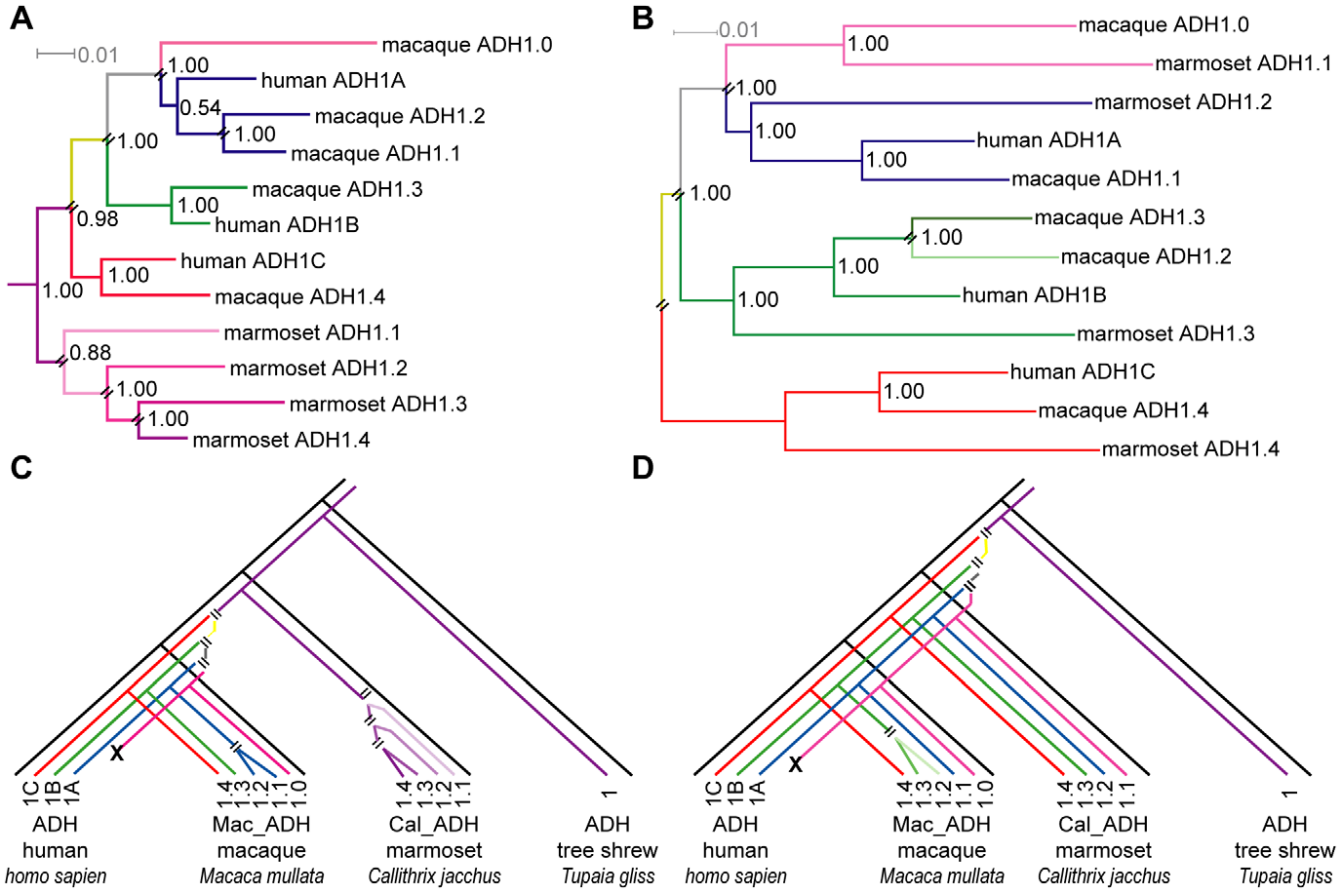
Phylogeny of primate *ADH1* paralogs. Phylogeny of primate *ADH1* paralogs inferred from (A) exonic sequence data (“exonic tree”) and (B) intronic data (“intronic tree”). Parallel black lines indicate bifurcations associated with gene duplications without speciation. *ADH1* genes from New World monkeys (represented by marmoset) form a separate clade from the hominid/OWM genes in the exonic tree (A), while they interleave with hominid/OWM genes in the intronic tree (B). The lower panels, (C) and (D), redraw the gene tree from (A) and (B) in a species tree format, highlighting where each gene duplication occurs relative to the divergence of each primate lineage. The exonic tree is rooted using multiple non-primate *ADH1* genes (see Figure S2). The intronic tree is unrooted (due to ambiguity, see text). The names of *ADH1* paralogs have been shortened (e.g. the marmoset (*Callthrix jacchus*) ADH1 paralog “Cal_*ADH1.1”* is simply referred to as “marmoset ADH1.1”). Numbers at nodes refer to the Bayesian posterior probability values.

However, phylogenetic analysis of the exonic sequences suggests neither (Figure 2A). This “exonic phylogeny” places only one *ADH1* gene in the last common ancestor of all anthropoids, implying that the mulitiple *ADH1* paralogs in modern catarrhines (macaque and human) and platyrrhines arose by independent gene duplications in each lineage after the catarrhine and platyrrhine primates diverged.

When lemur paralogs were included within the exonic sequence analysis, they formed a separate clade as an outgroup to both the catarrhine and platyrrhine clades (Figure S2). This phylogeny also indicated the duplications yielding multiple ADH paralogs in sifaka and ring-tailed lemur occurred independently in each lineage. This implies multiple, independent *ADH1* duplications in each of the strepsirhines, catarrhines and platyrrhines lineages.

#### Support for the exonic model is weak, however

Although 1125 sites were present in the haplorhine exon dataset, only 112 of these were informative. Of these, just 24 support the exonic phylogeny, and only 16 of these hold synonymous substitutions. Another 88 sites are homoplasic, having parallel or reverse nucleotide substitutions across the tree; half of these homoplasies make synonymous changes and therefore have no functional consequence on the encoding protein (Figure S3). This number is unexpectedly large, and indicates that stochastic models for nucleotide replacement describe only imperfectly the natural history of these regions and therefore calls into question phylogenetic conclusions based on simple bioinformatics modeling.

Further, the number of supporting sites relative to the number of homoplasic sites is not much different when branches are swapped to give different tree topologies. For example, when the exon sequences are modeled according to a tree that interleaves marmoset *ADH1* paralogs with human and macaque *ADH1* paralogs (as in Figure 2B), twelve sites support this alternate phylogeny, with nine of these being synonymous. Another 100 sites are homoplasic (Figure S3). The alternative trees (Figure 2A and 2B) share 82 homoplasic sites.

Thus, the tree in Figure 2A is favored by both supporting sites (24 versus 12 for the tree in Figure 2B) and homoplasic sites (88 versus 100 for the tree in Figure 2B). However, the differences are not large, especially considering the evidence for adaptive change in this family coming from the location of many amino acid replacements in the active sites of these proteins and the large number of conserved paralogs. Homoplasies can result from convergent evolution of function. This, however, cannot explain the abundance of homoplasies at synonymous sites.

#### Analysis of introns favors a haplorhine ancestor already having multiple *ADH1* paralogs

The longer intron sequences available for human, macaque and marmoset paralogs provide more informative characters than their corresponding exon sequences, found among some 12,500 sites when the eight introns are concatenated. Intron sites also presumably diverge with little selective pressure, making them more likely to behave as expected under canonical stochastic models. Therefore, we repeated the phylogenetic analysis of the *ADH1* genes using intron sequences, including the sequence of the macaque pseudogene. We asked whether the intronic sequence data confirmed the exonic tree in Figure 2A.

They did not. Instead, the topology of the best scoring tree obtained from intron sequence data had three *ADH1* clades that each contained a paralog from each of the marmoset, the macaque, and the human (the “intronic phylogeny”, Figure 2B). This intronic phylogeny requires that the haplorhine ancestor of Old and New World primates contained *four* paralogs (with the subsequent loss of the human ortholog in the Mac_*ADH1.0*/Cal_*ADH1.1* clade), not the single exemplar implied by exon sequences. Further, it implied that the duplications creating those four *ADH1* paralogs occurred *before* the catarrhines and platyrrhines diverged, not afterwards, as in the exonic phylogeny.

When intronic data available from lemurs was included (reducing the dataset to only parts of introns 1, 2, 3, 6 and 7), the lemur paralogs formed a monophyletic clade sister to the haplorhine paralogs, leaving the phylogeny of haplorhine introns paralogs unchanged (Figure S4). In other words, the phylogeny inferred using the limited lemur intronic data suggests a single ADH paralog in the urprimate ancestor, with independent duplications in both the strepsirhine and haplorhine lineages. Further, while the amount of lemur intronic sequence data was too little to support strong phylogenetic conclusions, they nonetheless suggested that the duplications yielding multiple *ADH1* paralogs in bushbaby and mouse lemur also occurred independently in each lineage.

#### Molecular clock-based comparisons of intronic regions suggest *ADH1* duplications predate the platyrrhines-catarrhines split

Clearly, a natural history that has the *ADH1* paralogs duplicating and diverging *before* the Old and New Worlds split embodies different functional implications than a natural history that has *ADH1* paralogs duplicating and diverging *after* the Old and New Worlds split. Resolving this discrepancy is vital to correctly interpreting the evolutionary history of this gene family and its functional implications. A detailed examination of the evolutionary history of highly duplicitous gene families such as the ADH gene family may also shed light on processes important to the evolution of function in duplicated genes.

If the intronic tree (Figure 2B) were correct and the formation of the *ADH1* paralogs predates the platyrrhine-cattarhine split, then for each human gene, the pairwise distances between one human-marmoset pair should be lower than all of the other human-marmoset pairs, and lower than all human-human paralog comparisons. Likewise, the marmoset intron that pairs best with each human gene should *not* be the marmoset gene that pairs best with any of the *other* human gene. Last, the distances between the so-assigned pairs of orthologous gene should be roughly the same.

As Table 1 shows, these conditions are met for intronic regions. For example, the concatenated intronic regions of human *ADH1*A pairs best with marmoset ADH_*1.2* (distance 0.127); the corresponding distances to marmoset ADH_*1.1*, *1.3* and *1.4* are 0.149, 0.146, and 0.165. Likewise, the human *ADH1*B paralog pairs best with marmoset *1.3* (distance 0.124); the distances to the other marmoset paralogs ADH_*1.1*, *1.2*, and *1.4* are 0.169, 0.154, and 0.161. The human *ADH1*C paralog pairs best with marmoset ADH_*1.4* (distance 0.120); the distances to the other marmoset paralogs ADH_*1.1*, *1.2*, and *1.3* are 0.171, 0.170, and 0.169. The ortholog pairwise distances are all similar (0.127, 0.124, 0.120), suggesting that the series of duplications occurred in relatively rapid succession.

**Table 1:** Pairwise distance estimates of *ADH1* intronic regions.

paralog	1: Human*ADH1A*	2: Human*ADH1B*	3: Human*ADH1C*	4: Mac*ADH1.0*	5: Mac*ADH1.1*	6: Mac*ADH1.2*	7: Mac*ADH1.3*	8: Mac*ADH1.4*	9: Cal*ADH1.1*	10: Cal*ADH1.2*	11: CalADH*1.3*	12: Cal*ADH1.4*
1: Human *ADH1A*		[0.003]	[0.004]	[0.005]	[0.002]	[0.004]	[0.003]	[0.004]	[0.004]	[0.004]	[0.004]	[0.004]
2: Human *ADH1B*	0.123		[0.004]	[0.005]	[0.004]	[0.003]	[0.003]	[0.005]	[0.004]	[0.004]	[0.004]	[0.003]
3: Human *ADH1C*	0.143	0.139		[0.004]	[0.004]	[0.004]	[0.004]	[0.003]	[0.004]	[0.004]	[0.004]	[0.004]
4: Mac *ADH1.0*	0.134	0.146	0.155		[0.005]	[0.005]	[0.006]	[0.005]	[0.004]	[0.004]	[0.006]	[0.005]
5: Mac *ADH1.1*	0.059	0.134	0.153	0.143		[0.003]	[0.003]	[0.004]	[0.004]	[0.004]	[0.005]	[0.004]
6: Mac *ADH1.2*												
7: Mac *ADH1.3*	0.139	0.072	0.153	0.163	0.151			[0.004]	[0.004]	[0.004]	[0.004]	[0.004]
8: Mac *ADH1.4*	0.152	0.146	0.065	0.164	0.16	0.158	0.158		[0.005]	[0.005]	[0.005]	[0.004]
9: Cal *ADH1.1*	0.149	0.169	0.171	0.129	0.158	0.178	0.187	0.179		[0.004]	[0.005]	[0.004]
10: Cal *ADH1.2*	0.127	0.154	0.170	0.164	0.132		0.170	0.179	0.158		[0.004]	[0.005]
11: Cal ADH*1.3*	0.146	0.124	0.169	0.175	0.157		0.141	0.176	0.183	0.152		[0.004]
12: Cal *ADH1.4*	0.165	0.161	0.12	0.184	0.173	0.175	0.177	0.127	0.185	0.183	0.172	

Pairwise distance among the *ADH1* paralogs for the concatenated intronic dataset were calculated using the Maximum Composite Likelihood method implemented by MEGAv4.0. Pairwise distances are shown in the lower left of the table, with variance estimates in the upper right of table.

These comparisons, of course, are merely particulars amid the entire dataset that is used to construct the intronic phylogeny in the first place. While the comparisons are consistent with the intronic tree (Figure 2B), the particulars cannot be said to provide entirely independent support for that tree. Therefore, we examined several other approaches to see if they might support one or the other phylogeny.

In principle, molecular clocks that estimate the dates of the paralog duplication might be compared with similar estimates of the platyrrhine-catarrhine divergence date to offer an independent test. The comparison might help distinguish the two alternative phylogenies. Intronic sequences offer more data than exon sequences, and therefore an opportunity for an improved estimate of the relative date of the *ADH1* paralog duplications. For example, the distances between each marmoset *ADH1* paralog and its catarrahine orthologs is less than the average distance among intra-species paralogs (Table 2), suggesting paralog duplication predates the ortholog speciation. Extending this rationale, we compared pairwise distances between introns from *ADH1* paralogs to distances among introns from orthologous genes of strepsirhines, catarrhines and platryrrhines that were *not* alcohol dehydrogenases.

**Table 2:** Average of pairwise distances for ADH1 intronic regions (shown in Table 1) among paralogs and between orthologs.

	Average of pairwise distances between according to group		
	Paralogs	Ortholog: human+macaque vs marmoset	Ortholog: human vs macaque
Average (all)	0.158+/−0.017	0.129+/−0.007	0.065+/−0.007
Human paralogs	0.135+/−0.011		
Macaque paralogs	0.157+/−0.007		
Marmoset paralogs	0.172+/−0.014		

The rate of intron divergence is *not* clock-like in some regions. For example, some introns contain “long, conserved non-coding sequences” (LCNS; also known as “ultraconserved regions”, UCR) [56]. Further, genomic regions within 15 Mbp of the telomere, or with high GC-content, are known to diverge more rapidly [48] The ADH loci (including the neighboring ADH5 and *ADH4* genes) contain none of these special features. Therefore, we built a dataset of *non-ADH1* reference genes, starting with the dataset compiled by Horvath *et al.* [47] and removing introns that contained LCNS, were within 15 Mbp of the telomere, or had high GC-content. We added to this dataset introns 6, 7 and 8 from *ADH4* (the gene immediately adjacent to the *ADH1* paralogs).

We then calculated distances between pairs of orthologous introns in our dataset (including both *ADH1* and *non*-*ADH1* genes) and between pairs of introns from *ADH1* paralogs (from our three focal anthropoids, human, macaque and marmoset).

**Figure 3:**
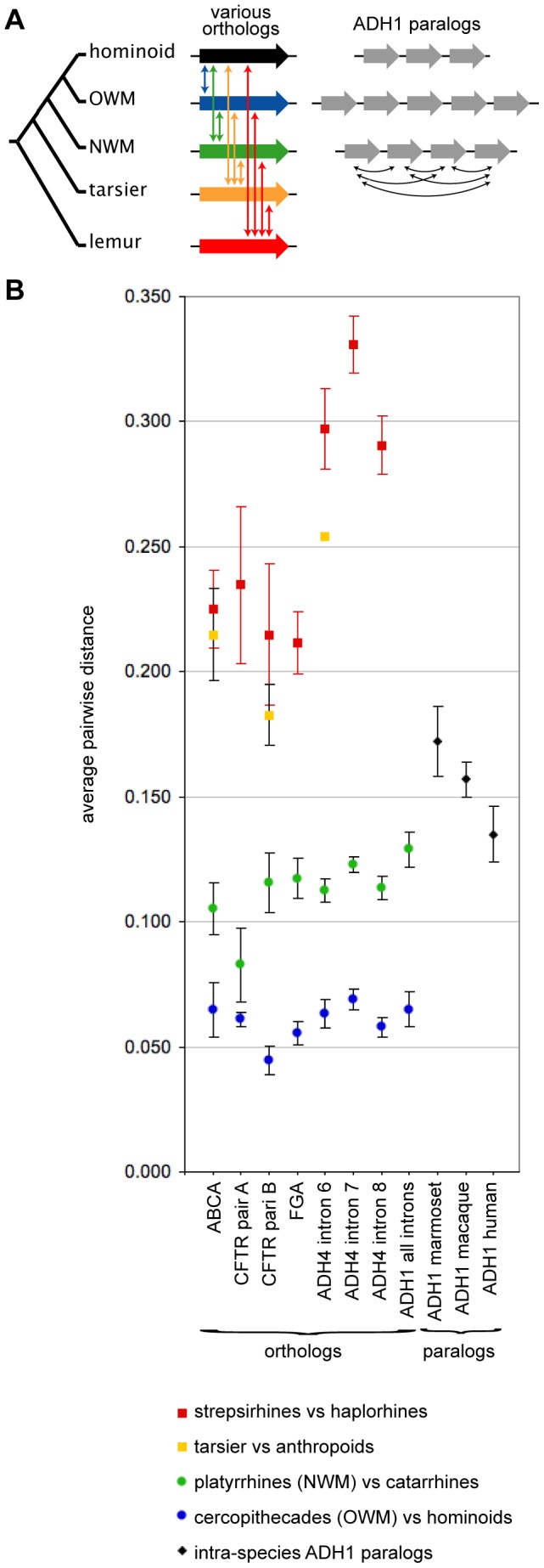
Estimate of the ADH1 paralog duplications relative to the time of the major primate speciation events. The average pairwise distances separating the introns of the *ADH1* paralogs were compared with the average pairwise distances separating a set of introns in paired taxa. (A) This schematic illustrates the various ortholog comparisons used to estimate the relative age among the ADH1 paralogs. (B) This plot summaries the data in Table 2 and 3. The distances among the *ADH1* paralogs in marmoset, macaque and human (black diamonds) are somewhat larger than those separating catarrhine and platyrrhine orthologs (green circles), implying that these *ADH1* paralogs diverged (duplicated) before the catarrhine-platyrrhine split. Conversely, distances separating the *ADH1* paralogs in marmoset, macaque and human are somewhat smaller than those separating orthologous introns among strepsirhine and haplorhine (red squares), implying that these *ADH1* paralogs diverged after the split between strepsirhine and haplorhine.

Pairwise comparison of orthologous introns from a platyrrhines and catarrhines found distances ranging from 0.083 to 0.129 nucleotide substitutions per site (Table 3). This is less than the average pairwise distance among *ADH1* paralogs: 0.135 in human, 0.157 in macaques, and 0.174 in marmosets (Table 2 and Figure 3). This suggests the *ADH1* paralog duplication predates the platyrrhines-catarrhines split, consistent with the intronic tree in Figure 2B.

**Table 3:** Pairwise distance estimates for orthologous intronic regions in a dataset composed of non-*ADH1* genes.

				# of sequences within each clade				Average of pairwise distances among pairs from clades *X* vs *Y*							
gene from whichintronic sequencederives	position inhumanchromosome	%GC insurrounding1 Mbp	length	strepsirhine	NWM	OWM	Hominoid	strepsirhinevs tarsier	strepsirhinevs NWM	strepsirhinevs OWM	strepsirhinevs hominoid	strepsirhinevs haplorhine	tarsier vsanthropoid	NWM vscatarrhines	OWM vshominoid
ABCA	chr9∶107,676,904-107,677,398	38.1%	495	22	1	1	4	0.232+/−0.020	0.236+/−0.014	0.218+/−0.011	0.222+/−0.014	0.225+/−0.016	0.215+/−0.018	0.105+/−0.010	0.065+/−0.011
CFTR_A	chr7∶117,304,915-117,305,512	37.2%	598	21	6	4	5		0.234+/−0.031	0.245+/−0.030	0.227+/−0.031	0.235+/−0.031		0.083+/−0.015	0.061+/−0.003
CFTR_B	chr7∶117,305,623-117,306,129	37.2%	511	14	1	3	3	0.186+/−0.024	0.226+/−0.021	0.232+/−0.023	0.208+/−0.024	0.215+/−0.028	0.183+/−0.012	0.116+/−0.012	0.045+/−0.006
FGA	chr4∶155,508,073-155,508,661	38.2%	589	14	1	1	4		0.228+/−0.008	0.222+/−0.008	0.205+/−0.008	0.212+/−0.013		0.117+/−0.008	0.056+/−0.004
*ADH4* intron6	chr4∶100,340,365-100,341,689	38.5%	1392	1	1	1	4	0.286	0.325	0.297	0.293+/−0.014	0.297+/−0.016	0.254	0.113+/−0.005	0.064+/−0.006
*ADH4* intron7	chr4∶100,337,348-100,338,924	38.5%	1598	1	1	1	3		0.344	0.342	0.323+/−0.002	0.331+/−0.011		0.123+/−0.003	0.069+/−0.004
*ADH4* intron8	chr4∶100,334-331-100,335,632	38.5%	1409	1	1	1	4		0.309	0.300	0.284+/−0.004	0.291+/−0.012		0.114+/−0.005	0.058+/−0.004

The distances between anthropoid *ADH1* paralogs, however, are less than distances between orthologs from strepsirhine-haplorhine comparisons, indicating the *ADH1* paralogs found in the anthropoids duplicated after the strepsirhine-haplorhine split. This too is consistent with the intronic tree that includes strepsirhine introns (Figure S4).

A similar approach was applied to synonymous (2-fold degenerate) sites within the exonic dataset Text S1 and Table S5). The duplication dates of the *ADH1* paralogs was approximately the same as *ADH1* orthologs, but molecular clock estimates of *ADH1* ortholog duplication dates were consistently underestimated from *ADH1* exonic data (relative to the *ADH4* gene used to calibrate the molecular clock). The substantial variance in exonic molecular clock dating meant the test was indecisive (see supporting information).

### Role of Gene Conversion

#### Homoplasies at synonymous and nonsynonymous sites suggest gene conversion rather than convergent functional evolution among the *ADH1* paralogs

The intronic phylogeny is supported by molecular clock estimates indicating the duplications of *ADH1* predate the catarrhine-platyrrhine split, while the exonic phylogeny is weakened by the abundance of homoplasies. Gene conversion offers one explanation for unexpected features of molecular evolution that do not fit standard stochastic models. Given that there are less phylogenetically informative characters in the exonic region, gene conversion amongst marmoset *ADH1* genes (or among *ADH1* paralogs in the ancestor of all catarrhines) could explain why the exonic tree differs from the intronic tree, and would also account for the large amount of homoplasy at synonomous sites. When modeled using standard stochastic tools, gene conversion can generate sites that appear to be homoplasic. The homoplasies created by gene conversion should differ, however, from homoplasies caused by functional convergence.

In particular, homoplasies arising from functional convergence should lie primarily at non-synonymous sites in coding regions. In contrast, homoplasies from gene conversion should lie equally in synonymous and non-synonymous sites, with purifying selection possibly removing them from the latter. Further, gene conversion should give homoplasies clustered along the linear sequence. The distribution of homoplasies arising from functional convergence should be more complicated, reflecting the protein fold and other functional features.

Once stochastic models averaged over the entire lengths of individual genes are abandoned, statistical tools to distinguish different kinds of homoplasies become difficult to construct. This notwithstanding, with *ADH1* paralogs, homoplasies do not appear randomly distributed in the linear sequence (e.g. exon 6 has very few homoplasies, Figure S3). Further, nearly half of the homoplasies in the coding regions are found at synonymous sites, more than expected from functional convergence. Further, of the approximately 45 homoplasies affecting non-synonymous sites, many map onto residues *not* believed to be functionally critical from crystallographic and mutant data (Figure S5). These observations all are positive indicators of gene conversion, albeit weak ones.

To extract more information from patterns of conservation in synonymous and non-synonymous sites within exons, we separated non-synonymous (but informative) sites from the synonymous (but informative) sites in the focal *ADH1* genes. If the marmoset *ADH1* paralogs form a monophyletic group in the exonic tree because of gene conversion, then the tree made from synonymous sites *and* the tree made from non-synonymous sites should be congruent to the tree derived from the entire exonic dataset. Alternatively, if functional convergence accounts for the abundance of homoplasic sites, then only the tree made from the non-synonymous sites should be congruent to the tree from the entire exonic dataset; the tree made from synonymous sites should be congruent to the intronic tree.

Both trees turn out to be congruent to the exonic tree (Figure S6), at least regarding the clustering of the marmoset paralogs as a separate clade. This suggests that the differences between the exonic phylogeny and intronic phylogeny are *not* driven predominantly by adaptive evolution at non-synonymous sites. This also suggests that functional convergence cannot easily explain the abundance of homoplasic sites and the discrepancy between exonic and intronic phylogenies. Again, this indicates gene conversion, but only weakly.

#### Phylogenetic analysis of individual introns suggests discordant evolutionary histories

Gene conversion is expected to create “mosaic” patterns [57], with greater sequence similarity in a pairwise alignment between converted segments than non-converted segments. Because gene conversion results in regions of increased similarity, they are often detected by comparing the sequence similarity or phylogeny between sub-domains of the entire sequence. Regions that have suffered gene conversion have phylogenies that do not correspond to the phylogeny generated for the alignment overall, or have regions where pairwise similarities are higher than expected from the pairwise identities overall. We applied this approach by examining separately the phylogeny of each of the eight *ADH1* introns in the focal species in an effort to detect mosaic structure.

While the phylogenies of most individual introns were consistent with the phylogeny derived from the concatenated intronic dataset, some notable deviations were found (Figure S7). For example, intron 3 of Mac_*ADH1.2* grouped more closely to the paralogs in the Mac_*ADH1.1*/human *ADH1*A clade rather than those in the Mac_*ADH1.3*/human *ADH1*B clade. The other introns of Mac_*ADH1.2* grouped more closely the paralogs in the Mac_*ADH1.3*/human *ADH1*B clade. This is precisely the pattern expected from a gene conversion in which intron 3 of Mac_*ADH1.2* acquired sequence information from Mac_*ADH1.1* via gene conversion (or vice versa).

Discordance between the phylogeny derived from the combined dataset and the phylogeny of individual introns is also seen among marmoset paralogs (specifically regarding the placement of Cal_*ADH1.2* introns 6, 7 and 8). In the phylogeny of the concatenated intronic dataset (as well as intron 1 and 2 individually), Cal_*ADH1.2* is positioned at the base of a clade including human *ADH1*A and Mac_*ADH1.1*. The phylogeny changes for intron 3, with Cal_*ADH1.2* forming a clade together with Cal_*ADH1.1*, and then again for introns 5, 6, 7, and 8, with Cal_*ADH1.2* grouping closest to Cal_*ADH1.3*.

Gene conversion tracts reportedly range from as few as a dozen nucleotides long to as many as several thousand [58]. Given that most of the *ADH1* introns are >1500 bases long, a gene conversion must be fairly large in order to alter the phylogeny of that intron, suggesting that small gene conversions may have gone unnoticed when analyzing an intron as a whole unit.

**Figure 4:**
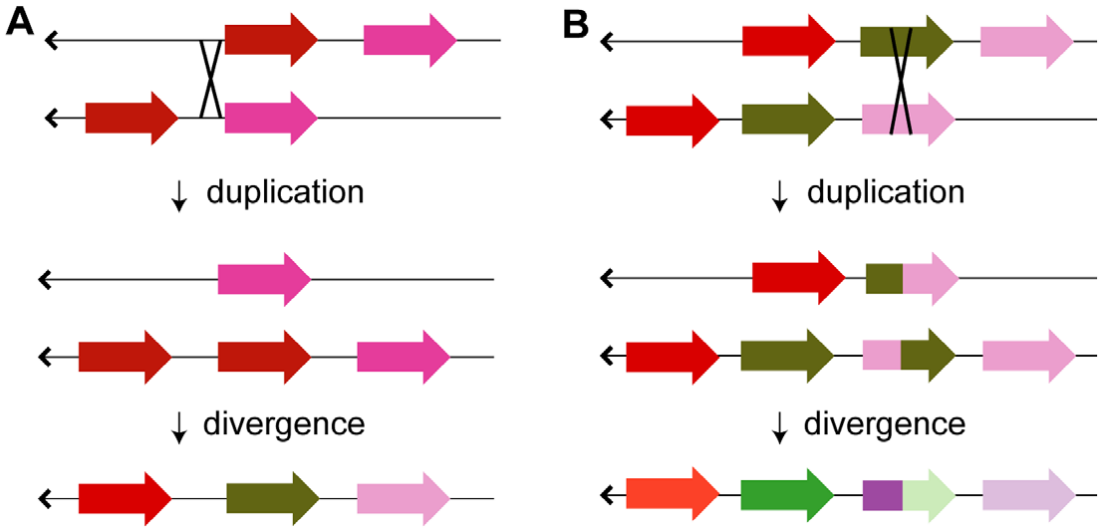
Gene duplication can generate “whole gene” and “chimeric gene” paralogs. (a) When unequal crossing-over (denoted with an “X”) occurs within the intergenic region between two paralogs, one chromosome gains an extra copy of a paralog, while the other chromosome loses one of the paralogs. This is followed by divergence of each paralog (only shown for the chromosome that gained a paralog and denoted as shift in color). A similar process can lead to the creation of the original paralog duplication, if, for example, transposons generate regions of sequence similarity on either side of a gene, thus enabling unequal crossing-over (not shown). (b) The same process can also lead to a chimeric gene duplicate if the crossing over occurs within the intragenic region (most likely within an intronic region).

Paralogs are generally assumed to derive from duplications of a single contiguous parent gene, yielding two nearly identical copies of a single parent gene. However, the observation that the phylogeny of large tracts of Cal_*ADH1.2* (introns 5–8) deviates from that of the combined dataset (and intron 1 and 2 individually) in a consistent manner encompassing nearly 6000 basepairs suggests either (a) repeated gene conversions, (b) a recombination event, or (c) a gene duplication from two parent paralogs (presumably Cal_*ADH1.1* and Cal_*ADH1.3*) yielding a chimeric paralog (see Figure 4).

The intronic phylogeny of the combined dataset and molecular clock estimates of *ADH1* paralog duplication both suggest four paralogs were present prior to the catarrhine-platyrrhine split, and that the pseudogene Mac_*ADH1.0* is the only catarrhine descendent of the clade including Cal_*ADH1.1* (this hypothesis is also supported by micro-indel evidence described later). Therefore, if Cal_*ADH1.2* is a chimeric duplicate, then we would also expect its macaque ortholog, Mac_*ADH1.1*, to be a chimeric duplicate as well. We examined this possibility by calculating pairwise distances among all paralogs for each intron individually. The two paralogs with the smallest pairwise distance are assumed to derive from the most recent gene duplication. If Cal_*ADH1.2*, and its macaque ortholog Mac_*ADH1.1*, derive from a chimeric duplication event, then the 5′ portion of both chimeric paralogs will be most similar to the parent paralog located towards their 3′-end, while the 3′ portion of both chimeric paralogs will be most similar to the other parent paralog located on their 5′ end. Table S6 shows this to be true for both Cal_*ADH1.2* and Mac_*ADH1.1*. The absence of the human ortholog of Cal_*ADH1.1*/Mac_*ADH1.0* prevents similar analysis for human *ADH1*A, but comparisons of human *ADH1*A to macaque paralogs suggests its duplication history parallels Cal_*ADH1.2* and Mac_*ADH1.1*.

#### Multiple approaches identify gene conversions within ADH1 paralogs

Several lines of evidence (discussed above) suggest both exonic and intronic regions have suffered gene conversion. Gene conversion within the *ADH1* paralogs has been proposed by Cheung, *et al.* [31], but a later investigation found no support for the gene conversion hypothesis and instead suggested the *ADH1* family was undergoing strong purifying selection [32]. We sought to re-examine the possibility of gene conversion with the benefit of a larger dataset, one that includes the complete genomic set of *ADH1* genes (in their entirety, with all exons and introns) and from a more comprehensive sample of primate species. We also sought to utilize multiple methods of detecting gene conversion within both intronic and exonic regions.

Discordant phylogenies among introns suggests gene conversion, but gene conversions need not conform to intron/exon boundaries, nor be large enough to alter the phylogeny of an entire intron. Therefore, the first approach to detect specific gene conversion events searched for mosaic structure by calculating pairwise similarity scores within a sliding window for all pairs of paralogs. These sliding window similarity scans were applied to both exonic and intronic sequence alignments. When a pairwise comparison yields a region with high similarity scores relative to the rest of the gene, or to other pairs in the same region, this region is considered a candidate for gene conversion between that pair. The hypothesis of gene conversion gains greater support when increased sequence similarity is found in synonymous sites [60].

**Figure 5:**
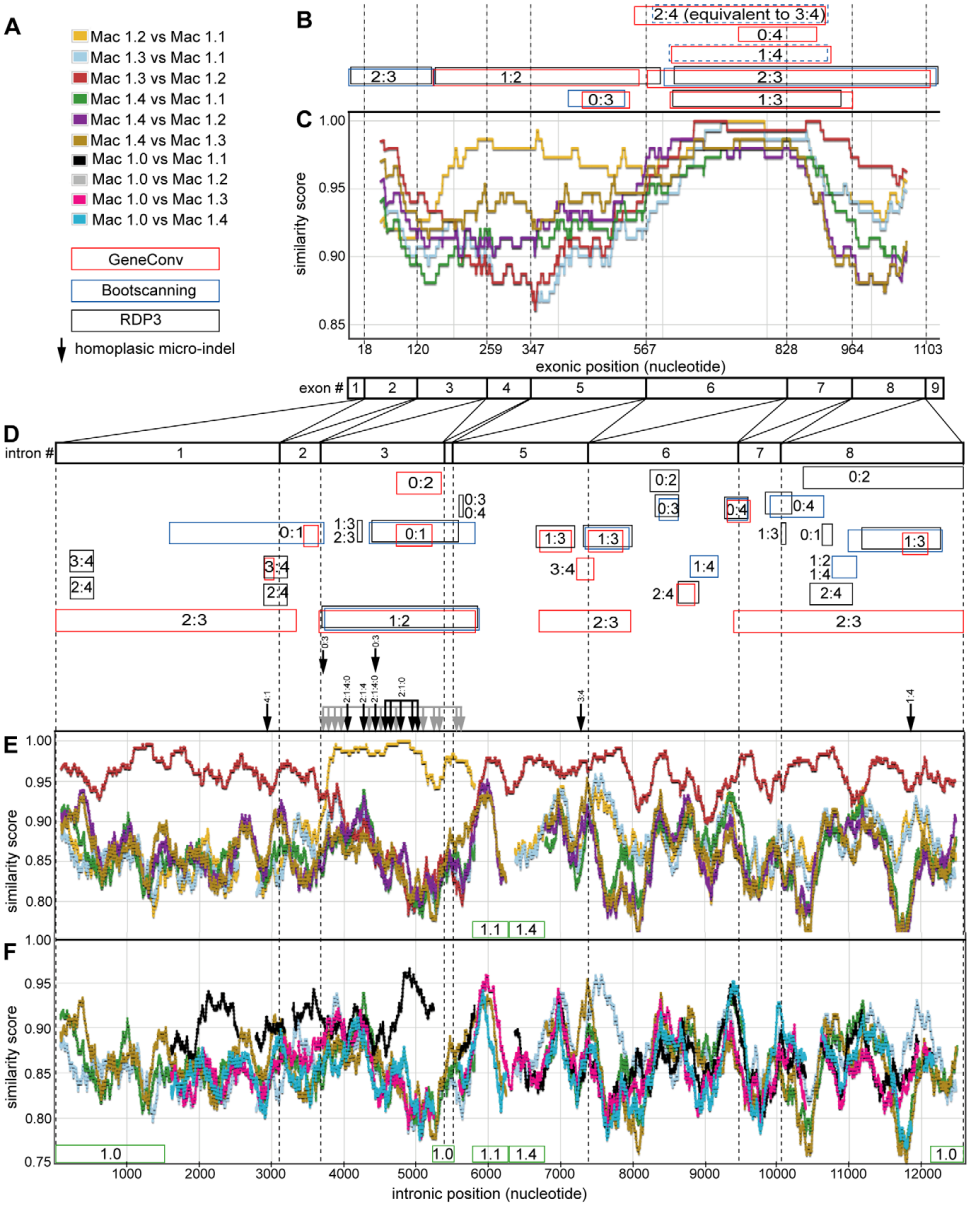
Summary of gene conversion analysis for macaque ADH1 paralogs. Exonic and intronic data sets were examined for indicators of gene conversion using similarity plots, homoplasic micro-indels, and various computational methods. (A) The figure legend displays the color schemes used in subsequent panels for illustrating pairwise similarity scores among paralogs, and the key used to summarize the results from various methods used to identify potential gene conversions. The names of *ADH1* paralogs have been shortened (e.g. the macaque (*Macaca mullata*) *ADH1* paralog “Mac_*ADH1.1*” is simply referred to as “Mac 1.1”). Pairwise similarity within a sliding window is plotted for various paralogs within (C) exonic regions (Mac_*ADH1.0* is not included), (E) intronic regions (without Mac_*ADH1.0*, the pseudogene), and (F) intronic regions including Mac_*ADH1.0*. The color of the line in the similarity plot corresponds to the identity of the paralog pair, as indicated in the figure legend (A). Similarity scores for exonic regions are calculated within a 150-nt sliding window, while that of intronic regions are calculated using a 250-nt sliding window. Colored boxes in (B) and (D) indicate putative gene conversion events identified by various computation methods. The color of the box corresponds to the computational method identifying each potential gene conversion, as indicated in the figure legend (A). Boxes with dashed borders indicate gene conversions that were not statistically significant at p-values <0.05, but were identified using p-values <0.10. The paralogs implicated in gene conversion are indicated within (or adjacent to) the colored box using the paralog suffix (e.g a gene conversion between Mac_*ADH1.1* and Mac_*ADH1.2* is indicated by “1∶2”). Homoplasic micro-indels in the intronic sequences are shown as vertical black arrows with the paralogs sharing these micro-indels indicated above each each arrow (the many homoplasic micro-indels shared by Mac_*ADH1.1* and Mac_*ADH1.2* are simply indicated with grey arrows). Boundaries between introns or exons are demarcated with dotted vertical lines. Green boxes below the similarity plots indicate large gaps in the alignment, with the affected paralog indicated within the box.

Similarity plots are often used to detect patterns of mosaic structure among genes, leading to the inference of a gene conversion (see [61]–[63]; and references in [64]); computational methods have since been developed that add statistical rigor to detecting gene conversions [64]. A second approach to detect gene conversion applied three of these computational methods: RDP, GENECONV, and Bootscanning. These are summarized in Figure 5, S8 and S9.

Evidence drawn from similarity plots and computational methods often lead to incompletely convincing conclusions about gene conversions. Similarity plots are not statistically rigorous, and because they rely on dramatic deviations from expectations to be persuasive, short, overlapping, or ancient gene conversions are difficult to substantiate (particularly within coding regions, where selection pressures confound any potential signal of gene conversion). Computation approaches are highly dependent on the model parameters, and different methods yield different results [64]. Thus, most gene conversion identified by similarity plots or computations methods are consider provisional “working hypotheses”.

Accordingly, a third approach was sought that might help distinguish between the alternative phylogenies in Figure 2 and help confirm gene conversion. Micro-indels, short insertion-deletion events that created gaps within intronic alignments, offered this. When indels of identical length occur at the same site in the alignment and are shared between two or more genes, these “shared” micro-indels are informative characters that support tree construction and can provide evidence for gene conversion (see examples in Figure S10).

Over 150 informative indels were found within the ca. 12,500-nt intronic alignment. These were encoded as binary data and analyzed using parsimony to compare the alternative phylogenies in Figure 2 (micro-indels encoded as binary data are shown in Figure S11). This micro-indel analysis supported the intronic phylogeny (Figure 2B), with 247 micro-indel events required for the 2B phylogeny compared to 369 steps required for the 2A phylogeny (Figure S12). As the intronic tree in Figure 2B was constructed with the gapped sequences removed when calculating distances, the micro-indel analysis was independent of the analysis used to derive the intronic phylogeny in Figure 2B.

While the majority of shared micro-indels supported intronic tree in Figure 2B, some shared micro-indels were inconsistent with the intronic tree (i.e. “homoplasic”). These did not appear to be scattered randomly along the alignment; rather, the homoplasic micro-indels were concentrated in regions where sliding window analysis and computational methods also suggest gene conversion. For example, the majority of the homoplasic indels present in macaque introns co-localize to a region of intron 3, 4 and 5 also identified as a candidate region for gene conversion using pairwise similarity plots (described in detail below, Figure 5).

Indels are rare events, and micro-indels within introns are not likely favored or disfavored by selection pressures. It is unlikely, therefore, that shared micro-indels would result from convergent evolution (making them phylogenetically useful characters). The correlation between regions of gene conversion (identified via similarity plots and computational methods) and homoplasic indels suggests that homoplasic indels themselves are useful markers of gene conversion events, particularly when gene conversions are too small or too ancient to be detected by similarity plots or computational methods. Applying multiple detection strategies provided evidence for many gene conversions (Figure 5, S8 and S9). We focused our attention on regions with the strongest evidence of gene conversion where multiple methods suggested corroborating gene conversions.

For intronic regions, we focused on regions where pairwise similarity scores exceeded 0.96. The average pairwise similarity between intronic regions of *ADH1* orthologs that diverged at the human/macaque split ca. 23–34 Mya is 0.938+/−0.018 (Figure S13), and since (as discussed above) the *ADH1* paralogs diverged much before this, any region between two paralogs that score >0.96 are good candidates for gene conversion within the past 23–34 million years. We then looked for at least one other indicator of gene conversion in that region, either from a homoplasic micro-indel or one of the computational methods (RDP, GENECONV, or Bootscanning).

Computational methods identified within introns many additional potential gene conversion events that did not contain regions with similarity scores >0.96. These putative gene conversions presumably are of more ancient origins, occurring before (or very near) the time when the macaque and human lineages split ca. 23–34 mya. These putative gene conversion events are more speculative, and thus they are listed separately (Table S7).

The *ADH1* exonic sequences have less sequence data than their introns and have much fewer selectively neutral sites, making detection of gene conversion more difficult (selection pressure can lead to sequence conservation and convergent evolution, both of which confound gene conversion detection). The criteria for identifying putative gene conversions within exonic regions was broadened, focusing on regions identified by at least one computational method. Regions where pairwise similarities appeared higher in some pairs relative to other pairs in that region were also considered; if that pair also had a corresponding increase of pairwise similarity at synonymous-only sites in that region, a gene conversion of that region was considered more likely. If a putative gene conversion was near an exon:exon boundary, the flanking intronic regions were examined for a corroborating signal.

Conclusions drawn from exonic regions are often more tentative than those drawn from intronic regions. Independent analysis of introns can, however, support detection of gene conversion within exons when intronic gene conversions overlap intron-exon boundaries. Further, even if clear evidence of gene conversions is limited to within intronic regions, this nonetheless suggests gene conversions are also likely within exons where they are less clearly discerned.

An example of this combined analysis is described below for one region within the macaque paralogs.

#### Gene conversion between Mac_*ADH1.1* and *1.2* at intronic position 3700–5900 and flanking exons 3–5

A plot of the pairwise similarity among macaque intronic regions using a sliding window makes apparent a mosaic structure similar to that implied by the phylogeny of individual introns (Figure 5). For example, sequence similarity scores between Mac_*ADH1.2* and *1.3* are high at the beginning and end of the alignment, but drop in the region corresponding to intron 3–5 (intronic position 3700–5900). A complementary but opposite pattern is seen in the comparison of Mac_*ADH1.1* and Mac_*ADH1.2*. This type of mosaic structure is often taken as evidence of gene conversion [65], [66]. In this particular case, the most parsimonious explanation for the mosaic pattern entails a gene conversion of Mac_*ADH1.2* to Mac_*ADH1.1* from the beginning of intron 3 to the beginning of intron 5.

This pairwise-sliding window analysis was extended to the *exonic* regions, where a similar mosaic pattern corroborated the gene conversion evident in the intronic regions (Figure 5). This mosaic pattern was present even when examining only 4-fold degenerate (synonymous only) sites (Figure S14).

A gene conversion between the same paralogs and in the same intronic region was also identified by each of the three computational methods (RDP, GENECONV, and Bootscanning). Two of the computational methods identified the corresponding gene conversion in the intervening region of the exonic dataset.

As described above, micro-indels shared among intronic regions of paralogs are phylogenetically informative traits, the majority of which support the intronic tree. There are, however, 25 homoplasic micro-indels among the macaque paralogs; these are micro-indels that are inconsistent with the intronic tree. These are not scattered randomly along the alignment; rather, 20 of the 25 homoplasic micro-indels result from homoplasies between Mac_*ADH1.1* and Mac_*ADH1.2* and co-localize to the same region where sliding window analysis and computation methods identified gene conversion between Mac_*ADH1.1* and Mac_*ADH1.2* (Figure 5). This confirms homoplasic indels are useful markers of gene conversions, and may be particularly useful for identifying putative gene conversions too small or too ancient to be detected by similarity plots or computational methods.

When phylogenetic analysis of the exonic data was repeated with the gene converted region of Mac_*ADH1.2* masked by encoding it as “missing data” (nucleotide 172 to 534), Mac_*ADH1.2* changes phylogenetic location, becoming sister to Mac_*ADH1.3* (as in the intronic tree, data not shown).

Thus, analysis of the similarity plots and homoplasic micro-indels and multiple computational methods offer corroborating evidence of a gene conversion between Mac_*ADH1.2* and *1.3* that includes both intronic and exonic regions. This gene conversion is longer than most reported gene conversions, and may in fact be the result of a chromosomal recombination (which are formed by a different mechanism, but is otherwise nearly indistinguishable from a gene conversion).

#### Numerous gene conversions occur between macaque, human and marmoset *ADH1* paralogs

Applying this analysis to other parts of the macaque intronic alignment identified evidence for perhaps three additional gene conversions (summarized in Table 4). Two of these gene conversions are near intron:intron boundaries, and analysis of the neighboring exonic regions substantiate the spread of these gene conversions into the adjacent exonic regions.

**Table 4:** Summary of putative gene conversions among macaque, human and marmoset paralogs.

			evidence supporting putative gene conversion					
GeneconversionEvent ID	Paralogs involved in geneconversion	Region of multiple sequencealignment involved in geneconversion	Similarity plots	Micro-indels	RDP	GENECONV	Boot-scanning	Corroborating adjacentexon/intron
	**Macaque**							
1	Mac_*ADH1.1* and *1.2*	Intron 3, 4 and 5; bases 3740–5812	X	X	X	X	X	Yes, exons 3, 4, and 5
		**Exons 3, 4, and 5**	X	n/a	X	X		Yes, intron bases 3740–5812
2	Mac_*ADH1.0* and *1.1*	Intron 3; bases 4725–5199; (potentiallyincluding 2000–2500 and 3200–3600)	X	X	X	X	X	n/a
3	Mac_*ADH1.3* and *1.4* (equivalent to Mac_*ADH1.2* and *1.4*)	Intron 5 and 6, bases 7288–7425	X	X		X		Yes, exon 6
		**Exon 6**; Potentially homologous geneconversion in human, implying thispre-dates mac/human split (Table S7).	X			X	<0.90	Yes, intron bases 7288–7425
4	Mac_*ADH1.1* and *1.3* (equivalent to Mac_*ADH1.1* and *1.2*)	Intron 6; bases 7436–7866; (potentiallyincluding 6900–7100)	X		X	X	X	yes
		**Exon 6**	X	n/a	X	X		Yes, intron bases 7436–7866
5	Mac_*ADH1.0* and *1.3* (equivalent to Mac_*ADH1.0* and *1.2*)	**Exon 5**	Not at 4-fold sites	n/a		X	X	
6	Mac_*ADH1.0* and *1.4*	Intron 7 and 8, bases 9340–9515			X	X	X	Yes, exon 7
		**Exon 5 and/or 6**	Not at 4-fold sites	n/a		X		Yes, intron bases 9340–9515
	**Human**							
7	Hum_*ADH1*A and 1C	Intron 1; bases 79–222	X			X	X	n/a
8	Hum_*ADH1*B and 1C	Intron 5; bases 5563–5771	X	X	X	X	X	Yes, exon 4 and 5
		**Exon 5** (and potentially including from exon 3 to 7)	X	n/a	X	X		Yes, intron bases 5563–5771
9	Hum_*ADH1*A and 1B	Intron 5; bases 6759–7241	X			X		n/a
10	Hum_*ADH1*A and 1B	Intron 8; bases 11,167–11,326 and11,772–11,954	X			X		n/a
	**Marmoset**							
11	Cal_*ADH1.1* and *1.2*	Intron 3; bases 3682–4466 (potentially extending to 5300)	X	X		X	X	
12	Cal_*ADH1.3* and *1.4*	Intron 4; bases 5346–5612	X		X	X	X	Yes, exon 5
		**exon 5**	X	n/a	<0.90	X	X	Yes, intron bases 5346–5612
13	Cal_*ADH1.2* and *1.3*	Intron 5; bases ca. 5450–5800	X	X				
14	Cal_*ADH1.3* and *1.4*	Intron 6; bases 8515–8825	X		X	X	X	
15	Cal_*ADH1.2* and *1.4*	Intron 7; bases 9808–10,085	X	X	X	X	X	
16	Cal_*ADH1.2* and *1.4*	**Exon 3 and/or 4**	X	n/a	X	X	<0.90	
17	Cal_*ADH1.2* and *1.3*	**Exon 4 and/or 5**	Not at 4-fold sites	n/a	X	X	<0.90	
18	Cal_*ADH1.1* and *1.3*	**Exon 5 and/or 6**	X	n/a	<0.90	X	<0.90	
19	Cal_*ADH1.1* and *1.2*	**Exon 6**	X	n/a		X		

One of the most striking features of macaque exonic analysis centered around exon 6. While most regions of the macaque exons have a pairwise similarity scores <0.95, the region encompassing most of exon 6 displays much greater similarity (>0.96) among all paralog comparisons (Figure 5). This pattern is also apparent when only four-fold redundant (synonymous) nucleotide positions were examined (Figure S14), and therefore is not likely due to adaptive convergence or conservation of functionally critical residues. Computational methods also identify potential gene conversions in this region between Mac_*ADH1.1* and *1.3*, between *ADH1.2* and *1.4* and between *ADH1.1* and *1.4* (although some at only statistically marginal levels, p<0.10). Taken together, the evidence suggests repeated gene conversions homogenized this region among all macaque paralogs.

Analysis of *intronic* regions identified at least one gene conversion involving the Mac_*ADH1.0* pseudogene. Computational methods also identify two gene conversions that involve the Mac_*ADH1.0* pseudogene within *exonic* regions (similarity scores of synonymous sites show no marked increase in the corresponding exonic regions, suggesting the gene conversions identified by the computation methods must be quite ancient). These observations are noteworthy in that they suggest, even though Mac_*ADH1.0* is presently a pseudogene, it could have altered the evolutionary fate of its paralogs in the distant past. This has implications for the human *ADH1* genes, where the absence of a Mac_*ADH1.0* ortholog in the modern human genome cannot rule out gene conversions that pre-date the loss of the gene.

A similar multi-modal approach was applied to the human *ADH1* paralogs, where evidence was found for at least four gene conversions within intronic regions (Figure S8, summarized in Table 4). One of the gene conversions identified among the intronic regions of human *ADH1B* and *1C* is substantiates a corresponding gene conversion in the adjacent exonic region.

When this criteria is applied to marmoset paralogs, gene conversions of at least five *intronic* regions are identified by multiple methods (two of these gene conversions extend into the adjacent exon). Computational methods and similarity plots suggest four additional gene conversions within *exonic* regions (Figure S9, summarized in Table 4).

Five homoplasic micro-indels are found among marmoset paralogs that are associated with a marked increases (>97%) in sequence similarity of the corresponding paralogs, suggesting gene conversion of these regions within the past 23–34 My. The nine other marmoset homoplasic micro-indels, however, are *not* associated with a marked increase in sequence similarity, indicating the gene conversions that generated these micro-indels were either ancient or very short and therefore missed by our criteria. The nine marmoset gene conversions summarized in Table 4, therefore, likely represent an underestimate of the number of gene conversion that occurred since the paralogs duplicated.

The many gene conversions that have homogenized regions among the marmoset paralogs, in combination with fewer number of informative characters within exonic data, can explain why the marmoset paralogs clade closer to each other than to their catarrhine orthologs in the exonic phylogeny (Figure 2A).

### Synthesizing Observations into a Model for *ADH1* Paralog Duplication History

The identification of gene conversions in exonic and intronic regions strengthens the conclusion, drawn from the intronic tree in Fig. 2B, that four *ADH1* paralogs existed prior to the Catarrhine-Platyrrhine split and that one of these paralogs was subsequently lost in humans (and became a pseudogene in macaque). Indeed, evidence for gene conversions offers an explanation for the abundant homoplasies found in the exonic sequences when modeled under any tree topology, and the discrepancy between the exon and intron tree, especially when considering the lower information content in the exonic dataset. They do suggest that the model for the natural history of *ADH1* paralogs in Figure 2B should be adjusted to include specific gene conversion events.

**Figure 6:**
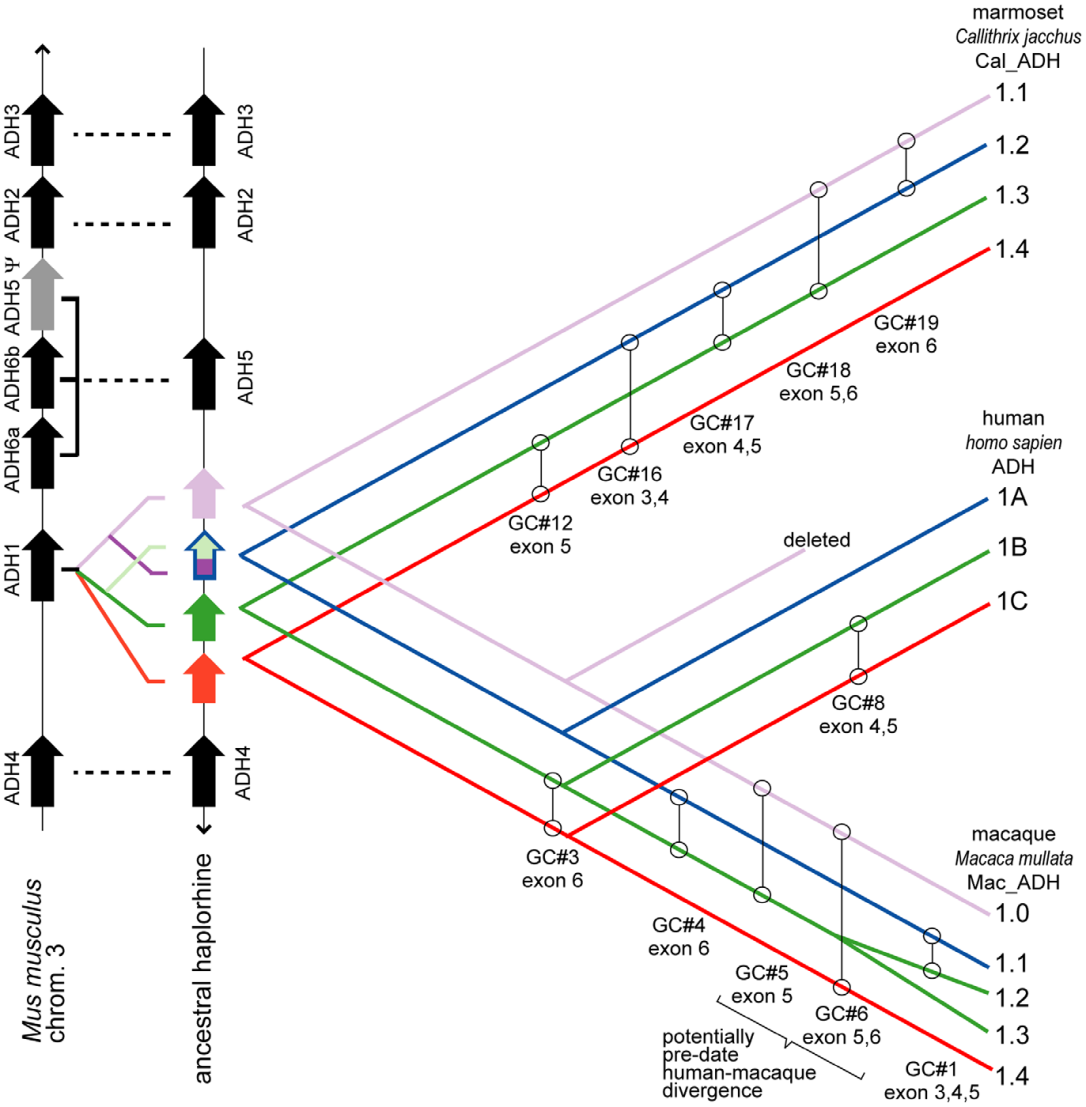
Model of *ADH1* paralog duplication and subsequent evolution in haplorhines. Thin vertical black arrows indicate the direction of the chromosome while thick vertical arrows identify ADH genes in the direction of transcription, with *ADH1* paralogs in primates colored according to the intronic phylogeny in Fig. 2B. Dashed lines connect orthologs. Diagonal lines indicate the proposed phylogeny of haplorrhine *ADH1* paralogs. The root of the haplorhine *ADH1* tree is not specified because the duplication order of haplorhine *ADH1* paralogs is ambiguous (see text). Putative gene conversions are indicated with open circles connected by vertical lines (from Table 4).

For example, in this modified model, Mac_*ADH1.2* and *1.3* are the most recent duplicates of a single gene. Following that duplication, a gene conversion event replaced a region from exon 3 to 5 of Mac_*ADH1.2* with the corresponding sequence of Mac_*ADH1.1*. This model is more parsimonious that a model where Mac_*ADH1.2* shares recent duplication event with Mac_*ADH1.1* followed by two gene conversion events (one at the 5′-end and one at the 3′-end) with Mac_*ADH1.3*. In other cases, the directionality of a gene conversion is not always clear. Figure 6 summarizes the modified model of *ADH1* paralog evolution.

It is commonly assumed that gene duplications create a new paralog by copying a single gene, rather than by fusion of parts of two existing paralogs [59], [14]. It is not clear, however, if this assumption is valid for all *ADH1* paralogs. Distance estimates suggest Mac_*ADH1.1* and Cal_*ADH1.2* (and presumably human *ADH1*A) could be chimeric genes, with the 5′-half of the chimeric gene duplicated from Mac_*ADH1.0* and Cal_*ADH1.1* (respectively), and the 3′-half duplicated from Mac_*ADH1.3* and Cal_*ADH1.3* (Table S6 and Figure S15). The evidence supporting this chimeric duplication is tentative, at best, and this signal could also reflect gene conversion history rather than a chimeric duplication. Our model in Figure 6 nonetheless incorporates the possibility that haplorhine ancestral of Cal_*ADH1.2* is a chimeric duplicate.

We attempted to determine the root of the haplorhine *ADH1* tree using intronic sequences from a variety of outgroups. The lemur genome sequencing projects are incomplete, providing only partial lemur *ADH1* intronic fragments. More distant outrgroups like rat and dog are so divergent from the primate *ADH1* paralogs that alignments are of poor wuality, containing many indels and only relatively short regions with unambiguous homology. Consequently, the phylogenies of individual introns have only weak support at many nodes (Figure S15). This was not unexpected given our earlier conclusion, based on the similar distances among all intra-species paralog comparisons, that the series of duplications yielding the four ancestral *ADH1* paralogs occurred in relatively rapid succession. Further, what little phylogenetic signal that accrued in the interval between duplications may have since been completely obscured by subsequent gene conversions. Nonetheless, it is noteworthy that the location of the root in these various phylogenies differed among the various introns. This prevents any strong conclusions about the order of gene duplication, but also indicate different phylogenies for the 5′- and 3′- halves of Mar_*ADH1.2* and its macaque ortholog (Mac_*ADH1.1*), suggesting the ancestral paralog may have been created by a chimeric gene duplication event. While a recombination event or repeated gene conversions in the ancestor of macaque and marmoset could explain this, we tentatively assume this pattern derives from a chimeric duplication event.

## Discussion

### The Last Common Ancestor of Old and New World Primates had Four Paralogous *ADH1*s

The literature describes many disagreements surrounding the natural history of primate ADH paralogs. These include disagreements concerning the number of paralogs, the topology of the tree describing their phylogeny, and the history of gene conversion between them. These disagreements carry forward to disagreements about the function of different paralogs catalyzing “the same” reaction, their natural substrates, their roles in fetal development, their roles in human alcoholism and related diseases, and how their behaviors have changed in response to a changing environment.

The additional data and analysis provided here help explain why these disagreements exist and how they might be resolved. The analysis requires, however, that stochastic bioinformatic tools provide only the starting point for analysis. To these must be added tools that consider non-canonical processes, including gene conversion, functional convergence, and database error. Only with these can the model for the natural history of primate *ADH1* paralogs be adequate to support evolution-based interpretation and paleogenetic studies.

First, disagreements over the preferred tree topology are hardly surprising if the topologies generated from an analysis of exon sequences differs from those generated by analysis of intron sequences. These differences might suggest that functional constraints operated strongly on the sequence divergence of *ADH1* paralogs in a way that creates homoplasies that conceal the “true” natural history. Conversely, these might indicate gene conversion are found more in exons than introns, or introns than exons, by accident, because of the mechanism of gene conversion (favoring conversion between more similar sequences), or selection pressure.

We have come to prefer the tree topology that is favored by intron sequence analysis (Figure 2B) over the topology indicated by the exonic sequence analysis (Figure 2A). This preference comes not only from the greater number of informative characters provided by intron sequences, or our suspicion that selective pressures have confused the phylogenetic signal carried by coding sequence (in fact, our analysis indicates the exonic phylogeny shown in Fig. 2A is largely independent of selective pressures, as analysis of synonymous sites only yields the same phylogeny). Rather, support of the intronic phylogeny comes from molecular clock estimates that establish the series of duplications that gave rise to four paralogs in marmoset and in macaque (including Mac_*ADH1.0*, but only one of the Mac_*ADH1.2*/*1.3* pair) predates the catarrhine-platyrrhine split. Further support comes from phylogenetic analysis of micro-indels, supplemented by the recognition that indel events in non-coding regions suffer only rarely from convergence. Finally, identification of specific gene conversions account for the many homplasic sites (both within synonymous and nonsynonymous sites, and micro-indels within introns), the discordant exonic and intronic phylogenies, discordant phylogenies for introns from the same gene, and underestimates of paralog duplication dates when estimated from synonymous sites of exonic data (Text S1).

Thus, our analysis suggests the intronic phylogeny in Figure 2B approximates the underlying phylogeny of gene duplication. The evolution of the exonic regions parallels this gene duplication history, but is modified by at least eleven independent gene conversions that involve exonic regions (five in macaque, one in human, and five in marmoset; Figure 6). Most of the exonic gene conversions are not associated with pronounced increases in pairwise similarity scores at synonymous sites exon 6 of the macaque paralogs are the exception). Therefore, if these gene conversions are not false positives, they occurred in the distant past. If so, we expect similar analysis will also identify comparable gene conversions in closely related primates.

It is important to emphasize that computation approaches used for identifying gene conversions are highly dependent on the model parameters, and different methods yield different results [64]. Identifying very ancient gene conversions results in only tentative conclusions. Because exonic regions have less sequence information and are subject to selective pressures, evidence supporting gene conversions in the distant past is usually considerably weaker and controvertible. For example, some have argued that gene conversion occurred among the pancreatic RNAse paralogs following their duplication in the Colobine monkeys [67]–[69]. Others have argued that the homoplasies within the pRNASE coding regions derive from convergent evolution for similar physical properties [70]–[72]. In the case of the primate ADH1 family, our conclusion that the exonic phylogeny is corrupted by gene conversions is not based on the reliability of the computational methods that identify specific gene conversions, but because multiple gene conversions offer a more plausible explanation than convergent evolution for the many observations described herein.

Our model places the formation of four *ADH1* paralogs before the divergence of catarrhines and platyrrhines (Old and New World primates), and *after* the divergence of strepsirhines (lemurs) from haplorhines (see Text S2). This places the duplication events in the Paleocene or Eocene, a period just following the K/T boundary and late Cretaceous, and also period of time when both paleontology and paleogenetics suggest that ethanol was becoming abundant in the biosphere.

One of the four *ADH1* paralogs was subsequently lost in the catarrhine lineage (becoming an apparent pseudogene in macaque, and deleted entirely from the genome of human). A fifth paralog arose by a unique (and therefore relatively recent) duplication in the OWM lineage leading to macaque, giving rise to Mac_*ADH1.2* and *1.3*. Substantial parts of Mac_*ADH1.2* were subsequently converted to Mac_*ADH1.1.*

The root of the primate *ADH1* tree is, as of yet, undetermined, owing to the rapidity of the paralog duplications and subsequent gene conversions. Even upon completion of intronic sequences from a near outgroup (e.g. a lemur or tree shrew), the order of paralog duplication may remain obscure. Further, pairwise distances and phylogenies containing distant outgroups suggest, albeit weakly, that Mar_*ADH1.2* and its macaque ortholog (Mac_*ADH1.1*) may have been created by a chimeric gene duplication event.

Even though this model appears to be robust, and appears to consider and manage many of the features of gene and proteins sequence divergence that can defeat even the most sophisticated bioinformatics tools, it remains a hypothesis. Nevertheless, because the hypothesis is robust, it can serve as the starting point for paleogenetics resurrections and experiments. These are presently under way.

### How the Current Model Compares to Earlier Models of *ADH1* Evolution

The proposed gene conversion events can be combined with yet another factor, database incompleteness and error, to resolve the long-standing controversies relating to the natural history of the *ADH1* family. We begin by comparing the contribution of Cheung *et al.* [31], which proposed gene conversion within *ADH1* paralogs, with the contribution of Oota *et al.* [32], which concluded that no evidence supported gene conversion.

First, lacking the benefit of marmoset or macaque genome sequence data, Cheung *et al.* [31] assumed that three *ADH1* paralogs existed in Old World monkeys (as with humans). Because of this assumption, they mistakenly concatenated the 5′-UTR of Mac_*ADH1.1* with the coding region of Mac_*ADH1.2*. This error created an artificial chimera (which they called “OWM_*ADH1*”) between the macaque paralogs of human *ADH1*A and human *ADH1*B. Not surprisingly, these authors also inferred different natural histories for the 5′-UTR of this construct (which we now see arises from Mac_*ADH1.1*) and the rest of the gene (which is from Mac_*ADH1.2*).

This chimera is particularly confusing because their “OWM_*ADH1*” sequence is, through gene conversion, a chimera of a chimera. The coding region of “OWM_*ADH1*” (our Mac_*ADH1.2*) has a piece of Mac_*ADH1.1* in its exon 3–5 region because of gene conversion. Hence, their analysis of “OWM_*ADH1*, exon 2–5” corresponds to a region that is most closely orthologous to human *ADH1*A (or, to use Cheung’s terminology, “*ADH1*”). Cheung’s conclusion that exon 2–5 did *not* suffer gene conversion in their “OWM_*ADH1*” follows from this artificial chimerality. This chimera had, in effect, already incorporated the gene conversion.

Similarly, the Cheung *et al.* analysis of exon 7–9 and the 3′-noncoding region suggested “OWM_ADH2” had converted to OWM_*ADH1*. However, this region of “OWM_*ADH1*” corresponds to Mac_*ADH1.2*. The clading of OWM_*ADH1* and OWM_ADH2 (Mac_*ADH1.3*) is *not* an indicator of gene conversion because both genes derive from a recent duplication in the Old World monkeys.

Cheung *et al.* [31] also inferred a unique gene conversion in the apes that transferred information to exon 2–5 of human *ADH1*C (their “ADH3”) from *ADH1*B (their “ADH2”). This putative conversion does not involve the artificial chimeric “OWM_*ADH1*” (which we believe is 5′-UTR of Mac_*ADH1.1*+ coding region of Mac_*ADH1.2*). Indeed, our analysis detected a similar region of increased similarity between human *ADH1*B and *ADH1C* (Figure S8).

Like Cheung, et al, Oota *et al.* [32] also assumed only three *ADH1* paralogs exist in Old World Monkey. Like Cheung *et al.* [31] Oota *et al.* [32] did not include any New World monkeys in their analysis. Further, their analysis included, we believe, an incomplete and inaccurately assigned set of paralogs for the Old World monkey outgroup. For example, their *intron* dataset (introns 2, 3, and 8) included only three Old World monkey (baboon) paralogs, corresponding to Mac_*ADH1.1*, Mac_*ADH1.3* and Mac_*ADH1.4*. Their *exon* dataset included human and chimp *ADH1*A-C, a single macaque paralog (which they called “*ADH1*A”, but was evidently Mac_*ADH1.2*), and two baboon paralogs (which they called *ADH1*B and *ADH1*C; our analysis suggests this assignment is correct, as they appear to be orthologs of Mac_*ADH1.3*/human_*ADH1*B and Mac_*ADH1.4*/human_*ADH1*C). Finally, because this analysis included less sequence data, and only considered large gene conversion tracts, it failed to identify several gene conversion events smaller than their analysis window.

### Do Gene Conversions Confer Adaptive Value?

With many gene conversions tentatively established that alter the coding regions of *ADH1* genes, we turn briefly to more speculative considerations about the functional impact of such gene conversions. For example, RDP identifies a gene conversion between *ADH1B* and *ADH1C* that includes exon 6 and part of exon 7 (Figure S8). Similarity plots also indicate increased similarity among *all* human paralogs in same region; increased similarity in this region is also seen among all paralogs when only synonymous sites are considered (Figure S16), suggesting gene conversion among all paralogs. Curiously, this is the same region where multiple macaque paralogs appear homogenized by multiple gene conversions (Figure 5), raising the possibility these gene conversions may predate the macaque/human divergence. Alternatively, independent gene conversions of the same region in both human and macaque lineages imply either adaptive value of these gene conversions, or this region is prone to gene conversion (presumably because it is highly conserved owing to functional constraints).

This region is already highly conserved among paralogs, and it may seem counterintuitive that additional “homogenization” by gene conversion in a region that is already nearly identical would have any discernable functional impact. However, the *ADH1*B*2, *ADH1*B*3 and *ADH1*C*1 polymorphisms illustrate that a single amino acid change occurring within the highly conserved NAD-binding domain can have very large functional impacts. In light of this, we might expect the most common gene conversions observed among paralogs would be those that promoted the transfer of a profoundly adaptive modification within a highly conserved region.

The exonic region from residue ca. 567–800 (the first half of exon 6) is very similar among primate and non-primate ADHs alike, relative to other parts of the protein. This region is predominated by residues involved in NAD binding, and as one expects, non-synonymous sites primarily account for this heightened similarity among non-primates (Figure S17), implying sequence similarity among primates and non-primates is driven by sequence conservation (purifying selection). Similarity among *non-primates* in the following region (ca. residue 800–925) drops off dramatically, but remains high in macaque and human. In fact, sequence similarity among human and macaque paralogs is at its highest in this region for *synonymous* sites, implying gene conversion may contribute (along with sequence conservation) to the increased similarity among paralogs in this region. Because this region is dominated by residues involved in subunit dimerization, increased sequence similarity (i.e. homogeneity) of this region suggests heterodimerization among macaque paralogs and among human paralogs has been selected for (relative to non-primates, where only a single paralog exists and heterodimers are not possible). The observation that synonymous site similarity is high in this region suggests gene conversions may have played a functional role to create the sequence homogeneity that permits (or maintains) heterodimerization.

Gene conversions among primate *ADH1* paralogs have occurred frequently within intronic regions, and judging from the pairwise similarity scores, some of these occurred recently (relative to paralog duplications, see Figure S9, panel E). This may owe to the multiple copies of each paralog, their sequence similarity, and/or their proximity in the genome. Alterations such as these gene conversions are expected to have minimal impact on the function of the affected gene when they occur within intronic regions, and are not likely to be disfavored by natural selection. Interestingly, gene conversions within *exonic* sequences are observed at a similarly high frequency (all appear ancient, except perhaps those affecting exon 6). Here, however, gene conversions affect substantial portions of the coding region that are under selection pressure.

Gene conversions within exonic regions that persist throughout the action of natural selection are expected to be of two types: those in which the fixation of gene conversions within a population are limited by natural selection to regions where the proteins were already homogeneous (both at sequence and functional levels) and therefore have no functional impact, or those where gene conversions altered functionally distinct residues but were favored by natural selection because they transferred beneficial changes from one paralog to another.

Distinguishing between the two scenarios is difficult without directly comparing ancestral sequences to modern sequences, but because so many of the gene conversions involve residues known to be involved in substrate specificity, NAD binding and subunit dimerization, it seems plausible that some of the gene conversions have conferred beneficial changes to the recipient gene. If this is borne out by future studies, then this would indicate that gene conversion among duplicated genes can function like recombination among orthologs to increase the rate of evolution of beneficial traits within a population.

### Conclusion

Earlier phylogenetic analysis of the ADH1 gene family assumed three ADH1 paralogs in both human and non-human primates, as well as their common ancestor [25]–[32]. Our database mining identified additional ADH1 paralogs in both marmoset and macaque. Our analsysis indicates the last common ancestor of Old and New World primates had four paralogous *ADH1*s. One of these four paralogs was subsequently lost in the human lineage and became a pseudogene in macaque, but remains active in marmosets. After diverging from the human lineage, yet another ADH1 duplication event occurred in the macaque lineage. Evidence for multiple gene conversions among ADH1 paralogs was found in each of the marmoset, macaque and human lineages.

The multiplicity of ADH1 paralogs and rampant gene conversion among paralogs obfuscates the evolutionary history of this gene family. The complicated history of this gene family required in-depth phylogenetic analysis and complete genomic record for key primates. With a more complete model for the natural history of the ADH1 gene family, new hypothesis regarding the role of ADH1 duplication (and loss) in the history of adaptation to dietary alcohols can be explored.

## Supporting Information

Figure S1 **Ensemble genome map of macaque (** ***M. mulatta*** **) covering the region including the four** ***ADH1*** **paralogs.** The Ensemble genome for macaque predicts ten overlapping alternatively spliced products (burgundy horizontal lines). The four non-overlapping *ADH1* genes used in this study are identified within the red boxes. Each gene is transcribed from right to left in this figure (i.e. the 5′ end of the coding region is on the right). The Mac_*ADH1.0* pseudogene is not shown, but is located adjacent and upstream (left) of the Mac_*ADH1.1* gene (see Table S1 for its precise genomic location).
(TIF)

Figure S2 **Phylogenetic relationship of ADH1 paralogs including lemurs.** The phylogeny of the *ADH1* paralogs shown here was determined by Bayesian analysis of exonic sequence data using a codon model, including strepsirrhines (lemurs, yellow: Ring-tailed lemur (*Lemur cata*), Brown lemur (*Eulemur fulvus collaris*), and Sifaka (*Propithecus coquereli*)), platyrrhines (New World primates, orange), and catarrhines (Old World primates and hominoids, red). The human *ADH2, ADH3, ADH4* and *ADH5* genes were used as representatives for mammalian ADH class I–V. Neither chicken (*Gallus gallus*) nor frog (*Xenopus tropicalis*) representatives of the mammalian ADH class II proteins were found in the public nucleotide databases, suggesting that either (1) these genes have not yet been sequenced in both chicken and frog, (2) the ADH class II homolog has been lost in both chicken and frog, or (3) the position of the human *ADH2* gene is incorrect in the phylogeny shown here (and should instead be sister to human *ADH3,* or branch after the chicken ADH Z and ADH Y clade). The names of the *ADH1* paralogs have been shortened (e.g. the marmoset (*Callthrix jacchus*) *ADH1* paralog “Cal_*ADH1.1*” is simply referred to as “marmoset ADH1.1”). Numbers at nodes refer to the Bayesian posterior probability values.
(TIF)

Figure S3 **Homplastic amino acid residues in the** ***ADH1*** **exonic dataset.** (A) The secondary structure information for the ADH1 protein (based on crystallographic data from ADH1 proteins [73], [74]) is shown. (B) Residues involved in NAD/NADH binding, subunit dimerization, substrate binding, and zinc interaction are highlighted (the numbering refers to the crystallographic number scheme, which does not include the first amino acid methionine; the color refers to the reference, shown at the far right). (C) The amino acid alignment and nucleotide alignment of the human, macaque and marmoset *ADH1* paralogs is shown (residues that are identical to the human ADH1A reference sequence are shown as a period). Residues that are homoplasic when the data is modeled according to the intronic tree (Figure 2B) are highlighted with color. Panel (E) shows the same analysis, but modeled using the exonic tree (Figure 2A). Sites that are homoplasic in both alignments are colored in red/yellow. Sites that are homoplastic only under the intronic evolutionary model are colored blue/orange, and sites that are homoplastic only under the exonic evolutionary model are colored green/purple. Sites that are homoplasic according to one phylogeny but informative according to the other phylogeny are highlighted lime-green in the alignment where they are informative. When more than one homoplasic residue exists within a codon, the second homoplasy is colored turquoise. (D) Homoplasies that are unique when the data is modeled according to either intronic or exonic phylogeny are indicated.
(XLSX)

Figure S4 **Phylogenic analysis of individual** ***ADH1*** **introns including fragments from lemur intronic sequences.** Lemur intronic sequences from mouse lemur (*Microcebus murinus)* and bushbaby (*Otolemur galago*) were assembled manually from sequences retrieved using a BLAST search of the NCBI Trace Archive WGS database for both *Microcebus* and *Otolemur* using human *ADH1A* as a query. These were aligned with the *ADH1* paralogs from macaque, marmoset and human, and Bayesian analysis was used to infer the most likely phylogeny for each available intron individually. Lemur paralogs form a single clade, indicated within a red box. Lemur intronic sequences are incomplete and not contiguous, so paralogs are arbitrarily number 1–3, and sequences from one intron are not necessarily from the same gene as identically numbers sequences from another intron. The names of the *ADH1* paralogs have been shortened (e.g. the marmoset (*Callthrix jacchus*) *ADH1* paralog “Cal_*ADH1.1*” is simply referred to as “marmoset *ADH1.1*”). Numbers at nodes refer to the Bayesian posterior probability values.
(TIF)

Figure S5 **Mapping of the homoplasic amino acids onto human** ***ADH1B*** **crystal structure.** The crystal structure of human *ADH1B* is shown, with homoplasic residues highlighted to illustrate their proximity to the active site. In all four panels, the NAD cofactor is colored red and the substrate analog is highlighted green. Residues in close proximity (by eye) to either NAD or the substrate analog are indicated below an asterisk; residues identified by Gibbons, *et al* as interacting with NAD or the substrate analog are indicated below with *italic* text [74]. (A) Homoplasic amino acids present in both the “exonic” and “intronic” phylogenies, part 1∶17 blue; 25 black; 41 bluetint; 47*brown; 48*cyan; 56*grey; 57*greenblue; 63 greentint; 84 hotpink; 93*magenta; 94*orange; 102 pink; 105 pinktint; 108 purple; 116*redorange; 117*seagreen; 120 skyblue; 128 violet; 133 white; 141*yellow; 143 yellowtint. (B) Homoplasic amino acids present in both the “exonic” and “intronic” phylogenies, part 2∶166 blue; 185 black; 207 bluetint; 285 brown; 291 cyan; 303 grey; 318*greenblue; 322 greentint; 327 hotpink; 330 magenta; 349 orange; 363 pink; 371 pinktint; 373 purple. (C) Homoplasic amino acids present only in the “intronic” phylogeny: 18 blue; 64 black; 152 bluetint; 155 brown; 275 cyan; 348 grey. (D) Homoplasic amino acids present only in the “exonic” phylogeny: 34 greenblue; 319*greentint; 328 hotpink.
(TIF)

Figure S6 **Phylogenetic analysis of exonic dataset after partitioning into synonymous and nonsynonymous datasets.** Neighbor-joining was used to determine the phylogeny of the exonic dataset after partitioning into two sets containing either (A) non-synonymous/informative sites (46 codons), or (B) the remaining sites (synonymous and non-informative non-synonymous sites, including 58 parsimoniously informative sites). The non-synonymous/informative dataset was created by first identifying the codons wherein two or more of the macaque, marmoset or human ADH1 sequences had a non-synonymous change (46 codons in total; three of these codons were non-parsimoniously informative non-synonymous changes, i.e. the position included two or more non-synonymous singletons, totaling 54 parsimoniously informative sites). If both amino acids involved in the non-synonymous change were coded by four-fold degenerate codons, then the third codon position was not a factor in creating the non-synonymous change, and was therefore included in the synonymous site partition (10 positions). The names of *ADH1* paralogs have been shortened (e.g. the marmoset (*Callthrix jacchus*) *ADH1* paralog “Cal_*ADH1.1*” is simply referred to as “marmoset *ADH1.1*”). Numbers at nodes refer to the bootstrap support values.
(TIF)

Figure S7 **Phylogenetic analysis of individual** ***ADH1*** **introns.** Phylogenetic trees produced by Bayesian analysis of all introns concatenated and each intron individually are shown. Branches are color coded according to the four primary clades established in the phylogeny of the concatenated intronic dataset (human ADH1A, ADH1B, ADH1C, and Cal_ADH1.1/Mac_ADH1.0). Intronic trees are not rooted using an outrgroup, The names of *ADH1* paralogs have been shortened (e.g. the marmoset (*Callthrix jacchus*) *ADH1* paralog “Cal_*ADH1.1*” is simply referred to as “marmoset 1.1”). Numbers at nodes refer to the Bayesian posterior probability values.
(TIF)

Figure S8 **Summary of gene conversion analysis for human** ***ADH1*** **paralogs.** Exonic and intronic data sets were examined for indicators of gene conversion using similarity plots, homoplasic micro-indels, and various computational methods. (A) The figure legend displays the color schemes used in subsequent panels for illustrating pairwise similarity scores among paralogs, and the key used to summarize the results from various methods used to identify potential gene conversions. The names of *ADH1* paralogs have been shortened (e.g. human *ADH1A* is simply referred to as “human 1A”). Pairwise similarity within a sliding window is plotted for various paralogs within (C) exonic regions and (E) intronic regions. The color of the line in the similarity plot corresponds to the identity of the paralog pair, as indicated in the figure legend (A). Similarity scores for exonic regions are calculated within a 150-nt sliding window, while that of intronic regions are calculated using a 250-nt sliding window. Colored boxes in (B) and (D) indicate putative gene conversion events identified by various computation methods. The color of the box corresponds to the computational method identifying each potential gene conversion, as indicated in the figure legend (A). The paralogs implicated in gene conversion are indicated within (or adjacent to) the colored box using the paralog suffix (e.g a gene conversion between human *ADH1A* and *1B* is indicated by “A:B”). Homoplasic micro-indels in the intronic sequences are shown as vertical black arrows with the paralogs sharing these micro-indels indicated above each each arrow. Boundaries between introns or exons are demarcated with dotted vertical lines. Green boxes below the similarity plots indicate large gaps in the alignment, with the affected paralog indicated within the box.
(TIF)

Figure S9 **Summary of gene conversion analysis for marmoset ADH1 paralogs.** Exonic and intronic data sets were examined for indicators of gene conversion using similarity plots, homoplasic micro-indels, and various computational methods. (A) The figure legend displays the color schemes used in subsequent panels for illustrating pairwise similarity scores among paralogs, and the key used to summarize the results from various methods used to identify potential gene conversions. The names of *ADH1* paralogs have been shortened (e.g. the marmoset (*Callithrix jacchus*) *ADH1* paralog “Cal_*ADH1.1*” is simply referred to as “Cal 1.1”). Pairwise similarity within a sliding window is plotted for various paralogs within (C) exonic regions and (E) intronic regions. The color of the line in the similarity plot corresponds to the identity of the paralog pair, as indicated in the figure legend (A). Similarity scores for exonic regions are calculated within a 150-nt sliding window, while that of intronic regions are calculated using a 250-nt sliding window. Colored boxes in (B) and (D) indicate putative gene conversion events identified by various computation methods. The color of the box corresponds to the computational method identifying each potential gene conversion, as indicated in the figure legend (A). Boxes with dashed borders indicate gene conversions that were not statistically significant at p-values <0.05, but were identified using p-values <0.10. The paralogs implicated in gene conversion are indicated within (or adjacent to) the colored box using the paralog suffix (e.g a gene conversion between Cal_*ADH1.1* and Cal_*ADH1.2* is indicated by “1∶2”). Homoplasic micro-indels in the intronic sequences are shown as vertical black arrows with the paralogs sharing these micro-indels indicated above each each. Boundaries between introns or exons are demarcated with dotted vertical lines. Green boxes below the similarity plots indicate large gaps in the alignment, with the affected paralog indicated within the box.
(TIF)

Figure S10 **Examples of micro-indels within intronic alignments.** Six sections from the multiple sequence alignment of the concatenated intronic data set are shown. Numbers below the alignment (“fragment base”) refer to the nucleotide position within the entire alignment. Gaps within individual sequences (denoted with “:” and highlighted blue) create indels. When the same indel occurred in two or more sequences, these shared micro-indels were scored as either “homoplasic” or “consistent” relative to the phylogenetic tree deduced from the entire dataset (as in Figure 2B, and shown to the left of each alignment subsection). (A) Examples of shared micoindels that are consistent with the evolutionary model deduced from the entire intronic dataset are indicated within a green box. (B) Examples of shared micoindels that are homoplastic with regard to the evolutionary model deduced from the entire intronic dataset are indicated within a red box.
(TIF)

Figure S11 **Summary of micro-indels in the concatenated intronic dataset coded as binary data.** Micro-indels that are generally informative for the human/OWM/NWM *ADH1A* clade are highlighted yellow. Similarly, micro-indels that are generally informative for the human/OWM/NWM *ADH1B* clade are highlighted green, and micro-indels that are generally informative for the human/OWM/NWM *ADH1C* clade are highlighted blue. Micro-indels that are generally informative for the Mac_*ADH1.0*/Mar_*ADH1.1* clade are highlighted red. Micro-indels that are generally informative for both the human/OWM/NWM *ADH1A* clade and the Mac_*ADH1.0*/Mar_*ADH1* clade are highlighted orange. Micro-indels that are suggestive of gene conversion are highlighted according to the source of the micro-indel (i.e. green if the micro-indel is generally informative of the human/OWM/NWM *ADH1B* clade), or highlighted purple, pink or grey when the source is undetermined. Micro-indels that are shared by both the Cal_*ADH1.1* and Cal_*ADH1.2* clade are highlighted orange. Missing data (from a gap in the alignment) is denoted with a period.
(XLS)

Figure S12 **Parsimony analysis of the micro-indels coded as binary data.** Micro-indels were coded as binary data, and parsimony analysis was conducted using MacClade to model the dataset according to either (A) the exon tree in Figure 2A, or (B) the intron tree in Figure 2B. The number of steps required to obtain the dataset when evolving according either model is shown below each tree. If the exon tree is modified by relocating Mac_*ADH1.2* sister to Mac_*ADH1.3* (as in the intronic tree), the parsimony score drops from 369 to 320 (tree not shown). This emphasizes the impact this single gene, and its associated gene conversion (see text) has on the overall parsimony score. The names of *ADH1* paralogs have been shortened (e.g. the marmoset (*Callthrix jacchus*) *ADH1* paralog “Cal_*ADH1.1*” is simply referred to as “marmoset *ADH1.1I”*).
(TIF)

Figure S13 **Similarity plot comparison of human** ***ADH1B*** **and its and macaque ortholog.** Pairwise similarity is shown among the intronic regions of the human *ADH1B*, human *ADH1C*, and macaque Mac_*ADH1.3* paralogs (window size = 250, minimum non-gapped positions = 200); the average of all pairwise distances across the entire alignment for this orthologous pair (human *ADH1B* and macaque Mac_*ADH1.3*) is 0.932+/−0.0174. The spike in pairwise similarity between human *ADH1B* and *ADH1C* at position 5900 and 6100 is presumably from a gene conversion (see text). The spike in pairwise similarity between human *ADH1B* and Mac_*ADH1.3* at 8200–8500 likely reflects a gene conversion where Mac_*ADH1.3* was converted to Mac_*ADH1.4*.
(TIF)

Figure S14 **Similarity plot of macaque** ***ADH1*** **exonic sequences partitioned by coding degeneracy.** Pairwise similarity between each macaque paralog is shown using (A) all sites (window size = 150), (B) only 0-fold degenerate sites (window size = 120 nt), and (C) using only 4-fold degenerate sites (window size = 35-nt).
(TIF)

Figure S15 **Phylogenetic analysis of individual** ***ADH1*** **introns with non-primate outgroups.** Neighbor-joining was used to determine the phylogeny of each intron individually after aligning with non-primate outgroups. The names of *ADH1* paralogs have been shortened (e.g. the marmoset (*Callthrix jacchus*) *ADH1* paralog “Cal_*ADH1.1*” is simply referred to as “marmoset *ADH1.1*”). Numbers at nodes refer to the bootstrap support values.
(TIF)

Figure S16 **Similarity plot of human** ***ADH1*** **exonic sequences partitioned by coding degeneracy.** Pairwise similarity is shown between all human *ADH1* paralogs using (A) all sites (window size 150), (B) only 0-fold degenerate sites (window size = 120 nt), and (C) using only 4-fold degenerate sites (window size = 35-nt).
(TIF)

Figure S17 **Similarity plot comparing exonic sequences (partitioned by coding degeneracy) of macaque** ***ADH1*** **paralogs to their rat ortholog.** Pairwise similarity between rat and macaque *ADH1* paralogs is shown using (A) all sites (window size = 150), (B) only 0-fold degenerate sites (window size = 120 nt), and (C) using only 4-fold degenerate sites (window size = 35-nt).
(TIF)

Table S1 **Accession numbers for** ***ADH*** **genes used in this study.**
(DOC)

Table S2 **Source of tissues used in this study.**
(DOC)

Table S3 **Primers used in this study.**
(DOC)

Table S4 **Summary of sequences retrieved from marmoset SRA and macaque EST databases.**
(DOC)

Table S5 **TREx estimates for paralog and ortholog ADH1 duplications.**
(DOC)

Table S6 **Pairwise distances among marmoset and macaque paralogs for individual introns and UTR regions.**
(DOC)

Table S7 **Putative gene conversions of ancient ancestry.**
(DOC)

Text S1 **Molecular clocks applied to exonic regions do not favor one phylogeny over the other.**
(DOC)

Text S2 **How many ADH1 paralogs existed in the urprimate?**
(DOC)
