# Supplementary figures and images for: The Natural History of Class I Primate Alcohol Dehydrogenases Includes Gene Duplication, Gene Loss, and Gene Conversion
Source: PLoS One. 2012 Jul 31;7(7):e41175. doi: 10.1371/journal.pone.0041175 (PMC3409193; doi:10.1371/journal.pone.0041175)

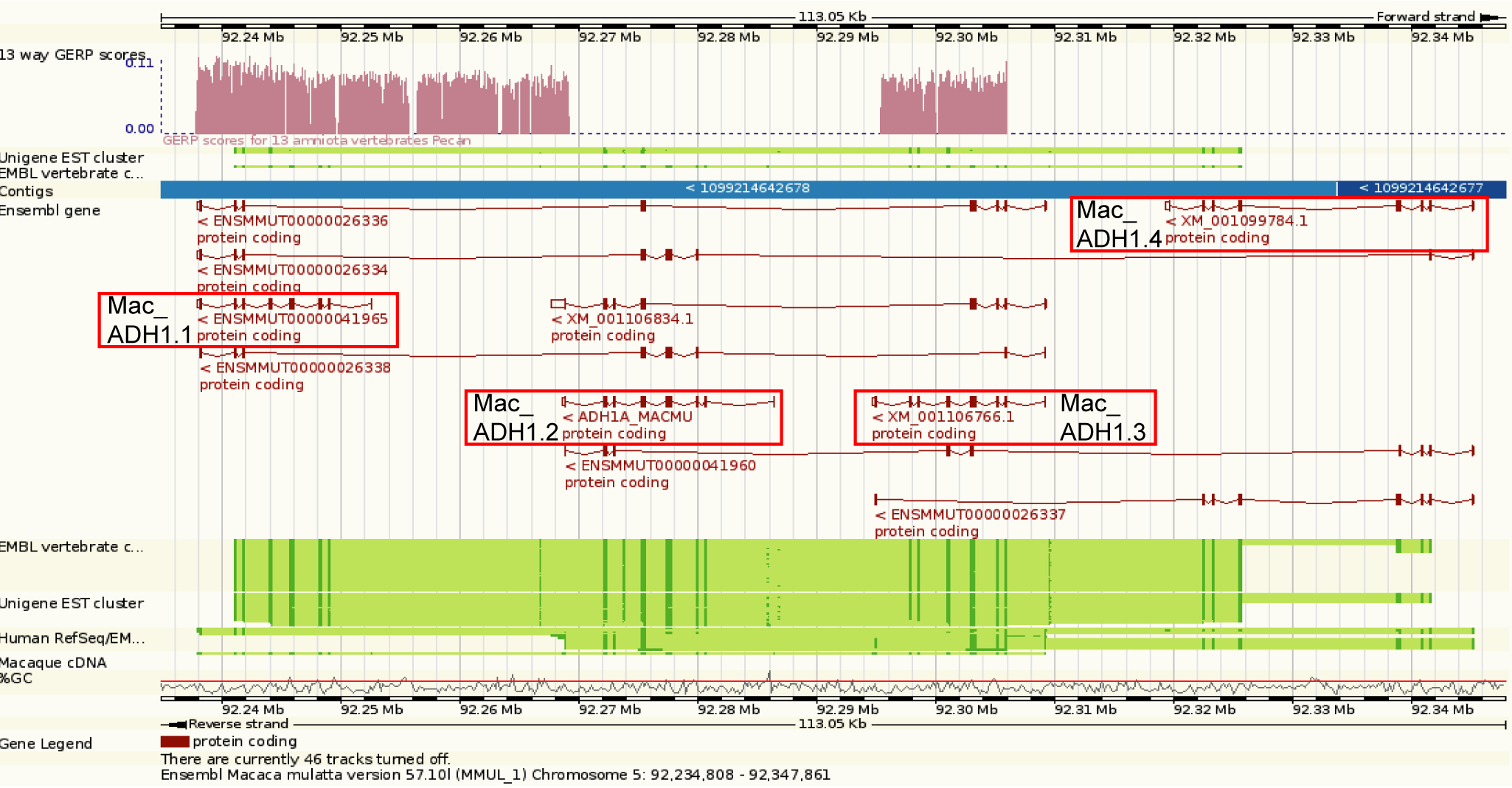

Supplement: Figure S1 — Ensemble genome map of macaque ( M. mulatta ) covering the region including the four ADH1 paralogs. The Ensemble genome for macaque predicts ten overlapping alternatively spliced products (burgundy horizontal lines). The four non-overlapping ADH1 genes used in this study are identified within the red boxes. Each gene is transcribed from right to left in this figure (i.e. the 5′ end of the coding region is on the right). The Mac_ADH1.0 pseudogene is not shown, but is located adjacent and upstream (left) of the Mac_ADH1.1 gene (see Table S1 for its precise genomic location). (TIF) [file pone.0041175.s001.tif]

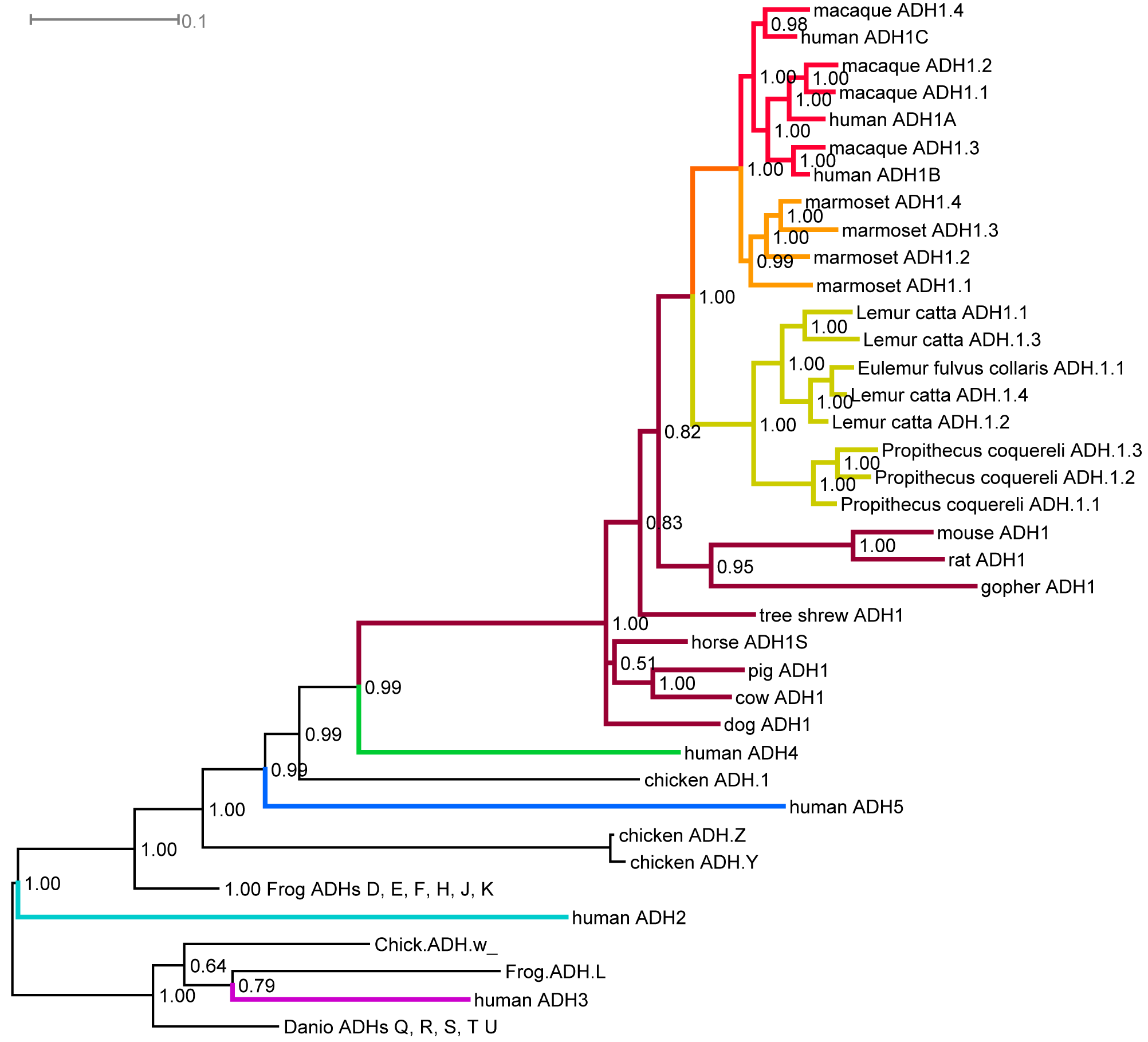

Supplement: Figure S2 — Phylogenetic relationship of ADH1 paralogs including lemurs. The phylogeny of the ADH1 paralogs shown here was determined by Bayesian analysis of exonic sequence data using a codon model, including strepsirrhines (lemurs, yellow: Ring-tailed lemur (Lemur cata), Brown lemur (Eulemur fulvus collaris), and Sifaka (Propithecus coquereli)), platyrrhines (New World primates, orange), and catarrhines (Old World primates and hominoids, red). The human ADH2, ADH3, ADH4 and ADH5 genes were used as representatives for mammalian ADH class I–V. Neither chicken (Gallus gallus) nor frog (Xenopus tropicalis) representatives of the mammalian ADH class II proteins were found in the public nucleotide databases, suggesting that either (1) these genes have not yet been sequenced in both chicken and frog, (2) the ADH class II homolog has been lost in both chicken and frog, or (3) the position of the human ADH2 gene is incorrect in the phylogeny shown here (and should instead be sister to human ADH3, or branch after the chicken ADH Z and ADH Y clade). The names of the ADH1 paralogs have been shortened (e.g. the marmoset (Callthrix jacchus) ADH1 paralog “Cal_ADH1.1” is simply referred to as “marmoset ADH1.1”). Numbers at nodes refer to the Bayesian posterior probability values. (TIF) [file pone.0041175.s002.tif]

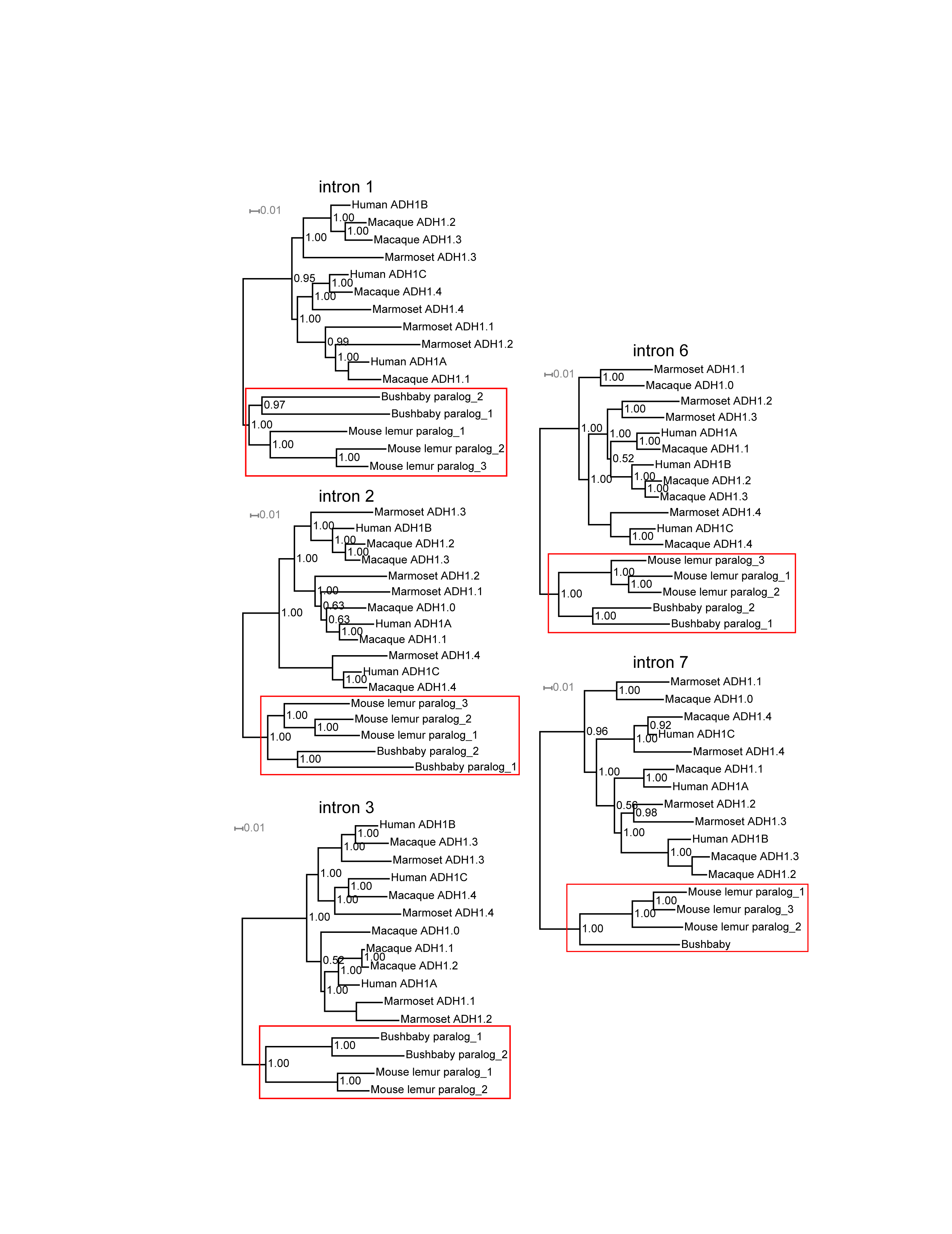

Supplement: Figure S4 — Phylogenic analysis of individual ADH1 introns including fragments from lemur intronic sequences. Lemur intronic sequences from mouse lemur (Microcebus murinus) and bushbaby (Otolemur galago) were assembled manually from sequences retrieved using a BLAST search of the NCBI Trace Archive WGS database for both Microcebus and Otolemur using human ADH1A as a query. These were aligned with the ADH1 paralogs from macaque, marmoset and human, and Bayesian analysis was used to infer the most likely phylogeny for each available intron individually. Lemur paralogs form a single clade, indicated within a red box. Lemur intronic sequences are incomplete and not contiguous, so paralogs are arbitrarily number 1–3, and sequences from one intron are not necessarily from the same gene as identically numbers sequences from another intron. The names of the ADH1 paralogs have been shortened (e.g. the marmoset (Callthrix jacchus) ADH1 paralog “Cal_ADH1.1” is simply referred to as “marmoset ADH1.1”). Numbers at nodes refer to the Bayesian posterior probability values. (TIF) [file pone.0041175.s004.tif]

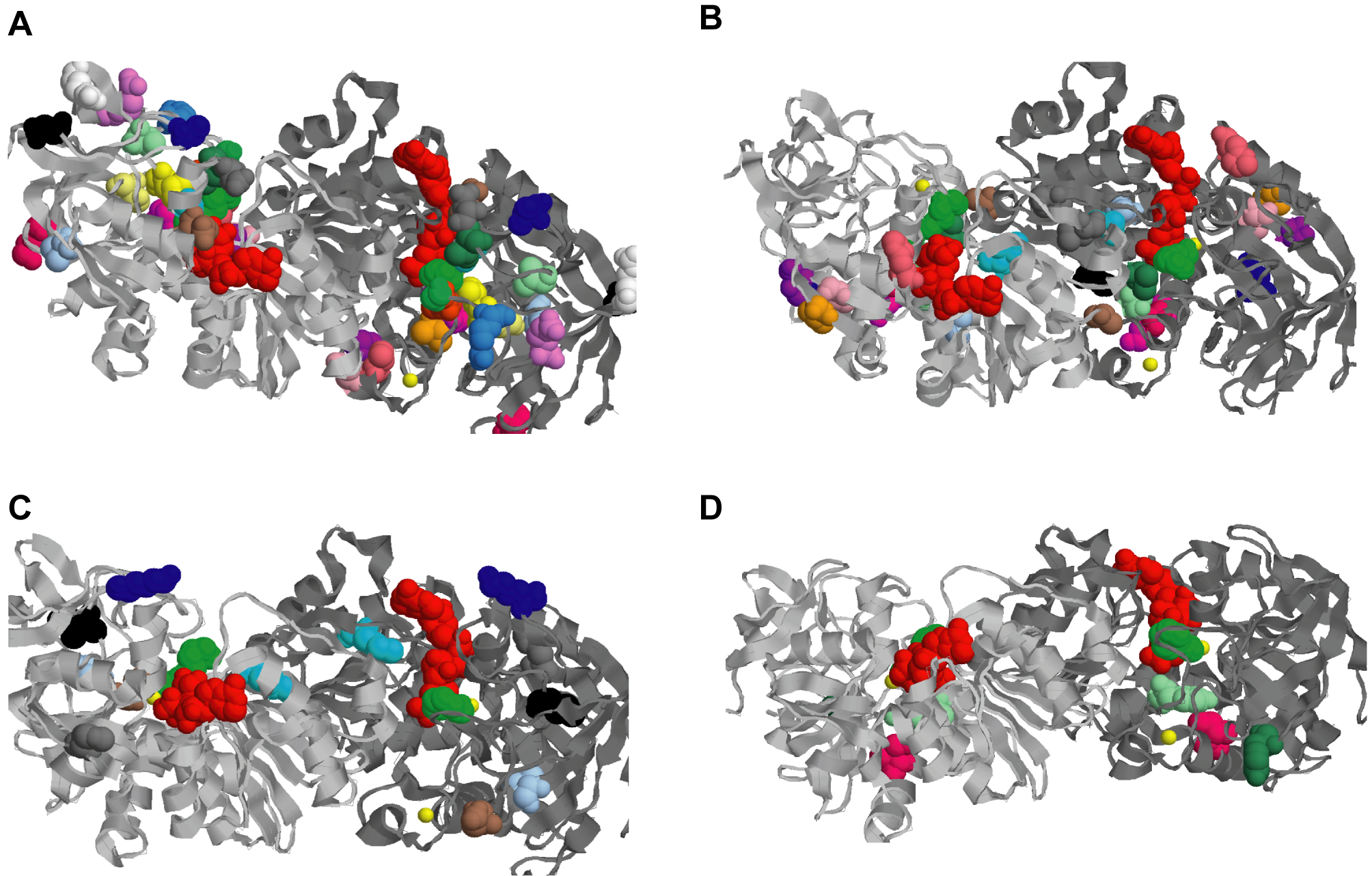

Supplement: Figure S5 — Mapping of the homoplasic amino acids onto human ADH1B crystal structure. The crystal structure of human ADH1B is shown, with homoplasic residues highlighted to illustrate their proximity to the active site. In all four panels, the NAD cofactor is colored red and the substrate analog is highlighted green. Residues in close proximity (by eye) to either NAD or the substrate analog are indicated below an asterisk; residues identified by Gibbons, et al as interacting with NAD or the substrate analog are indicated below with italic text [74]. (A) Homoplasic amino acids present in both the “exonic” and “intronic” phylogenies, part 1∶17 blue; 25 black; 41 bluetint; 47*brown; 48*cyan; 56*grey; 57*greenblue; 63 greentint; 84 hotpink; 93*magenta; 94*orange; 102 pink; 105 pinktint; 108 purple; 116*redorange; 117*seagreen; 120 skyblue; 128 violet; 133 white; 141*yellow; 143 yellowtint. (B) Homoplasic amino acids present in both the “exonic” and “intronic” phylogenies, part 2∶166 blue; 185 black; 207 bluetint; 285 brown; 291 cyan; 303 grey; 318*greenblue; 322 greentint; 327 hotpink; 330 magenta; 349 orange; 363 pink; 371 pinktint; 373 purple. (C) Homoplasic amino acids present only in the “intronic” phylogeny: 18 blue; 64 black; 152 bluetint; 155 brown; 275 cyan; 348 grey. (D) Homoplasic amino acids present only in the “exonic” phylogeny: 34 greenblue; 319*greentint; 328 hotpink. (TIF) [file pone.0041175.s005.tif]

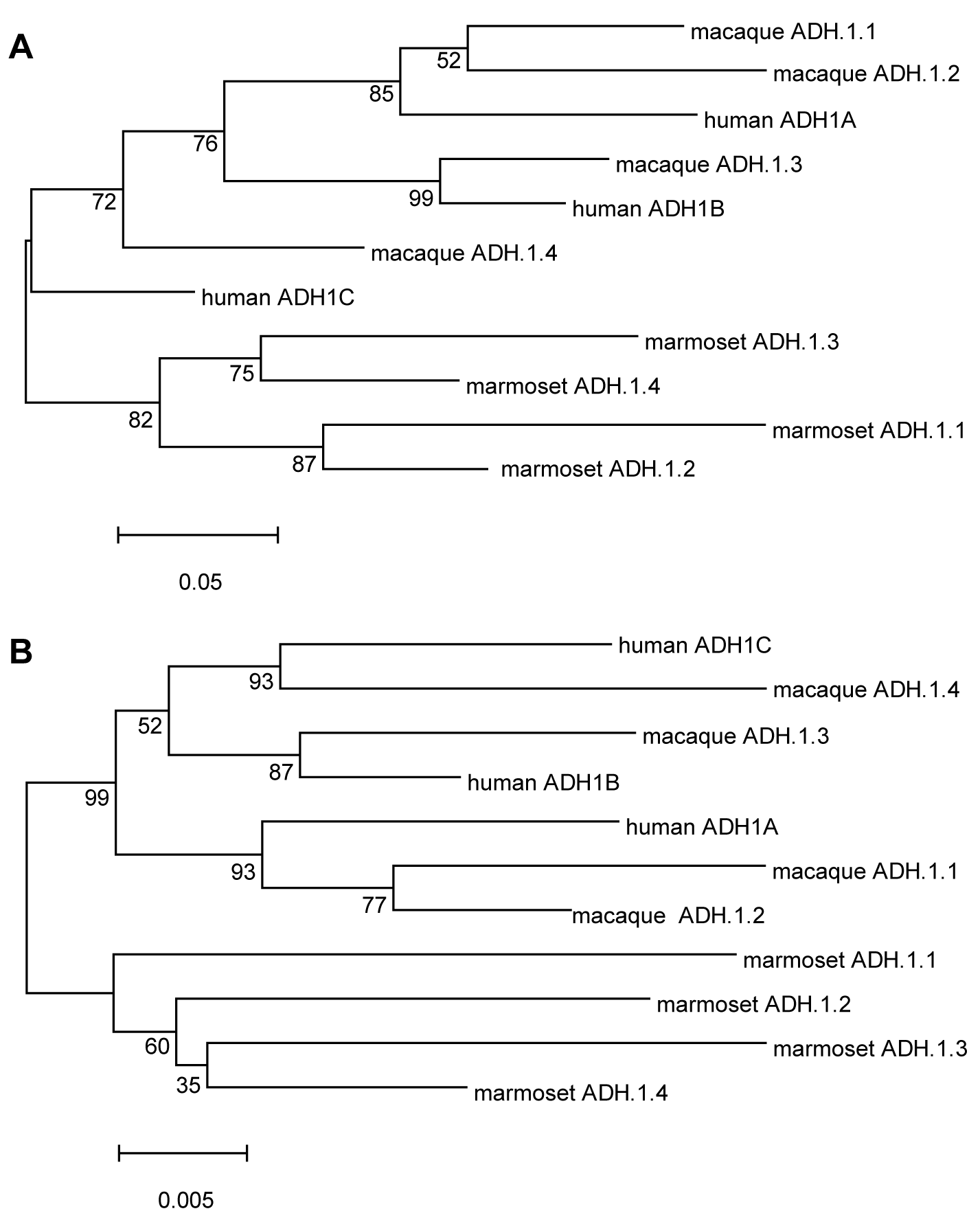

Supplement: Figure S6 — Phylogenetic analysis of exonic dataset after partitioning into synonymous and nonsynonymous datasets. Neighbor-joining was used to determine the phylogeny of the exonic dataset after partitioning into two sets containing either (A) non-synonymous/informative sites (46 codons), or (B) the remaining sites (synonymous and non-informative non-synonymous sites, including 58 parsimoniously informative sites). The non-synonymous/informative dataset was created by first identifying the codons wherein two or more of the macaque, marmoset or human ADH1 sequences had a non-synonymous change (46 codons in total; three of these codons were non-parsimoniously informative non-synonymous changes, i.e. the position included two or more non-synonymous singletons, totaling 54 parsimoniously informative sites). If both amino acids involved in the non-synonymous change were coded by four-fold degenerate codons, then the third codon position was not a factor in creating the non-synonymous change, and was therefore included in the synonymous site partition (10 positions). The names of ADH1 paralogs have been shortened (e.g. the marmoset (Callthrix jacchus) ADH1 paralog “Cal_ADH1.1” is simply referred to as “marmoset ADH1.1”). Numbers at nodes refer to the bootstrap support values. (TIF) [file pone.0041175.s006.tif]

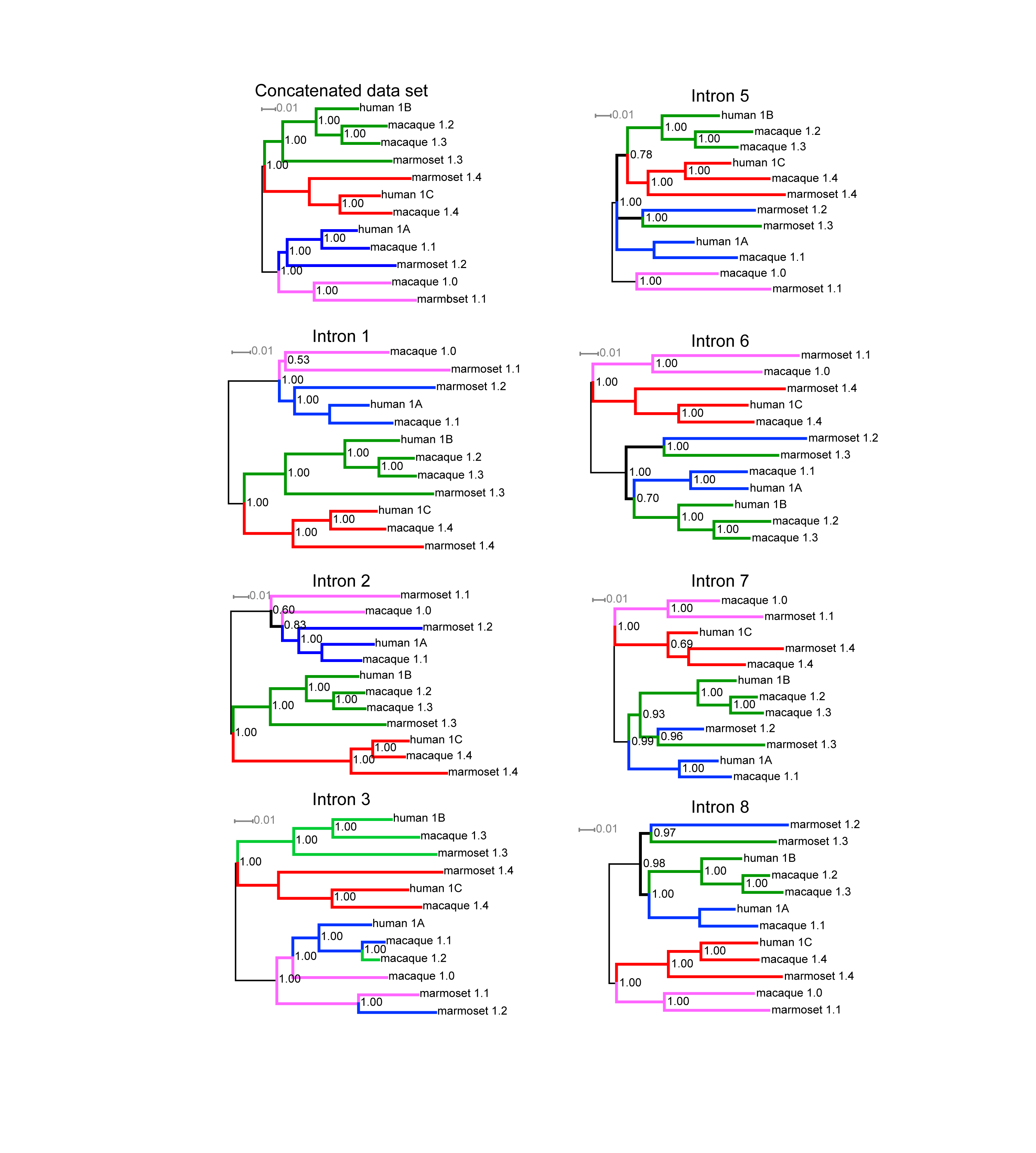

Supplement: Figure S7 — Phylogenetic analysis of individual ADH1 introns. Phylogenetic trees produced by Bayesian analysis of all introns concatenated and each intron individually are shown. Branches are color coded according to the four primary clades established in the phylogeny of the concatenated intronic dataset (human ADH1A, ADH1B, ADH1C, and Cal_ADH1.1/Mac_ADH1.0). Intronic trees are not rooted using an outrgroup, The names of ADH1 paralogs have been shortened (e.g. the marmoset (Callthrix jacchus) ADH1 paralog “Cal_ADH1.1” is simply referred to as “marmoset 1.1”). Numbers at nodes refer to the Bayesian posterior probability values. (TIF) [file pone.0041175.s007.tif]

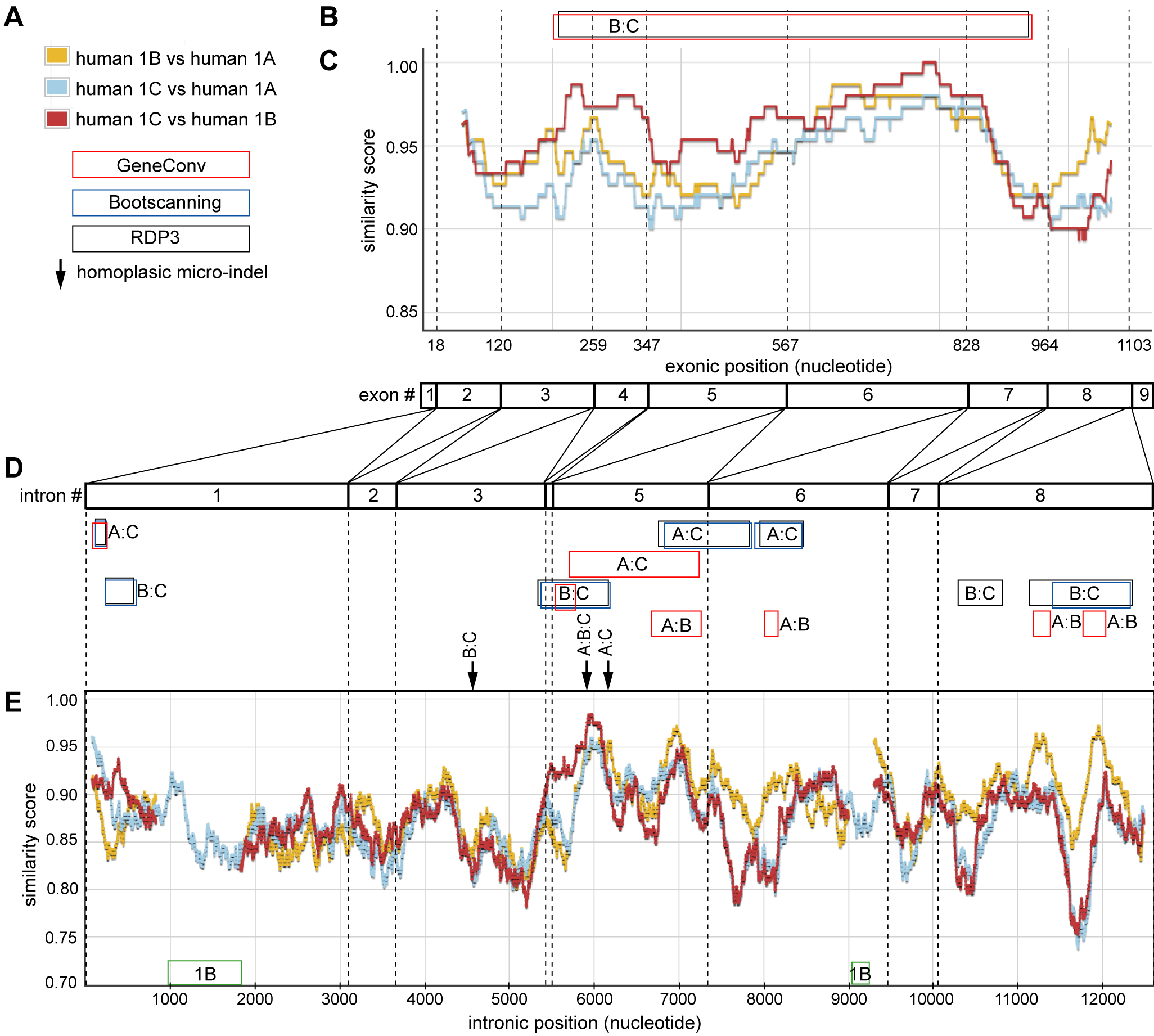

Supplement: Figure S8 — Summary of gene conversion analysis for human ADH1 paralogs. Exonic and intronic data sets were examined for indicators of gene conversion using similarity plots, homoplasic micro-indels, and various computational methods. (A) The figure legend displays the color schemes used in subsequent panels for illustrating pairwise similarity scores among paralogs, and the key used to summarize the results from various methods used to identify potential gene conversions. The names of ADH1 paralogs have been shortened (e.g. human ADH1A is simply referred to as “human 1A”). Pairwise similarity within a sliding window is plotted for various paralogs within (C) exonic regions and (E) intronic regions. The color of the line in the similarity plot corresponds to the identity of the paralog pair, as indicated in the figure legend (A). Similarity scores for exonic regions are calculated within a 150-nt sliding window, while that of intronic regions are calculated using a 250-nt sliding window. Colored boxes in (B) and (D) indicate putative gene conversion events identified by various computation methods. The color of the box corresponds to the computational method identifying each potential gene conversion, as indicated in the figure legend (A). The paralogs implicated in gene conversion are indicated within (or adjacent to) the colored box using the paralog suffix (e.g a gene conversion between human ADH1A and 1B is indicated by “A:B”). Homoplasic micro-indels in the intronic sequences are shown as vertical black arrows with the paralogs sharing these micro-indels indicated above each each arrow. Boundaries between introns or exons are demarcated with dotted vertical lines. Green boxes below the similarity plots indicate large gaps in the alignment, with the affected paralog indicated within the box. (TIF) [file pone.0041175.s008.tif]

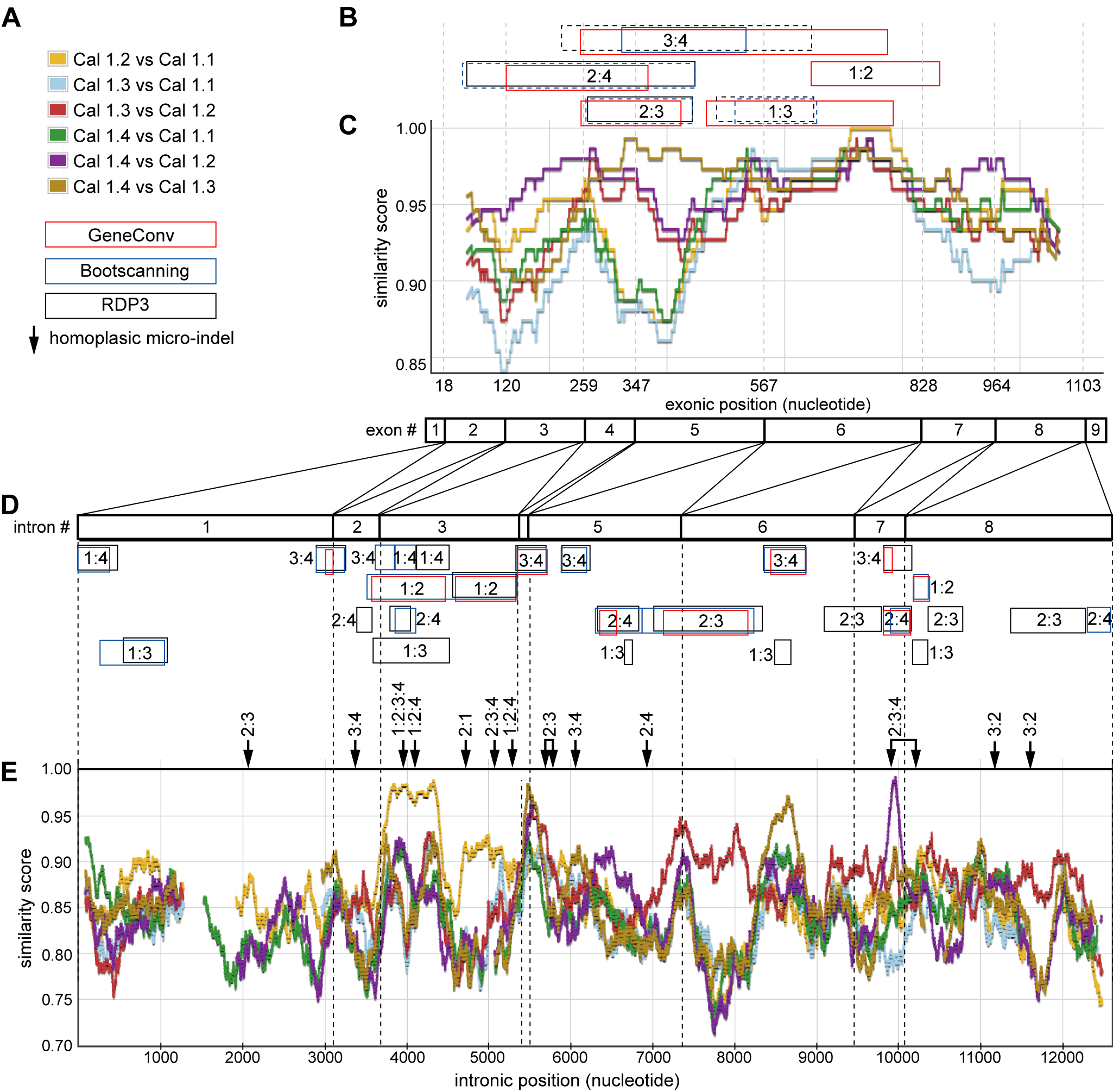

Supplement: Figure S9 — Summary of gene conversion analysis for marmoset ADH1 paralogs. Exonic and intronic data sets were examined for indicators of gene conversion using similarity plots, homoplasic micro-indels, and various computational methods. (A) The figure legend displays the color schemes used in subsequent panels for illustrating pairwise similarity scores among paralogs, and the key used to summarize the results from various methods used to identify potential gene conversions. The names of ADH1 paralogs have been shortened (e.g. the marmoset (Callithrix jacchus) ADH1 paralog “Cal_ADH1.1” is simply referred to as “Cal 1.1”). Pairwise similarity within a sliding window is plotted for various paralogs within (C) exonic regions and (E) intronic regions. The color of the line in the similarity plot corresponds to the identity of the paralog pair, as indicated in the figure legend (A). Similarity scores for exonic regions are calculated within a 150-nt sliding window, while that of intronic regions are calculated using a 250-nt sliding window. Colored boxes in (B) and (D) indicate putative gene conversion events identified by various computation methods. The color of the box corresponds to the computational method identifying each potential gene conversion, as indicated in the figure legend (A). Boxes with dashed borders indicate gene conversions that were not statistically significant at p-values <0.05, but were identified using p-values <0.10. The paralogs implicated in gene conversion are indicated within (or adjacent to) the colored box using the paralog suffix (e.g a gene conversion between Cal_ADH1.1 and Cal_ADH1.2 is indicated by “1∶2”). Homoplasic micro-indels in the intronic sequences are shown as vertical black arrows with the paralogs sharing these micro-indels indicated above each each. Boundaries between introns or exons are demarcated with dotted vertical lines. Green boxes below the similarity plots indicate large gaps in the alignment, with the affected paralog indicat [file pone.0041175.s009.tif]

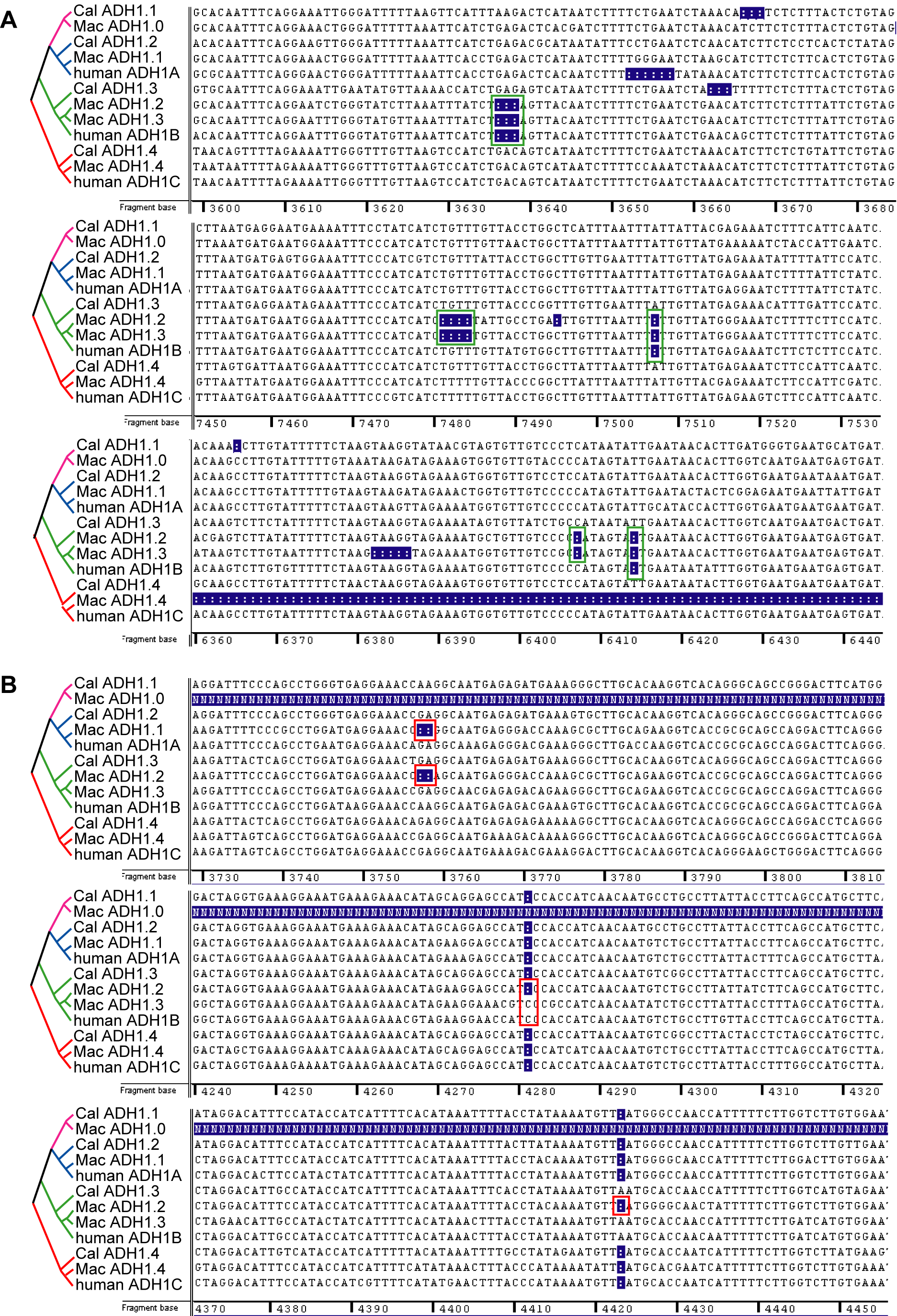

Supplement: Figure S10 — Examples of micro-indels within intronic alignments. Six sections from the multiple sequence alignment of the concatenated intronic data set are shown. Numbers below the alignment (“fragment base”) refer to the nucleotide position within the entire alignment. Gaps within individual sequences (denoted with “:” and highlighted blue) create indels. When the same indel occurred in two or more sequences, these shared micro-indels were scored as either “homoplasic” or “consistent” relative to the phylogenetic tree deduced from the entire dataset (as in Figure 2B, and shown to the left of each alignment subsection). (A) Examples of shared micoindels that are consistent with the evolutionary model deduced from the entire intronic dataset are indicated within a green box. (B) Examples of shared micoindels that are homoplastic with regard to the evolutionary model deduced from the entire intronic dataset are indicated within a red box. (TIF) [file pone.0041175.s010.tif]

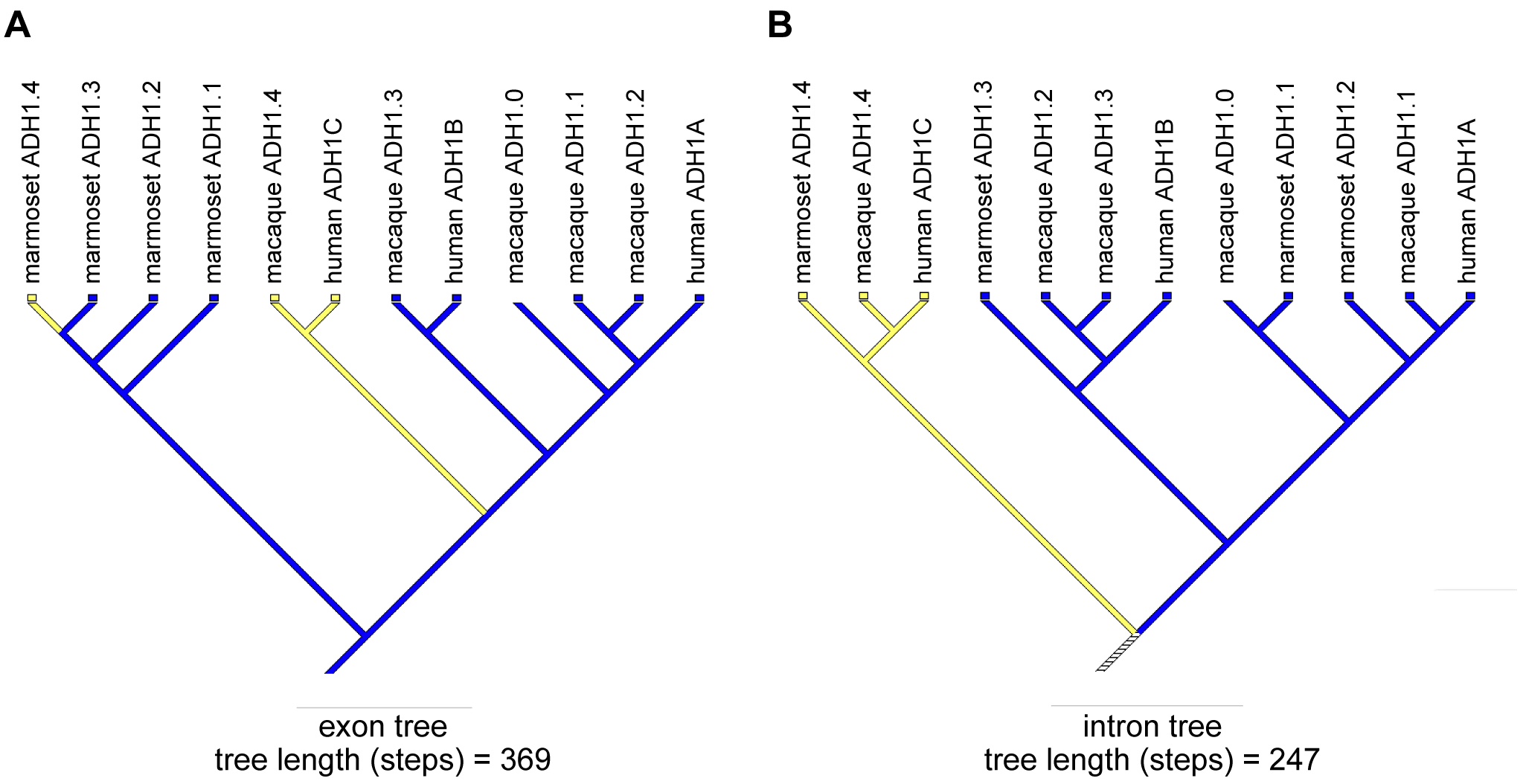

Supplement: Figure S12 — Parsimony analysis of the micro-indels coded as binary data. Micro-indels were coded as binary data, and parsimony analysis was conducted using MacClade to model the dataset according to either (A) the exon tree in Figure 2A, or (B) the intron tree in Figure 2B. The number of steps required to obtain the dataset when evolving according either model is shown below each tree. If the exon tree is modified by relocating Mac_ADH1.2 sister to Mac_ADH1.3 (as in the intronic tree), the parsimony score drops from 369 to 320 (tree not shown). This emphasizes the impact this single gene, and its associated gene conversion (see text) has on the overall parsimony score. The names of ADH1 paralogs have been shortened (e.g. the marmoset (Callthrix jacchus) ADH1 paralog “Cal_ADH1.1” is simply referred to as “marmoset ADH1.1I”). (TIF) [file pone.0041175.s012.tif]

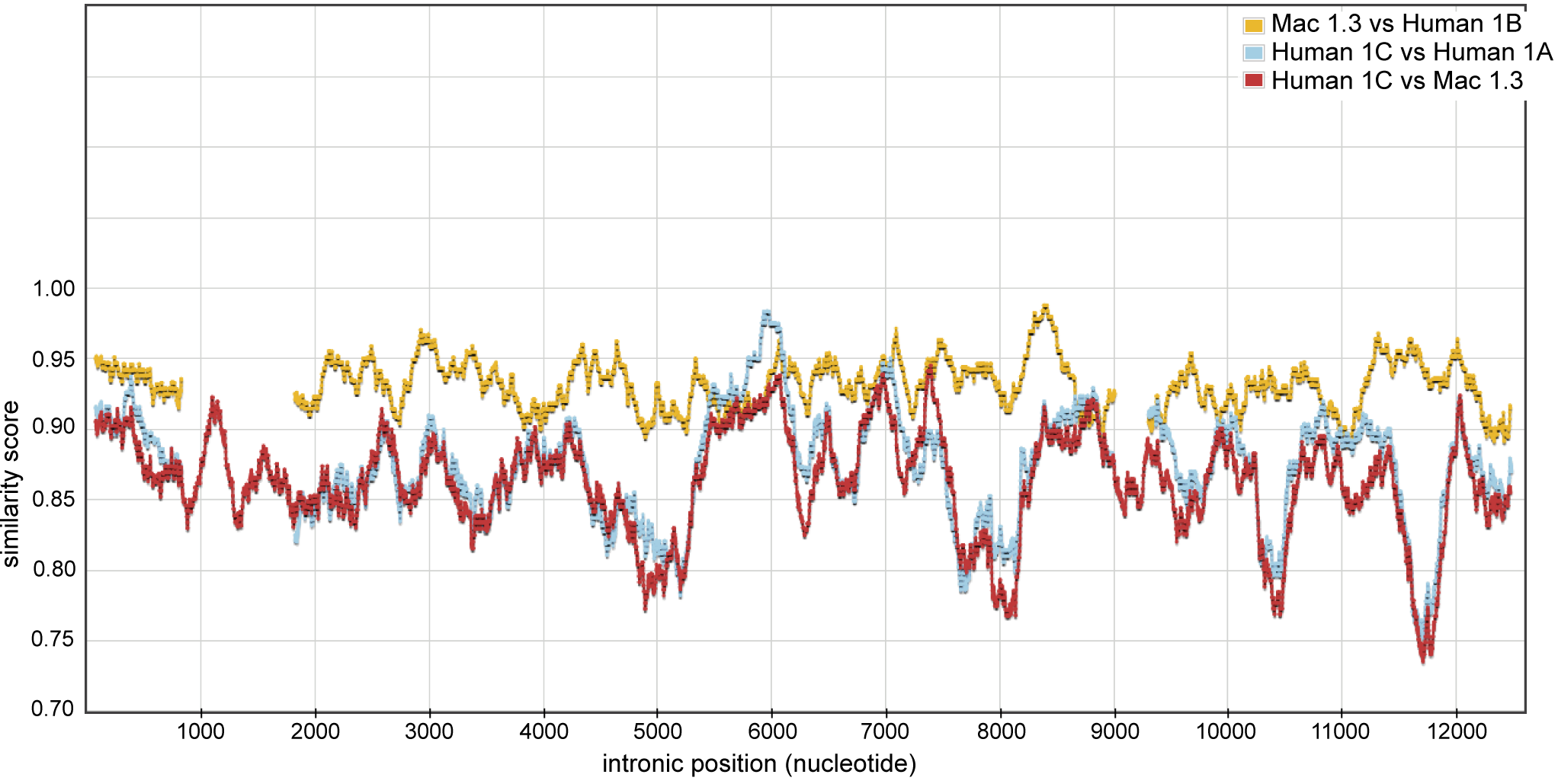

Supplement: Figure S13 — Similarity plot comparison of human ADH1B and its and macaque ortholog. Pairwise similarity is shown among the intronic regions of the human ADH1B, human ADH1C, and macaque Mac_ADH1.3 paralogs (window size = 250, minimum non-gapped positions = 200); the average of all pairwise distances across the entire alignment for this orthologous pair (human ADH1B and macaque Mac_ADH1.3) is 0.932+/−0.0174. The spike in pairwise similarity between human ADH1B and ADH1C at position 5900 and 6100 is presumably from a gene conversion (see text). The spike in pairwise similarity between human ADH1B and Mac_ADH1.3 at 8200–8500 likely reflects a gene conversion where Mac_ADH1.3 was converted to Mac_ADH1.4. (TIF) [file pone.0041175.s013.tif]

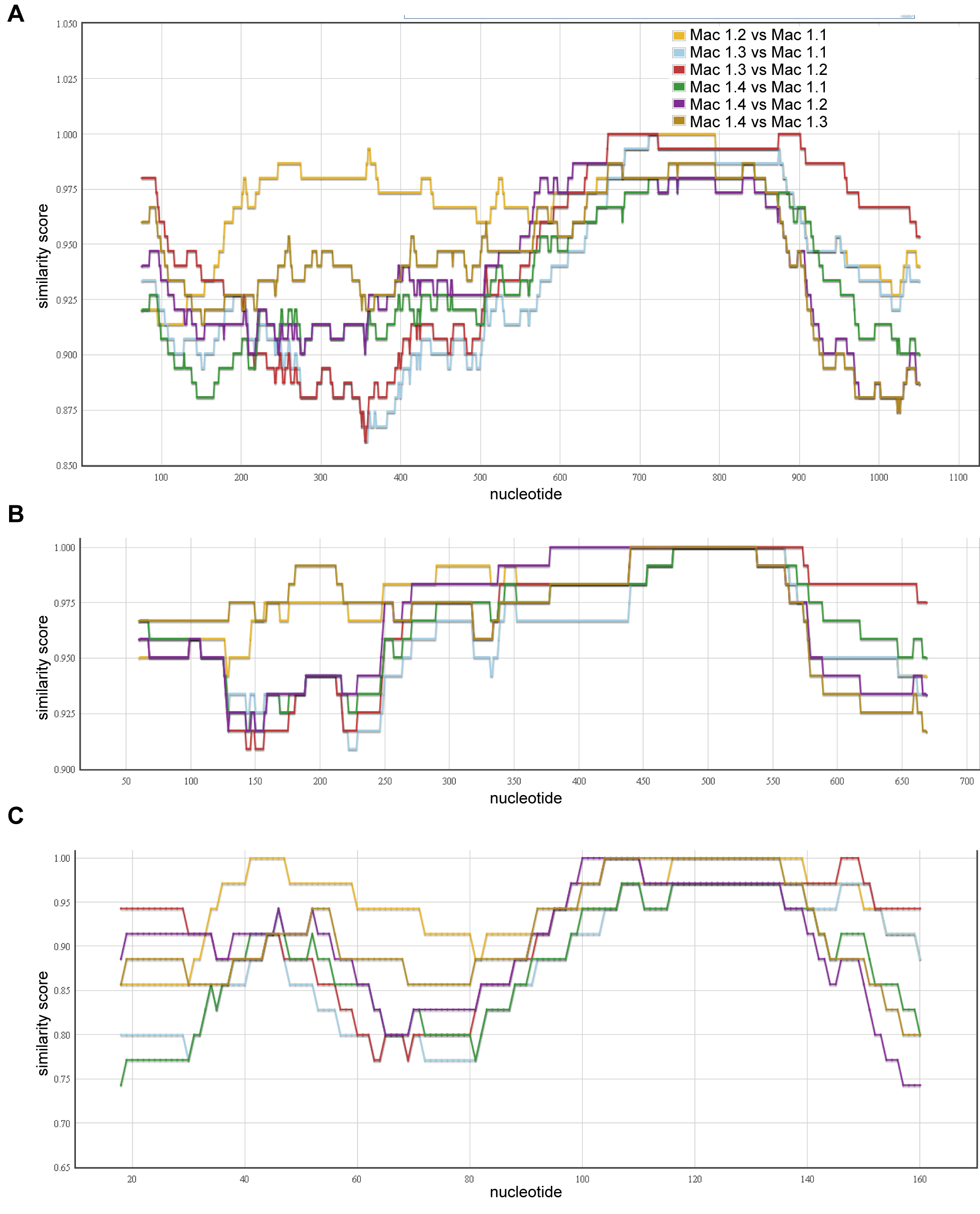

Supplement: Figure S14 — Similarity plot of macaque ADH1 exonic sequences partitioned by coding degeneracy. Pairwise similarity between each macaque paralog is shown using (A) all sites (window size = 150), (B) only 0-fold degenerate sites (window size = 120 nt), and (C) using only 4-fold degenerate sites (window size = 35-nt). (TIF) [file pone.0041175.s014.tif]

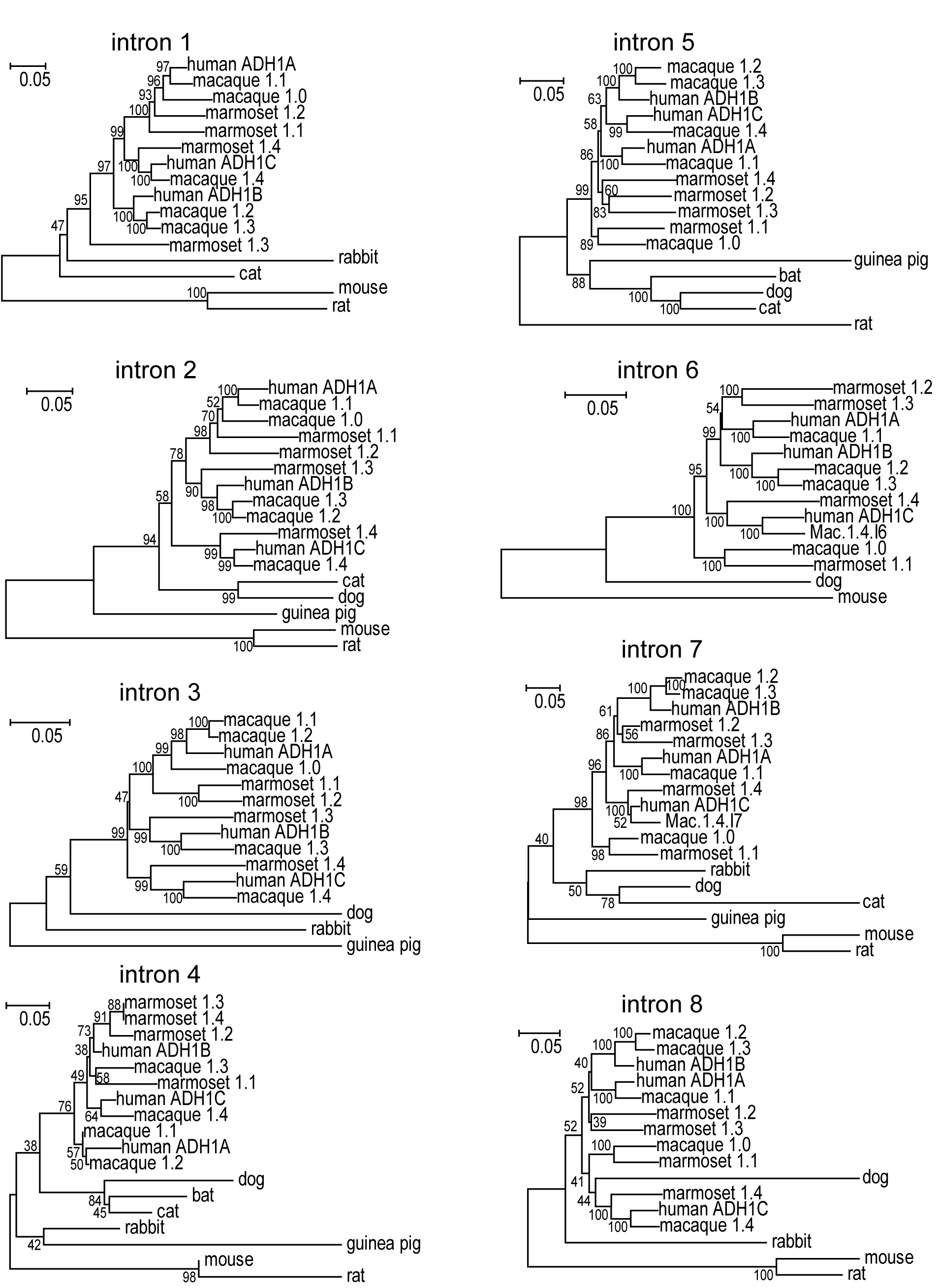

Supplement: Figure S15 — Phylogenetic analysis of individual ADH1 introns with non-primate outgroups. Neighbor-joining was used to determine the phylogeny of each intron individually after aligning with non-primate outgroups. The names of ADH1 paralogs have been shortened (e.g. the marmoset (Callthrix jacchus) ADH1 paralog “Cal_ADH1.1” is simply referred to as “marmoset ADH1.1”). Numbers at nodes refer to the bootstrap support values. (TIF) [file pone.0041175.s015.tif]

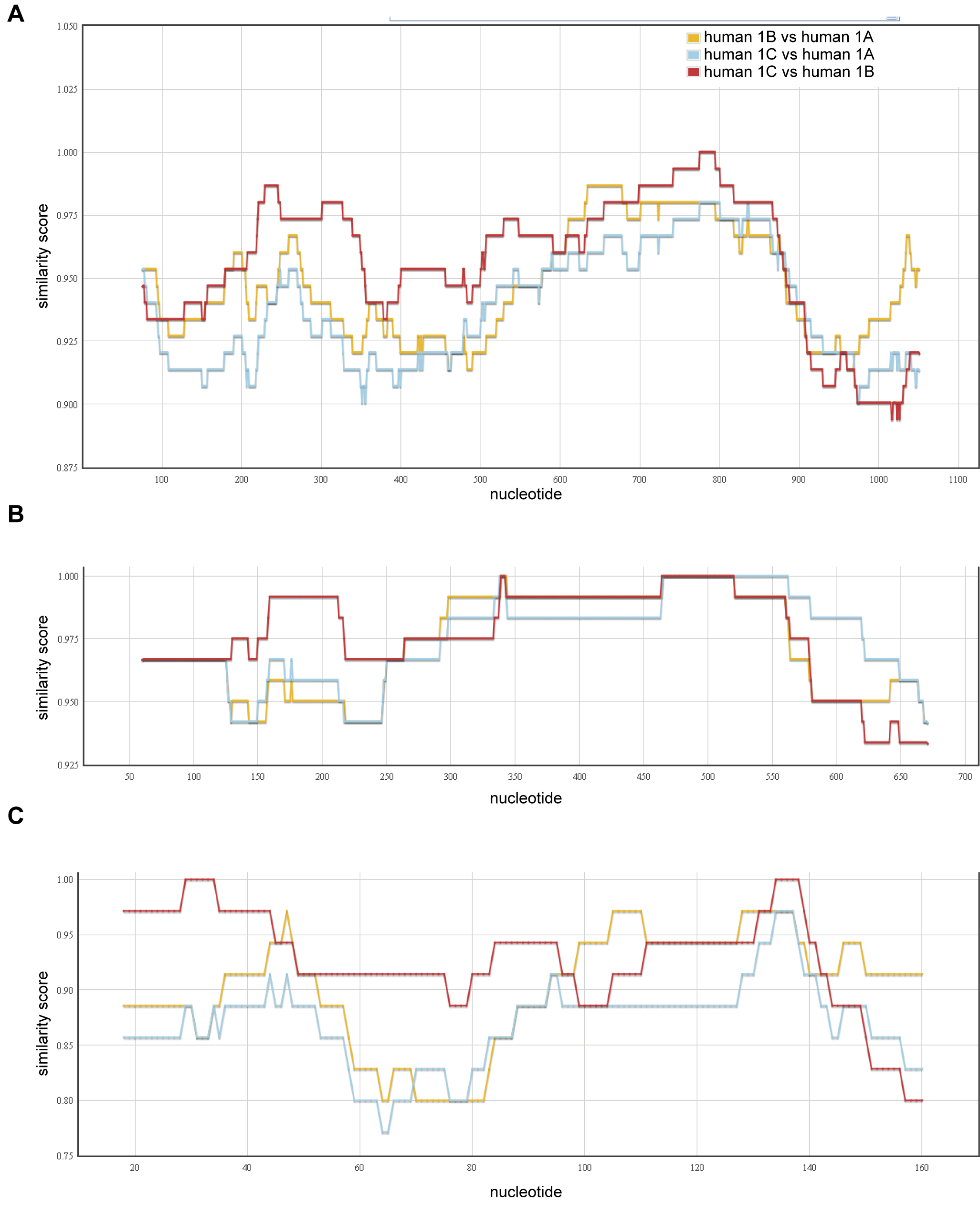

Supplement: Figure S16 — Similarity plot of human ADH1 exonic sequences partitioned by coding degeneracy. Pairwise similarity is shown between all human ADH1 paralogs using (A) all sites (window size 150), (B) only 0-fold degenerate sites (window size = 120 nt), and (C) using only 4-fold degenerate sites (window size = 35-nt). (TIF) [file pone.0041175.s016.tif]

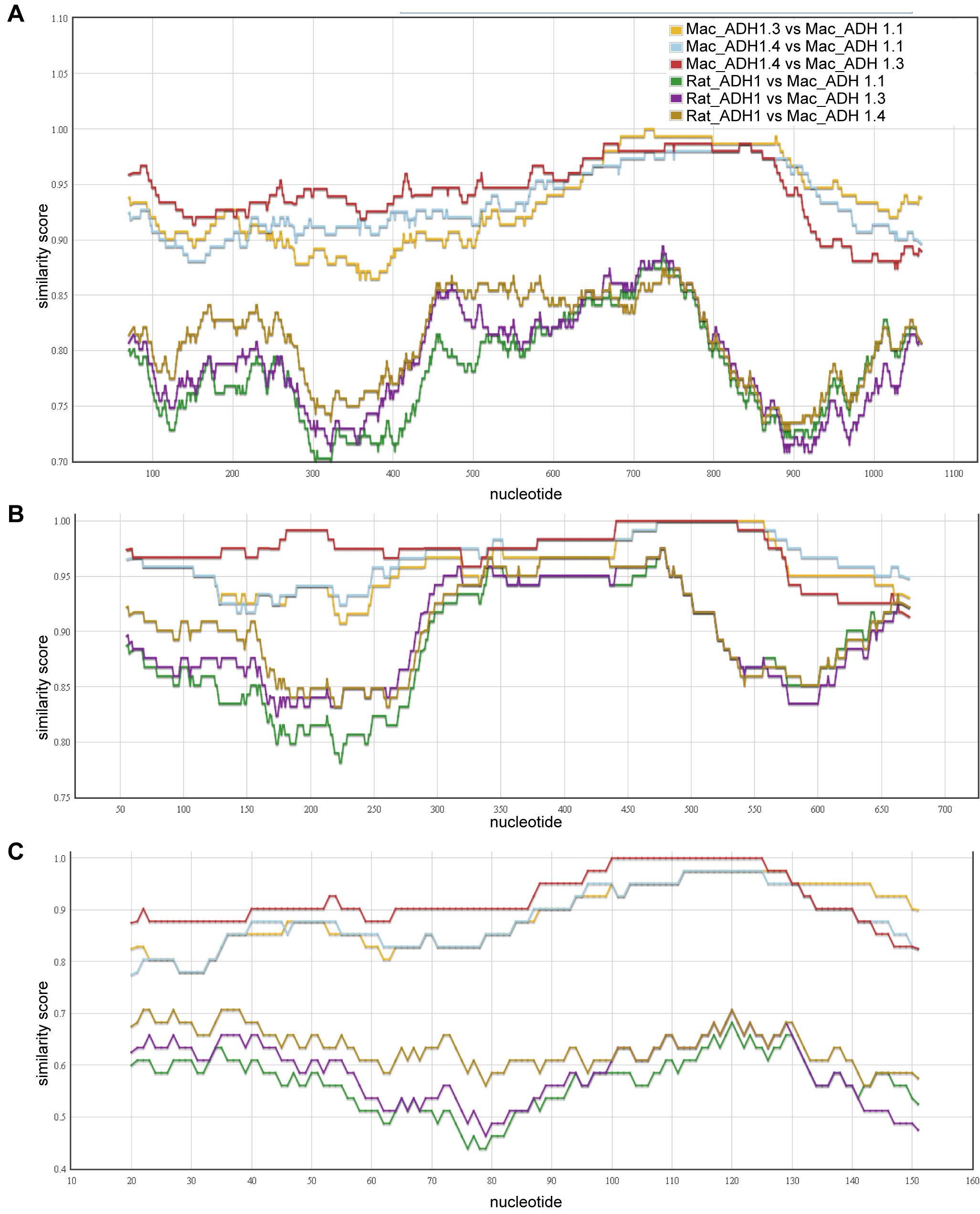

Supplement: Figure S17 — Similarity plot comparing exonic sequences (partitioned by coding degeneracy) of macaque ADH1 paralogs to their rat ortholog. Pairwise similarity between rat and macaque ADH1 paralogs is shown using (A) all sites (window size = 150), (B) only 0-fold degenerate sites (window size = 120 nt), and (C) using only 4-fold degenerate sites (window size = 35-nt). (TIF) [file pone.0041175.s017.tif]
