# Supplementary material for: The Natural History of Class I Primate Alcohol Dehydrogenases Includes Gene Duplication, Gene Loss, and Gene Conversion
Source: PLoS One. 2012 Jul 31;7(7):e41175. doi: 10.1371/journal.pone.0041175 (PMC3409193; doi:10.1371/journal.pone.0041175)
Supplement: Table S1 — Accession numbers for ADH genes used in this study. (DOC) [file pone.0041175.s018.doc]

**Table S1. Accession numbers for *ADH* genes used in this study**

| Ora.ADH.1.1_Pongo_abelii_ | ENSPPYP00000016692/ENSPPYG00000014949 Alcohol dehydrogenase 1A (EC 1.1.1.1) (Alcohol dehydrogenase subunit alpha). [Source:UniProtKB/Swiss-Prot;Acc:Q5RBP7] |
| --- | --- |
| Ora.ADH.1.2_Pongo_abelii_ | ENSPPYP00000016691/ENSPPYG00000014949 Alcohol dehydrogenase 1A (EC 1.1.1.1) (Alcohol dehydrogenase subunit alpha). [Source:UniProtKB/Swiss-Prot;Acc:Q5RBP7] |
| MacM_ADH1.0_Macaca_mulatta_ | Jan.2006 (MBSC Merged1.0/rheMax2) assembly of UCSC Genome Browser, position: chromosome 5: 92,206,705-92,222,014; the missing section near exon 4 was filled in with a BAC read found in the NCBI database, gb|AC210715.3|. |
| MacM.ADH.1.1_Macaca_mulatta_ | ENSMMUP00000034974/ENSMMUG00000018738 Alcohol dehydrogenase 1A (EC 1.1.1.1) (Alcohol dehydrogenase alpha subunit). [Source:UniProtKB/Swiss-Prot;Acc:P28469] chromosome 5:92,237,901-92,252,655 |
| MacM.ADH.1.2_Macaca_mulatta_ | ENSMMUP00000034970/ENSMMUG00000018738 Alcohol dehydrogenase 1A (EC 1.1.1.1) (Alcohol dehydrogenase alpha subunit). Source:UniProtKB/Swiss-Prot;Acc:P28469] Same as: NCBI Reference Sequence: NM_001042765.1 Macaca mulatta alcohol dehydrogenase 1A (class I), alpha polypeptide (ADH1A), mRNA; chromosome 5: 92,268,575-92,286,411 |
| MacM.ADH.1.3_Macaca_mulatta_ | ENSMMUP00000034968/ENSMMUG00000018738 Alcohol dehydrogenase 1A (EC 1.1.1.1) (Alcohol dehydrogenase alpha subunit). Source:UniProtKB/Swiss-Prot;Acc:P28469] Same as: NCBI Reference Sequence: XM_001106834.1 PREDICTED: Macaca mulatta alcohol dehydrogenase 1B (class I), beta polypeptide, transcript variant 3 (ADH1B), mRNA; chromosome 5: 92,294,687-92,309,207 |
| MacM.ADH.1.4_Macaca_mulatta_ | ENSMMUP00000021006/ENSMMUG00000018738 Alcohol dehydrogenase 1A (EC 1.1.1.1) (Alcohol dehydrogenase alpha subunit). Source:UniProtKB/Swiss-Prot;Acc:P28469] Same as: NCBI Reference Sequence: XM_001099784.1 PREDICTED: Macaca mulatta class I alcohol dehydrogenase, gamma subunit (ADH1C), mRNA; chromosome 5: 92,319,350-92,345,251 |
| Bab.ADH.1.2_Papio_hamadryas_ | GenBank: L30113.1 Papio hamadryas alcohol dehydrogenase class I (ADH) mRNA, complete cds |
| Boar.ADH.1.1_Sus_scrofa_ | GenBank: AK233380.1 Sus scrofa mRNA, clone:LVRM10135H10, expressed in liver |
| Cow.ADH.1.1_Bos_taurus_ | NCBI Reference Sequence: XM_586772.4 PREDICTED: Bos taurus alcohol dehydrogenase 1C (class I), gamma polypeptide, transcript variant 1 (ADH1C), mRNA |
| Dog.ADH.1.1_Canis_familiaris_ | NCBI Reference Sequence: XM_535667.2 PREDICTED: Canis familiaris similar to Alcohol dehydrogenase gamma chain (LOC478489), mRNA |
| Goph.ADH.1.1_Geomys_bursarius_ | GenBank: AF044728.1 Geomys bursarius major alcohol dehydrogenase (ADH1) mRNA, allele M''', complete cds |
| Hors.ADH.1.1_Equus_caballus_ | NCBI Reference Sequence: NM_001081945.1 Equus caballus alcohol dehydrogenase-S-isoenzyme (LOC100034175), mRNA |
| Homo.ADH.1.a_Homo_sapiens_ | NCBI Reference Sequence:gi_11496886_ref_NM_000667.2_ Homo sapiens alcohol dehydrogenase 1A (class I), alpha polypeptide (ADH1A), mRNA |
| Homo.ADH.1.b_Homo_sapiens_ | NCBI Reference Sequencegi_160298141_ref_NM_000668.4_ Homo sapiens alcohol dehydrogenase 1B (class I), beta polypeptide (ADH1B), mRNA |
| Homo.ADH.1.c_Homo_sapiens_ | NCBI Reference Sequence:gi_71565150_ref_NM_000669.3_ Homo sapiens alcohol dehydrogenase 1C (class I), gamma polypeptide (ADH1C), mRNA |
| MacF.ADH.1.a_Macaca_fascicularis_ | GenBank: AK240628.1 Macaca fascicularis mRNA, complete cds, clone Qlv-U339A-H12: similar to Homo sapiens alcohol dehydrogenase 1A (class I), alpha polypeptide (ADH1A), mRNA, NM_000667.2 |
| MacF.ADH.1.b_Macaca_fascicularis_ | GenBank: AK240629.1 Macaca fascicularis mRNA, complete cds, clone Qlv-U343A-A10: similar to Homo sapiens alcohol dehydrogenase IB (class I), beta polypeptide (ADH1B), mRNA, NM_000668.3 |
| MusC.ADH.1.1_Mus_caroli_ | GenBank: M11307.1 Mouse class I alcohol dehydrogenase (ADH-AA) mRNA, complete cds |
| MusM.ADH.1.1_Mus_musculus_ | GenBank: BC054467.1 |
| Ora.ADH .1.a_Pongo_abelii_ | NCBI Reference Sequence: NM_001131990.1 Pongo abelii alcohol dehydrogenase 1A (class I), alpha polypeptide (ADH1A), mRNA |
| Pan.ADH.1.a_Patroglodytes_ | NCBI Reference Sequence: XM_001166683.1 PREDICTED: Pan troglodytes class I alcohol dehydrogenase, alpha subunit (ADH1A), mRNA |
| Pan.ADH.1.b_Patroglodytes_ | NCBI Reference Sequence: NM_001034158.1 Pan troglodytes alcohol dehydrogenase 1B (class I), beta polypeptide (ADH1B), mRNA |
| Pan.ADH.1.c_Patroglodytes_ | NCBI Reference Sequence: XM_001167058.1 PREDICTED: Pan troglodytes class I alcohol dehydrogenase, gamma subunit, transcript variant 4 (ADH1C), mRNA |
| Rat.ADH.1.1_Rattus_norvegicus_ | NCBI Reference Sequence: NM_019286.3 Rattus norvegicus alcohol dehydrogenase 1 (class I) (Adh1), mRNA |
| Mar.ADH.1.1_Callithrix_jacchus_ | UCSC Genome (Contig1120.001.a) Same as our sequencing- Mar1203.1.2; UCSC Genome Browser, March 2009 (WUGSC 3.2/calJac3) Assembly, chromosome 3, 94,428,469-94,442,948 |
| Mar.ADH.1.2_Callithrix_jacchus_ | UCSC Genome (Contig1120.001.a); UCSC Genome Browser, March 2009 (WUGSC 3.2/calJac3) Assembly, chromosome 3, 94,392,510-94,407,997 |
| Mar.ADH.1.3_Callithrix_jacchus_ | UCSC Genome (Contig1120.001.a) ; Position: Contig1120:75112-209500: same as our sequencing- Mar1203.1.1 ; UCSC Genome Browser, March 2009 (WUGSC 3.2/calJac3) Assembly, chromosome 3, 94,349,029-94,378,928 |
| Mar.ADH.1.4_Callithrix_jacchus_ | UCSC Genome (Contig1120.001.a) ; UCSC Genome Browser, March 2009 (WUGSC 3.2/calJac3) Assembly, chromosome 3, 94,307,535-94,332,187 |
| Ring-tailed lemur ADH1.w | New sequence from this work; GenBank accession number JX155347 |
| Ring-tailed lemur ADH1.x | New sequence from this work; GenBank accession number JX155348 |
| Ring-tailed lemur ADH1.y | New sequence from this work; GenBank accession number JX155349 |
| Ring-tailed lemur ADH1.z | New sequence from this work; GenBank accession number JX155350 |
| Brown lemur ADH1 | New sequence from this work; GenBank accession number JX155354 |
| Sifaka ADH1.t | New sequence from this work; GenBank accession number JX155351 |
| Sifaka ADH1.u | New sequence from this work; GenBank accession number JX155352 |
| Sifaka ADH1.v | New sequence from this work; GenBank accession number JX155353 |
| Tree shrew ADH1 | New sequence from this work; GenBank accession number JX155356 |
| Chick.ADH.1 | >ENSGALT00000020006 cdna:KNOWproteicoding |
| ADH1_CHICK_Chick.ADH.Z | >ENSGALT00000032122 cdna:KNOWproteicoding |
| Chick.ADH.Y | >ENSGALT00000020007 cdna:KNOWproteicoding |
| Chick.ADH.W | >ENSGALT00000032127 cdna:KNOWproteicoding |
| Danio.ADH.Q | >ENSDART00000042766 cdna:KNOWproteicoding |
| Danio.ADH.R | >ENSDART00000008498 cdna:KNOWproteicoding |
| Danio.ADH.S | >ENSDART00000108825 cdna:KNOWproteicoding |
| Danio.ADH.T | >ENSDART00000027892 cdna:KNOWproteicoding |
| Danio.ADH.U | >ENSDART00000105604 cdna:KNOWproteicoding |
| Frog.ADH.E | - ENSXETT00000020500 |
| Frog.ADH.F | - ENSXETT00000020510 |
| Frog.ADH.H | - NM_001030410.1 Xenopus (Silurana) tropicalis alcohol dehydrogenase 1C (class I), gamma polypeptide (adh1c), mRNA |
| Frog.ADH.D | - gi|58332695|ref|NM_001011423.1| Xenopus (Silurana) tropicalis alcohol dehydrogenase 6 (class V) (adh6), mRNA |
| Frog.ADH.J | - gi|58332633|ref|NM_001011391.1| Xenopus (Silurana) tropicalis alcohol dehydrogenase 1 (adh1), mRNA |
| Frog.ADH.K | - NM_001011431.1 Xenopus (Silurana) tropicalis alcohol dehydrogenase 1B (class I), beta polypeptide (adh1b), mRNA |
| Frog.ADH.L | - gi|58332851|ref|NM_001011502.1| Xenopus (Silurana) tropicalis alcohol dehydrogenase 5 (class III), chi polypeptide (adh5), mRNA |
| Homo.ADH.2_Homo_sapiens_ | - NCBI Reference Sequence: GenBank: AK290835.1 Homo sapiens cDNA FLJ77091 complete cds, highly similar to Homo sapiens alcohol dehydrogenase 4 (class II), pi polypeptide (ADH4), mRNA |
| Dog.ADH.2_Canis_familiaris_ | - NCBI Reference Sequence: XM_535665.2 PREDICTED: Canis familiaris similar to Alcohol dehydrogenase class II pi chain precursor, transcript variant 2 (LOC478487), mRNA  --- the NCBI dog sequence shows a deletion of amino acid 331 (Val), 991 (GTA), relative to human; our database has inserted 3 N’s into the NCBI dog sequence at this position to give proper alignment with human sequence. |
| Cow.ADH.2_Bos_taurus_ | - NCBI Reference Sequence: NCBI Reference Sequence: NM_001102059.1 Bos taurus alcohol dehydrogenase 4 (class II), pi polypeptide (ADH4), mRNA |
| Pig.ADH.2_Sus_scrofa_ – | ENSEMBLE Reference Sequence: Transcript: ENSSSCT00000010057 Alcohol dehydrogenase 4 (EC 1.1.1.1)(Alcohol dehydrogenase class II pi chain) [Source:UniProtKB/Swiss-Prot;Acc:P08319] Source: 1.1.1.1 Chromosome 8: 103,894,886-103,916,227 forward strand. This transcript is a product of gene ENSSSCG00000009184.  ---ENSEMBLE miscalled the first exon (6 AA, 18bps) relative to human. In order to correct the first exon we BLASTed the ENSEMBLE reference Pig.ADH.2 sequence against the Reference mRNA sequence (refseq_rna) database for Sus scrofa in NCBI. We picked mRNAs which covered the first miscalled exon with >99% identity to the Pig.ADH.2 reference sequence of more than 200bps downstream of the first exon.  The following mRNA was used to correct the first exon:  gi|221032078|gb|FD605122.1|FD605122 susfleck_LV_12_A09 SUSFLECK Liver Sus scrofa cDNA clone 12_A09, mRNA sequence |
| Mus.ADH.2_Mus_musculus_ | - NCBI Reference Sequence: GenBank: AJ245750.1 Mus musculus mRNA for alcohol dehydrogenase II (ADH2 gene) |
| Rab.ADH.2_Oryctolagus_cuniculus_ | - NCBI Reference Sequence: GenBank: AJ002388.1 Oryctolagus cuniculus mRNA for class II alcohol dehydrogenase, isozyme 1 |
| Homo.ADH.3_Homo_sapiens_ | - NCBI Reference Sequence: NM_000671.3 Homo sapiens alcohol dehydrogenase 5 (class III), chi polypeptide (ADH5), mRNA >gi|71565153|ref|NM_000671.3| Homo sapiens alcohol dehydrogenase 5 (class III), chi polypeptide (ADH5), mRNA |
| Cow.ADH.3_Bos_taurus_ | - NCBI Reference Sequence: NM_001034249.1 Bos taurus alcohol dehydrogenase 5 (class III), chi polypeptide (ADH5), mRNA |
| Dog.ADH.3_Canis_familiaris_ | - NCBI Reference Sequence: XM_532181.2| PREDICTED: Canis familiaris similar to Alcohol dehydrogenase class III chi chain (Glutathione-dependent formaldehyde dehydrogenase) (FDH), transcript variant 1 (LOC474946), mRNA |
| Mus.ADH.3_Mus_musculus_ | - NCBI Reference Sequence: GenBank: BC062879.1 Mus musculus alcohol dehydrogenase 5 (class III), chi polypeptide, mRNA (cDNA clone MGC:86026 IMAGE:5691327), complete cds |
| Pig.ADH.3_Sus_scrofa_ | - NCBI Reference Sequence: GenBank: AK235370.1 Sus scrofa mRNA, clone:OVRM10074B12, expressed in ovary |
| Rat.ADH.3_Rattus_norvegicus_ | - NCBI Reference Sequence: NM_001126120.1 Rattus norvegicus alcohol dehydrogenase 5 (Adh5), mRNA |
| Homo.ADH.4_Homo_sapiens_ | - NCBI Reference sequence: gi|71743839|ref|NM_000673.3| Homo sapiens alcohol dehydrogenase 7 (class IV), mu or sigma polypeptide (ADH7), mRNA |
| Opo.ADH.4_Monodelphis_domestica_ | - NCBI Reference Gene: XM_001369736.1 (ENSMODG00000020710) Location Chromosome 5: 51,644,026-51,660,712 forward strand. ---This sequence is missing data for the first exon. The gap in the first exon was replaced with N’s to facilitate alignment. |
| Mus.ADH.4_Mus_musculus_ | - NCBI Reference Sequence: NM_009626.4| Mus musculus alcohol dehydrogenase 7 (class IV), mu or sigma polypeptide (Adh7), mRNA |
| Rat.ADH.4_Rattus_norvegicus_ | - NCBI Reference Sequence: NM_134329.1| Rattus norvegicus alcohol dehydrogenase 7 (class IV), mu or sigma polypeptide (Adh7), mRNA |
| Tars.ADH.4_Tarsius_syrichta_ | - ENSEMBLE Reference Sequence: Transcript: ENSTSYT00000004615 Alcohol dehydrogenase class 4 mu/sigma chain (EC 1.1.1.1)(Alcohol dehydrogenase class IV mu/sigma chain)(Retinol dehydrogenase)(Gastric alcohol dehydrogenase) [Source:UniProtKB/Swiss-Prot;Acc:P40394] Source: 1.1.1.1  Reference Tarsier sequence has a miscalled 3’ last exon relative to NCBI Human. This error is the same as with the reference ENSEMBLE Gorilla and Orangutan sequence (see above for explanation and images). Searched all databases NCBI has, no sequence for the 3’ end of Tarsier ADH4 is present in any of them. |
| Tree.ADH.4_Tupaia_belangeri_ | - ENSEMBLE Reference Sequence: Transcript: ENSTBET00000013224 Alcohol dehydrogenase class 4 mu/sigma chain (EC 1.1.1.1)(Alcohol dehydrogenase class IV mu/sigma chain)(Retinol dehydrogenase)(Gastric alcohol dehydrogenase) [Source:UniProtKB/Swiss-Prot;Acc:P40394] Source: 1.1.1.1  The Reference Tree includes called exons with only 2-3 bases, based on genomic data. These errors are in the third exon (when compared to reference NCBI human); (picture available upon request).- We BLASTed the reference NCBI Human Exon 3 against the trace whole shotgun sequencing database in NCBI, for Tupaia belangeri. Two traces matched >90% against the human ADH4, and matched the exon/intron boundries in the reference human exon 3.  We corrected the miscalled exon 3 in Tupaia using the following traces: G835P69025FK21.T0, G835P65870FE17.T0 |
| Homo.ADH.5_Homo_sapiens_ | - NCBI Reference Sequence: NM_001102470.1 Homo sapiens alcohol dehydrogenase 6 (class V) (ADH6), transcript variant 1, mRNA |
| Tree.ADH.5_Tupaia_belangeri_ | - Transcript: ENSTBET00000015786 alcohol dehydrogenase 6 (class V) [Source:HGNC Symbol;Acc:255] GeneScaffold_3252: 258,728-271,688 reverse strand. This transcript is a product of gene ENSTBEG00000015792 |
| Cow.ADH.5_Bos_taurus_ | - NCBI Reference Sequence: NM_001046057.1 Bos taurus alcohol dehydrogenase 6 (class V) (ADH6), mRNA |
