# Supplementary material for: The Natural History of Class I Primate Alcohol Dehydrogenases Includes Gene Duplication, Gene Loss, and Gene Conversion
Source: PLoS One. 2012 Jul 31;7(7):e41175. doi: 10.1371/journal.pone.0041175 (PMC3409193; doi:10.1371/journal.pone.0041175)
Supplement: Table S2 — Source of tissues used in this study. (DOC) [file pone.0041175.s019.doc]

**Table S2. Source of tissues used in this study**

| **Common Name** | **Genus/Species** | **Tissue** | **Organization** | **Approving Committee** | **contact** |
| --- | --- | --- | --- | --- | --- |
| Sifaka | *Propithecus coquereli* | Liver | Duke Lemur Center | Duke Lemur Center Research Committee | Sarah Zehr |
| Brown Lemur | *Eulemur fulvus collaris* | Liver | Duke Lemur Center | Ibid. | Ibid. |
| Ring-tailed Lemur | *Lemur catta* | Liver | Duke Lemur Center | Ibid. | Ibid. |
| Northern Tree Shrew | *Tupaia belangeri* | Liver | University of Alabama, Birmingham; Department of Vision Sciences | Institutional Animal Care and Use Committee of the University of Alabama at Birmingham | Thomas Norton |
| Northern Tree Shrew | *Tupaia belangeri* | Liver | Duke Institute for Brain Sciences | Duke University Animal Care and Use Committee | David Fitzpatrick |
| Common Tree Shrew | *Tupaia glis* | Liver | Bronx Zoo | Wildlife Conservation Society Biomaterials Committee and the Wildlife Conservation Society Animal Management Committee | Lisa Harley Newton |
| Marmoset | *Callithrix jacchus* | Liver | Wisconsin National Primate Research Center, Grant Number RR000167 | Institutional Animal Care and Use Committee of the University of Wisconsin | Jordana Lenon |
| Marmoset | *Callithrix jacchus* | Fetal liver | Southwest National Primate Research Center, funded by the National Center for Research Resources [p51RR013986] and the Office of Research Infrastructure Programs/OD [P51OD011107] | Institutional Animal Care and Use Committee of the Southwest National Primate Research Center | Jera Pecotte |
