# Supplementary material for: The Natural History of Class I Primate Alcohol Dehydrogenases Includes Gene Duplication, Gene Loss, and Gene Conversion
Source: PLoS One. 2012 Jul 31;7(7):e41175. doi: 10.1371/journal.pone.0041175 (PMC3409193; doi:10.1371/journal.pone.0041175)
Supplement: Table S3 — Primers used in this study. (DOC) [file pone.0041175.s020.doc]

**Table S3. Primers used in this study.**

| General purpose primers used for amplifying primate and tree shrew ADH1 sequences. | | | | | | |
| --- | --- | --- | --- | --- | --- | --- |
| **direction** | **Primer #** | **Primer Name** | **estimated Tm (˚C)** | **annealing temp used in PCR (˚C)** | **Length** | **Sequence (5' to 3')** |
| Forward | 4217-0243 | adh1For_v1 | 62.0 | 58 | 28 | agtctcctggtctgcagagaagacagaa |
| Forward | 4239-7343 | adh1For_v3 | 59.2 | 58 | 26 | cagggaagaaatccacaaggactcac |
| Forward | 4434-3551 | adh1For_v8 | 60.0 | 58 | 24 | tctcctggtctgcasagaagacag |
| Reverse | 4217-0245 | adh1Rev_v1 | 64.0 | 58 | 31 | tgtagggtagaggaggctgaagactgctaca |
| Reverse | 4239-7344 | adh1Rev_v3 | 58.1 | 58 | 25 | ctgctacaagggaaggcatctctat |
| Primers designed to be specific to Cal_ADH1.2 | | | |  |  |  |
| **direction** | **Primer #** | **Primer Name** | **estimated Tm (˚C)** | **annealing temp used in PCR (˚C)** | **Length** | **Sequence (5' to 3')** |
| Forward | 5005-2413 | adh1For_v30 | 61.5 | 58 | 27 | atgagcacggcaggaaaagtaagcaac |
| Forward | 5005-2414 | adh1For_v31 | 64.4 | 58 | 34 | ctgcttgaaaaacgatctcagcaatgctcaggga |
| Forward | 5005-2415 | adh1For_v32 | 65.7 | 58 | 31 | aggggttggcctttctgtcgttatgggatgt |
| Reverse | 5005-2416 | adh1Rev_v30 | 61.7 | 58 | 34 | aaacatcaggacagtacggatacttttcccagaa |
| Reverse | 5005-2417 | adh1Rev_v31 | 59.5 | 58 | 36 | ggtaaaacgttggttattaatgcatccagtgaaaat |
| Primers designed to be specific to Cal_ADH1.4 | | | |  |  |  |
| **direction** | **Primer #** | **Primer Name** | **estimated Tm (˚C)** | **annealing temp used in PCR (˚C)** | **Length** | **Sequence (5' to 3')** |
| Forward | 5005-2418 | adh1For_v33 | 69.5 | 58 | 36 | tggaagtggcacctcctaaggcccatgaagttcgca |
| Forward | 5005-2419 | adh1For_v34 | 62.7 | 58 | 32 | gcattaagatggtggctgtaggaatctgtcgt |
|  |  |  |  |  |  |  |
| Reverse | 5005-2420 | adh1Rev_v33 | 56.7 | 55 | 29 | gtaaagcattggttattaatgcatccagg |
| Reverse | 5005-2421 | adh1Rev_v34 | 60.6 | 58 | 36 | ggggacatcttctttactcttatagcctccaaaaat |

Multiple primers were designed based on different regions in the 5’- and 3’-UTR, but targeted toward regions conserved among all available primate ADH1 genes. Separate reverse transcription (RT) reactions were performed using each of the various reverse primers. Each of these RTs was then used as a template for multiple PCR reactions using the same reverse primer used in the RT, but individually varying the forward primers. PCRs were then analyzed by agarose gel electrophoresis to identify primer combinations yielding successful amplification. In order to maximize the diversity of paralogs isolated, every successful primer combination was cloned and sequenced. PCRs conditions were: 95˚ for 4 minutes; (95˚for 30 seconds, anneal at various temperatures for 45 seconds, 72˚ for 2 minutes) x 45 cycles.
