# Supplementary material for: The Natural History of Class I Primate Alcohol Dehydrogenases Includes Gene Duplication, Gene Loss, and Gene Conversion
Source: PLoS One. 2012 Jul 31;7(7):e41175. doi: 10.1371/journal.pone.0041175 (PMC3409193; doi:10.1371/journal.pone.0041175)
Supplement: Table S4 — Summary of sequences retrieved from marmoset SRA and macaque EST databases. (DOC) [file pone.0041175.s021.doc]

**Table S4. Summary of sequences retrieved from marmoset SRA and macaque EST databases.**

| gene | # of hits found | Tissue source | Example sequence |
| --- | --- | --- | --- |
| Cal_ADH1.1 | 9 | kidney (adult) | SRA:SRR000079.87562.2 |
| Cal_ADH1.2 | none |  |  |
| Cal_ADH1.3 | 23 | kidney (adult) | SRA:SRR000078.124728.2 |
| Cal_ADH1.4 | none |  |  |
| Mac_ADH1.1 | none |  |  |
| Mac_ADH1.2 | 8 | Liver | BB890805 Sugano cDNA library, adult liver Macaca fascicularis cDNA clone Qlv-U401A-F8 3', mRNA sequence. |
| Mac_ADH1.3 | 1 | Pancreas | FS367772 macaque pancreas cDNA library QpaA Macaca fascicularis cDNA clone QpaA-14356 5', mRNA sequence. |
|  | 51 | Liver | DC627816 macaque liver cDNA library QlvC Macaca fascicularis cDNA clone QlvC-25149 5 |
|  | 1 | Thymus | DK579306 macaque thymus cDNA library QthA Macaca fascicularis cDNA clone QthA-12315 5', mRNA sequence |
|  | 1 | heart | FS583267 macaque heart cDNA library QhtB Macaca fascicularis cDNA clone QhtB-23311 5', mRNA sequence. |
| Mac_ADH1.4 | 1 | Liver | GenBank: CO775503.1 ILLUMIGEMCQ_52813 KatzMFLV (liver) Macaca fascicularis cDNA clone IBIUW:28567 5- similar to Bases 7 to 960 highly similar to human ADH1C (Hs.2523), mRNA sequence |
|  | 1 | Pancreas | FS369196 macaque pancreas cDNA library QpaA Macaca fascicularis cDNA clone QpaA-1 |
|  | 9 | Kidney | DC648039 macaque kidney cDNA library QreB Macaca fascicularis cDNA clone QreB-06252 5', mRNA sequence |
|  | 1 | ovary | gb|DY759790.1| 172360 Pigtailed macaque ovary library Macaca nemestrina cDNA 5', mRNA sequence. |
|  | 4 | jejunum | GenBank: CO582804.1 ILLUMIGEMCQ_43997 KatzMMJJ (jejunum) Macaca mulatta cDNA clone IBIUW:20751 5- similar to Bases 21 to 931 highly similar to human ADH1C (Hs.2523), mRNA sequence |
|  | 2 | ileum | GenBank: CO579288.1 ILLUMIGEMCQ_50376 KatzMMIL (ileum) Macaca mulatta cDNA clone IBIUW:17007 5- similar to Bases 5 to 947 highly similar to human ADH1C (Hs.2523), mRNA sequence |

Macaque EST databases contain mRNA isolated primarily (but not exclusively) from spleen mononuclear lymphocyte, brain, lung, two placental libraries (from male and female fetuses), liver, duodenum, ileum, jejunum, testes, ovary, and activated PBMC [1]. Therefore, the failure to find ESTs corresponding to Mac_ADH1.1 in the macaque EST database may reflect a lack EST sequences from fetal liver rather than a lack of Mac_ADH1.1 expression in fetal liver. SRA data for marmoset comes from the NCBI SRA database (Project ID 13630 by Washington University). The SRA and EST sequences were counted as a ‘hit’ against the reference sequence if they were ≥99% identical and >225 nucleotides long or ≥95% identical and >750 nucleotides long. This criteria generally distinguishes among the multiple paralogs, but occasionally results in a single transcript being counted as a ‘hit’ against more than one reference sequence. When this occurred, only the paralog with the highest scoring ‘hit’ was counted.

**References**

1. Magness, CL., Fellin, PC, Thomas, MJ, Korth, MJ, Agy, MB, Proll, SC, Fitzgibbon, M, Scherer, CA, Miner, DG, Katze, MG, Iadonato, SP (2005) Analysis of the Macaca mulatta transcriptome and the sequence divergence between Macaca and human. Genome Biol. 2005; 6(7): R60.
