# Supplementary material for: The Natural History of Class I Primate Alcohol Dehydrogenases Includes Gene Duplication, Gene Loss, and Gene Conversion
Source: PLoS One. 2012 Jul 31;7(7):e41175. doi: 10.1371/journal.pone.0041175 (PMC3409193; doi:10.1371/journal.pone.0041175)
Supplement: Table S5 — TREx estimates for paralog and ortholog ADH1 duplications. (DOC) [file pone.0041175.s022.doc]

**Table S5. TREx estimates for paralog and ortholog ADH1 duplications.**

| Paralog comparison | *f*_2_ | *kt* | Estimated time of divergence (millions of years) |
| --- | --- | --- | --- |
| Human ADH1A vs 1B | 0.94 | 0.134 | 4.31E+07 |
| Human ADH1A vs 1C | 0.9 | 0.234 | 7.54E+07 |
| Human ADH1B vs 1C | 0.94 | 0.134 | 4.31E+07 |
| Human ADH1A vs Chimp ADH1A | 0.99 | 0.021 | 6.79E+06 |
| Macaque ADH1.1 vs Macaque ADH1.2 | 0.98 | 0.04 | 1.37E+07 |
| Macaque ADH1.1 vs Macaque ADH1.3 | 0.94 | 0.13 | 4.31E+07 |
| Macaque ADH1.1 vs Macaque ADH1.4 | 0.92 | 0.18 | 5.88E+07 |
| Macaque ADH1.2 vs Macaque ADH1.3 | 0.94 | 0.13 | 4.31E+07 |
| Macaque ADH1.2 vs Macaque ADH1.4 | 0.92 | 0.18 | 5.88E+07 |
| Macaque ADH1.3 vs Macaque ADH .4 | 0.91 | 0.21 | 6.70E+07 |
| Marmoset ADH1.1 vs Marmoset ADH1.2 | 0.93 | 0.16 | 5.08E+07 |
| Marmoset ADH1.1 vs Marmoset ADH1.3 | 0.94 | 0.13 | 4.31E+07 |
| Marmoset ADH1.1 vs Marmoset ADH1.4 | 0.94 | 0.13 | 4.31E+07 |
| Marmoset ADH1.2 vs Marmoset ADH1.3 | 0.95 | 0.11 | 3.55E+07 |
| Marmoset ADH1.2 vs Marmoset ADH1.4 | 0.94 | 0.13 | 4.31E+07 |
| Marmoset ADH1.3 vs Marmoset ADH1.4 | 0.95 | 0.11 | 3.55E+07 |
| Human ADH1B vs Chimp ADH1B | 0.99 | 0.021 | 6.79E+06 |
| Human ADH1C vs Chimp ADH1C | 0.99 | 0.021 | 6.79E+06 |
| Human ADH1A vs Gibbon ADH1A | 0.96 | 0.087 | 2.81E+07 |
| Human ADH1B vs Gibbon ADH1B | 0.98 | 0.043 | 1.37E+07 |
| Human ADH1C vs Gibbon ADH1C | 0.95 | 0.110 | 3.55E+07 |
| Human ADH1A vs Macaque ADH1.1 | 0.95 | 0.110 | 3.55E+07 |
| Human ADH1A vs Macaque ADH1.2 | 0.93 | 0.158 | 5.08E+07 |
| Human ADH1A vs Macaque ADH1.3 | 0.91 | 0.208 | 6.70E+07 |
| Human ADH1A vs Macaque ADH1.4 | 0.9 | 0.234 | 7.54E+07 |
| Human ADH1B vs Macaque ADH1.1 | 0.94 | 0.134 | 4.31E+07 |
| Human ADH1B vs Macaque ADH1.2 | 0.94 | 0.134 | 4.31E+07 |
| Human ADH1B vs Macaque ADH1.3 | 0.93 | 0.158 | 5.08E+07 |
| Human ADH1B vs Macaque ADH1.4 | 0.93 | 0.158 | 5.08E+07 |
| Human ADH1C vs Macaque ADH1.1 | 0.91 | 0.208 | 6.70E+07 |
| Human ADH1C vs Macaque ADH1.2 | 0.92 | 0.182 | 5.88E+07 |
| Human ADH1C vs Macaque ADH1.3 | 0.93 | 0.158 | 5.08E+07 |
| Human ADH1C vs Macaque ADH1.1 | 0.95 | 0.110 | 3.55E+07 |
| Human ADH1A vs Marmoset ADH1.1 | 0.91 | 0.208 | 6.70E+07 |
| Human ADH1A vs Marmoset ADH1.2 | 0.94 | 0.134 | 4.31E+07 |
| Human ADH1A vs Marmoset ADH1.3 | 0.95 | 0.110 | 3.55E+07 |
| Human ADH1A vs Marmoset ADH1.4 | 0.92 | 0.182 | 5.88E+07 |
| Human ADH1B vs Marmoset ADH1.1 | 0.92 | 0.182 | 5.88E+07 |
| Human ADH1B vs Marmoset ADH1.2 | 0.93 | 0.158 | 5.08E+07 |
| Human ADH1B vs Marmoset ADH1.3 | 0.93 | 0.158 | 5.08E+07 |
| Human ADH1B vs Marmoset ADH1.4 | 0.94 | 0.134 | 4.31E+07 |
| Human ADH1C vs Marmoset ADH1.1 | 0.92 | 0.182 | 5.88E+07 |
| Human ADH1C vs Marmoset ADH1.2 | 0.92 | 0.182 | 5.88E+07 |
| Human ADH1C vs Marmoset ADH1.3 | 0.93 | 0.158 | 5.08E+07 |
| Human ADH1C vs Marmoset ADH1.4 | 0.92 | 0.182 | 5.88E+07 |
| Human ADH1A vs all lemur (averaged) | 0.86 | 0.345 | 1.11E+08 |
| Human ADH1B vs all lemur (averaged) | 0.87 | 0.316 | 1.02E+08 |
| Human ADH1C vs all lemur (averaged) | 0.89 | 0.260 | 8.40E+07 |
| vervet ADH1.1 vs vervetADH1.2 | 0.98 | 0.04 | 1.37E+07 |
| baboon ADH1.3 vs baboon ADH1.4 | 0.92 | 0.19 | 6.29E+07 |
| langur ADH1.3 vs langur ADH1.4 | 0.93 | 0.16 | 5.08E+07 |
| Macaque ADH1.1 vs Marmoset ADH1.1 | 0.92 | 0.182 | 5.88E+07 |
| Macaque ADH1.1 vs Marmoset ADH1.2 | 0.94 | 0.134 | 4.31E+07 |
| Macaque ADH1.1 vs Marmoset ADH1.3 | 0.94 | 0.134 | 4.31E+07 |
| Macaque ADH1.1 vs Marmoset ADH1.4 | 0.93 | 0.158 | 5.08E+07 |
| Macaque ADH1.2 vs Marmoset ADH1.1 | 0.91 | 0.208 | 6.70E+07 |
| Macaque ADH1.2 vs Marmoset ADH1.2 | 0.94 | 0.134 | 4.31E+07 |
| ADH1.2 vs Marmoset ADH1.3 | 0.93 | 0.158 | 5.08E+07 |
| ADH1.2 vs Marmoset ADH1.4 | 0.92 | 0.182 | 5.88E+07 |
| ADH1.3 vs Marmoset ADH1.1 | 0.9 | 0.234 | 7.54E+07 |
| ADH1.3 vs Marmoset ADH1.2 | 0.91 | 0.208 | 6.70E+07 |
| ADH1.3 vs Marmoset ADH1.3 | 0.91 | 0.208 | 6.70E+07 |
| ADH1.3 vs Marmoset ADH1.4 | 0.89 | 0.260 | 8.40E+07 |
| ADH1.4 vs Marmoset ADH1.1 | 0.93 | 0.158 | 5.08E+07 |
| ADH1.4 vs Marmoset ADH1.2 | 0.93 | 0.158 | 5.08E+07 |
| ADH1.4 vs Marmoset ADH1.3 | 0.92 | 0.182 | 5.88E+07 |
| ADH1.4 vs Marmoset ADH1.4 | 0.93 | 0.158 | 5.08E+07 |
| Human ADH1A vs treeshrew | 0.84 | 0.405 | 1.31E+08 |
| Human ADH1B vs treeshrew | 0.83 | 0.437 | 1.41E+08 |
| Human ADH1C vs treeshrew | 0.89 | 0.260 | 8.40E+07 |

The ‘*kt*’ values = -ln((*f_2_*-*Eq*)/(1-*Eq*)), where *f_2_* is the fraction of substitutions at two-fold degenerate sites in sequence pairs estimated according to the methods of Li, *et al*., 2006, and *Eq* is the codon bias at equilibrium, estimated for primates to be 0.52. The number of years separating two sequences, *t,* is calculated by dividing *kt* by the mutation rate *k,* (3.1 x 10^-9^ substitutions per site per year, see Li *et al*.*,* [1]. The estimated date of divergence (in millions of years ago) is *t*/2.

**References**

1. Li, T., Chamberlin, S. G., Caraco, M. D., Liberles, D. A., Gaucher, E. A., Benner, S. A. (2006) Analysis of transitions at two-fold redundant sites in mammalian genomes. Transition redundant approach-to-equilibrium (TREx) distance metrics. BMC Evolutionary Biology, 6, 241.
