# Supplementary material for: The Natural History of Class I Primate Alcohol Dehydrogenases Includes Gene Duplication, Gene Loss, and Gene Conversion
Source: PLoS One. 2012 Jul 31;7(7):e41175. doi: 10.1371/journal.pone.0041175 (PMC3409193; doi:10.1371/journal.pone.0041175)
Supplement: Table S6 — Pairwise distances among marmoset and macaque paralogs for individual introns and UTR regions. (DOC) [file pone.0041175.s023.doc]

**Table S6. Pairwise distances among marmoset and macaque paralogs for individual introns and UTR regions.**

|  | Pariwise distances among marmoset genomic comparisons | | | Summary of comparison |
| --- | --- | --- | --- | --- |
| Marmoset | | | | |
| 5’UTR (782-nt) | Cal_1.1 | Cal_1.2 | Cal_1.3 |  |
| Cal_1.2 | 0.199 |  |  | 1.2 closer to 1.1 than 1.3 |
| Cal_1.3 | 0.282 | 0.248 |  |  |
| Cal_1.4 | 0.219 | 0.213 | 0.205 |  |
| Intron 1 (3116-nt) | Cal_1.1 | Cal_1.2 | Cal_1.3 |  |
| Cal_1.2 | 0.152 |  |  | 1.2 closer to 1.1 than 1.3 |
| Cal_1.3 | 0.174 | 0.188 |  |  |
| Cal_1.4 | 0.157 | 0.183 | 0.156 * |  |
| Intron 2 (568-nt) | Cal_1.1 | Cal_1.2 | Cal_1.3 |  |
| Cal_1.2 | 0.170 |  |  | 1.2 closer to (≈) 1.1 than 1.3 |
| Cal_1.3 | 0.201 | 0.182 |  |  |
| Cal_1.4 | 0.231 | 0.242 | 0.230 * |  |
| Intron 3 (1726-nt) | Cal_1.1 | Cal_1.2 | Cal_1.3 |  |
| Cal_1.2 | 0.076 * |  |  | 1.2 closer to 1.1 than 1.3 |
| Cal_1.3 | 0.179 | 0.175 |  |  |
| Cal_1.4 | 0.182 | 0.183 | 0.182 |  |
| Intron 5 (1850-nt) | Cal_1.1 | Cal_1.2 | Cal_1.3 |  |
| Cal_1.2 | 0.155 |  |  |  |
| Cal_1.3 | 0.171 | 0.139 * |  | 1.2 closer, but approximately equal distance, to 1.3 than 1.1 |
| Cal_1.4 | 0.180 | 0.151 * | 0.170 * |  |
| Intron 6 (2112-nt) | Cal_1.1 | Cal_1.2 | Cal_1.3 |  |
| Cal_1.2 | 0.205 |  |  |  |
| Cal_1.3 | 0.186 | 0.131 * |  | 1.2 closer to 1.3 than 1.1 |
| Cal_1.4 | 0.192 | 0.195 | 0.161 * |  |
| Intron 7 (596-nt) | Cal_1.1 | Cal_1.2 | Cal_1.3 |  |
| Cal_1.2 | 0.167 |  |  |  |
| Cal_1.3 | 0.228 | 0.111 |  | 1.2 closer to 1.3 than 1.1 |
| Cal_1.4 | 0.198 | 0.123 * | 0.184 |  |
| Intron 8 (2528-nt) | Cal_1.1 | Cal_1.2 | Cal_1.3 |  |
| Cal_1.2 | 0.175 |  |  |  |
| Cal_1.3 | 0.169 | 0.142 |  | 1.2 closer to 1.3 than 1.1 |
| Cal_1.4 | 0.165 | 0.179 | 0.168 |  |
| 3’UTR (1816-nt) | Cal_1.1 | Cal_1.2 | Cal_1.3 |  |
| Cal_1.2 | 0.192 |  |  |  |
| Cal_1.3 | 0.204 | 0.158 |  | 1.2 closer to 1.3 than 1.1 |
| Cal_1.4 | 0.172 | 0.206 | 0.208 |  |
| Macaque | | | | |
| Intron 1 (3116-nt) | Mac_1.0 | Mac_1.1 | Mac_1.3 |  |
| Mac_1.1 | 0.105 * | 0.105 * | 0.105 * | 1.1 closer to 1.0 than 1.3 |
| Mac_1.3 | 0.180 | 0.180 | 0.180 |  |
| Mac_1.4 | 0.169 | 0.169 | 0.169 |  |
| Intron 2 (568-nt) | Mac_1.0 | Mac_1.1 | Mac_1.3 |  |
| Mac_1.1 | 0.078 * | 0.078 * | 0.078 * | 1.1 closer to 1.0 than 1.3 |
| Mac_1.3 | 0.151 | 0.151 | 0.151 |  |
| Mac_1.4 | 0.166 | 0.166 | 0.166 |  |
| Intron 3 (1726-nt) | Mac_1.0 | Mac_1.1 | Mac_1.3 |  |
| Mac_1.1 | 0.095 * | 0.095 * | 0.095 * | 1.1 closer to 1.0 than 1.3 |
| Mac_1.3 | 0.152 | 0.152 | 0.152 |  |
| Mac_1.4 | 0.175 | 0.175 | 0.175 |  |
| Intron 5 (1850-nt) | Mac_1.0 | Mac_1.1 | Mac_1.3 |  |
| Mac_1.1 | 0.138 | 0.138 | 0.138 |  |
| Mac_1.3 | 0.150 * | 0.150 * | 0.150 * | 1.1 closer, but approximately equal distance, to1.0 than 1.3 |
| Mac_1.4 | 0.150 | 0.150 | 0.150 |  |
| Intron 6 (2112-nt) | Mac_1.0 | Mac_1.1 | Mac_1.3 |  |
| Mac_1.1 | 0.154 | 0.154 | 0.154 |  |
| Mac_1.3 | 0.158 | 0.158 | 0.158 | 1.1 closer to 1.3 than 1.0 |
| Mac_1.4 | 0.164 * | 0.164 * | 0.164 * |  |
| Intron 7 (596-nt) | Mac_1.0 | Mac_1.1 | Mac_1.3 |  |
| Mac_1.1 | 0.180 | 0.180 | 0.180 |  |
| Mac_1.3 | 0.185 | 0.185 | 0.185 | approximately equal distance among 1.1, 1.0, and 1.3 |
| Mac_1.4 | 0.171 * | 0.171 * | 0.171 * |  |
| Intron 8 (2528-nt) | Mac_1.0 | Mac_1.1 | Mac_1.3 |  |
| Mac_1.1 | 0.158 | 0.158 | 0.158 |  |
| Mac_1.3 | 0.173 | 0.173 | 0.173 | 1.1 closer to 1.3 than 1.0 |
| Mac_1.4 | 0.155 | 0.155 | 0.155 |  |

Gene pairs potentially affected by gene conversions (as indicated in Table 3 and S7) are indicated by an asterisk.
