# Supplementary material for: The Natural History of Class I Primate Alcohol Dehydrogenases Includes Gene Duplication, Gene Loss, and Gene Conversion
Source: PLoS One. 2012 Jul 31;7(7):e41175. doi: 10.1371/journal.pone.0041175 (PMC3409193; doi:10.1371/journal.pone.0041175)
Supplement: Table S7 — Putative gene conversions of ancient ancestry. (DOC) [file pone.0041175.s024.doc]

**Table S7. Putative gene conversions of ancient ancestry.**

|  |  | evidence supporting putative gene conversion | | | | | |
| --- | --- | --- | --- | --- | --- | --- | --- |
| Paralogs involved in gene conversion | Region of multiple sequence alignment involved in gene conversion; comment | Similarity plots | Micro-indels | RDP | GENECONV | Boot-scanning | Corroborating adjacent exon/intron |
| **Macaque** |  |  |  |  |  |  |  |
| Mac_ADH1.2/1.3 and 1.4 | Intron 1 and 2, bases 3050 – 3150; Similarity is < 96% (only 93% in short region); potentially homologous GC with marmoset intronic region 3100. |  |  | X | X |  |  |
| Mac_ADH1.0 and 1.3 | Intron 5, bases 5900 – 6100; Similarity is = 96%, but no support from computational methods. | X |  |  |  |  |  |
| Mac_ADH1.2 and 1.4 | Intron 6, bases 8700 – 8800; Similarity is < 96% (only 93% in short region). |  |  | X | X |  |  |
| Mac_ADH1.0 and 1.4 (possibly also Mac_ADH_1.0 and 1.1 or Mac_ADH1.1 and 1.4) | Intron 6, bases 9250 – 9400 ; Similarity is < 96% (only 95%). |  |  | X | X | X |  |
| Mac_ADH1.1 and 1.2;  Mac_ADH1.1 and 1.3 | **Exon 8;** No support from computational methods, but three exonic homoplasies; potentially homologous gene conversion with human exon 8, indicating gene conversion that predates macaque-human split. | X |  |  |  |  |  |
| **Human** |  |  |  |  |  |  |  |
| Hum_ADH1B and 1C | **Exon 6;** potentially homologous gene conversion with macaque exon 6, indicating gene conversion that predates macaque-human split. | X | n/a | X |  |  |  |
| Hum_ADH1A and 1B | **Exon 8;** No support from computational methods, but four exonic homoplasies; potentially homologous gene conversion with macaque exon 8, indicating gene conversion that predates macaque-human split. | X |  |  |  |  |  |
| **Marmoset** |  |  |  |  |  |  |  |
| Cal_ADH1.3 and 1.4 | Intron 1 and 2, bases 3050-3150; Similarity is < 96% (only 92% in short region); potentially homologous gene conversion with macaque intronic region 3050-3150. |  |  | X | X | X |  |
| Cal_ADH1.3 and 1.4 | Intron 5, bases 6000 – 6200; Similarity is < 96% (only 92%). |  | X | X |  | X |  |
| Cal_ADH1.2 and 1.4 | Intron 5, bases 6300 – 6500; Similarity is < 96% (only 92%). |  |  | X | X | X |  |
| Cal_ADH1.2 and 1.3 | Intron 5 and 6, bases 7200-8000; Similarity is < 96% (only 92% - 95% in short region). |  |  | X | X | X |  |
|  | **Exon 6;** No support from computational methods, but corroborating gene conversion in neighboring intron; many homoplasies within exonic region. |  |  |  |  |  | Yes, neighboring intronic |
| Cal_ADH1.2 and 1.4 | **Exon 8;** No support from computational methods, but corroborating gene conversion in neighboring intron. | X | n/a |  |  |  | Yes, Intronic 9750 – 10,500 |
