# Supplementary material for: The Natural History of Class I Primate Alcohol Dehydrogenases Includes Gene Duplication, Gene Loss, and Gene Conversion
Source: PLoS One. 2012 Jul 31;7(7):e41175. doi: 10.1371/journal.pone.0041175 (PMC3409193; doi:10.1371/journal.pone.0041175)
Supplement: Text S1 — Molecular clocks applied to exonic regions do not favor one phylogeny over the other. (DOC) [file pone.0041175.s025.doc]

**Molecular clocks applied to exonic regions do not favor one phylogeny over the other.**

In principle, molecular clocks that estimate the dates of the paralog duplication from exonic data might be compared with dates for the platyrrhine-catarrhine divergence date estimated from a fossil record. The comparison might help distinguish the two alternative phylogenies.

Pursuing this rationale, we applied to the *ADH1* family the TREx clock of Li *et al*. [1]. The TREx clock estimates dates of divergence by examining two-fold redundant sites in coding regions where the encoded amino acid is conserved. Li *et al.* [1] calibrated the TREx clock in primates using an estimate of the primate-rodent divergence of 80 mya. They obtained a rate constant for silent transitions in two fold redundant sites of 3.1 x 10^-9^ substitutions/silent site/year for primates.

For this work, we re-calibrated the clock using the *ADH4* genes from various haplorhines, genes that are undisputed orthologs. From the fossil record, we used the lower and upper bounds for divergence of *Hominodea* and *Cercopithecoidea* of 23.0 to 33.9 mya [2, 3].

The fraction of two-fold redundant sites that are identical (*f*_2_) in pairwise comparisons of the *ADH4* orthologs was found to be ca. 0.94. This gives a TREx distance of 0.1335 and a rate constant ranging from 2.90 x 10^-9^ substitutions/silent site/year to 1.97 x 10^-9^ substitutions/silent site/year, depending on whether the more or less recent divergence date is taken from the fossil record. The first rate constant is consistent with the calibration of Li *et al.* [1].

Applying these two rate constants to the *ADH4* genes from New and Old World primates (*f*_2_ ≈ 0.90; TREx distance = 0.2336) gave dates of divergence that ranged from 40 to 59 mya, with the 40 mya date obtained with the larger rate constant closest to that obtained by Li *et al.* [1]. This is generally consistent with estimates based on the fossil record [4].

We then compared these to *f*_2_ values for the *ADH1* paralogs (Table S5). For example, in human-human paralog comparisons (A/B, A/C, and B/C), *f*_2_ values are 0.94, 0.90, and 0.94. Macaque-macaque paralog comparisons (excluding the Mac_*ADH1.2* and *1.3* pair because of extensive gene conversion, see primary text) ranged from 0.91 to 0.94. Marmoset-marmoset paralog comparisons gave *f*_2_ values from 0.93 to 0.95. The inter-taxa comparisons of marmoset and catarrhine orthologs were comparable, with *f*_2_ values ranging from 0.91 to 0.94 (orthologous pairs were assigned based on the intron tree).

Thus, the *f*_2_ values between *ADH1* paralogs were the same as those of *ADH1* orthologs. As these *f*_2_ values have substantial variances, the test was indecisive regarding whether the *ADH1* paralog duplication pre-dates the marmoset-catarrhine divergence.

All molecular clocks will be perturbed by gene conversion to give underestimates of the true duplication dates. This may explain why TREx distances for human-marmoset and macaque-marmoset ortholog comparisons are consistently lower between *ADH1* orthologs than between *ADH4* orthologs (0.1335 to 0.2076 for *ADH1* orthologs compared to 0.2336 for *ADH4* orthologs). In the case of *ADH1*, TREx dates are probably more perturbed than dates based on intronic sequences, because TREx dates involve far fewer sites (many of which are homoplasic). We therefore find the analysis of the intronic regions a more reliable estimate of the relative date of paralog duplication.

**References**

1. Li, T., Chamberlin, S. G., Caraco, M. D., Liberles, D. A., Gaucher, E. A., Benner, S. A. (2006) Analysis of transitions at two-fold redundant sites in mammalian genomes. Transition redundant approach-to-equilibrium (TREx) distance metrics. BMC Evolutionary Biology, 6, 241.

2. Benton MJ, Donoghue PCJ (2007) Paleontological evidence to date the tree of life. Mol Biol Evol 24: 26-53.

3. Donoghue PCJ, Benton MJ (2007) Rocks and clocks: calibrating the Tree of Life using fossils and molecules. Trends in Ecology & Evolution 22: 424-431.

4. Kay RF, Fleagle JG, Mitchell TRT, Colbert M, Bown T, Powers DW (2008) The anatomy of *Dolichocebus gaimanensis*, a stem platyrrhine monkey from Argentina. J Evol. 54: 323-382.
