# Supplementary material for: The Natural History of Class I Primate Alcohol Dehydrogenases Includes Gene Duplication, Gene Loss, and Gene Conversion
Source: PLoS One. 2012 Jul 31;7(7):e41175. doi: 10.1371/journal.pone.0041175 (PMC3409193; doi:10.1371/journal.pone.0041175)
Supplement: Text S2 — How many ADH1 paralogs existed in the urprimate? (DOC) [file pone.0041175.s026.doc]

**How many *ADH1* paralogs existed in the urprimate?**

With the historical controversies resolved following consideration of multiple sources of data and database error, we conclude that the last common ancestor of catarrhines and platyrrhines contained four *ADH1* paralogs. Further, our sequence data confirms that multiple *ADH1* genes exist in lemurs (strepsirhines), with as many as four in at least some lemur genera (e.g. ring-tailed lemur). This raised the question whether the multiple *ADH1* paralogs in the haplorhine ancestor and the multiple paralogs in lemurs indicate that their last common ancestor (the "urprimate") also had multiple *ADH1* paralogs.

Molecular clock estimates of *ADH1* intronic regions indicated four paralog duplications occured in the anthropoid ancestor before the catarrhines and platyrrhines diverged, but after the haplorhines diverged from the strepsirhines (Figure 3).

Similarly, the exonic sequence data from lemurs indicate only a single *ADH1* existed in the ancestor of strepsirhines and haplorhines (Figure S2). Only limited intron sequence data are available from strepsirhine *ADH1* genes, but they also indicate independent *ADH1* duplications within the haplorhine and strepsirhine lineages (Figure S4).

Depending on the root of the phylogeny in Figure S4, this data suggests at least two models: (1) the urprimate had a single *ADH1* paralog, where the multiple paralogs in the haplorhines and strepsirhines arose via independent duplications in the haplorhine and strepsirhine lineages, or (2) the urprimate had multiple *ADH1* paralogs, all but one of these was then lost in its haplorhine descendent, and the sole surviving *ADH1* paralog in the last common ancestor of catarrhines and platyrrhines gave rise to multiple duplicates just before catarrhines and platyrrhine split. Scenario (1) is more parsimonious, and the model we accept until additional lemur and tree shrew genome sequence data permits more detailed study. Both scenarios indicate extensive paralog duplication in the Paleocene or Eocene, a period just following the K/T boundary and late Cretaceous, a period of time when both paleontology and paleogenetics suggest that ethanol was becoming abundant in the biosphere.

A more comprehensive analysis of *ADH1* phylogeny in strepsirhines awaits the collection of more strepsirhine intron sequences. Even here, however, it should be recognized that models invoking lost genomic data are difficult to confirm until sequence data are obtained from other divergent primates. Furthermore, even if multiple paralogs existed in the urprimate ancestor and gave rise to (at least some of) the multiple paralogs in both lemur and haplorrhines, prevalent gene conversion involving intronic regions could potentially obscure this history.
